# Translation of Monoclonal Antibodies Pharmacokinetics from Animal to Human Using Physiologically Based Modeling in Open Systems Pharmacology (OSP) Suite: A Retrospective Analysis of Bevacizumab

**DOI:** 10.3390/pharmaceutics15082129

**Published:** 2023-08-14

**Authors:** Blaise Pasquiers, Salih Benamara, Mathieu Felices, David Ternant, Xavier Declèves, Alicja Puszkiel

**Affiliations:** 1 Inserm UMR-S1144, Faculty of Pharmacy, Université Paris Cité, 75006 Paris, Francealicja.puszkiel@aphp.fr (A.P.); 2 PhinC Development, 91300 Massy, France; 3 Faculty of Medicine, Université de Tours, EA 4245 T2I, 37032 Tours, France; 4 Service de Pharmacologie Médicale, CHRU de Tours, 37000 Tours, France; 5 Biologie du Médicament—Toxicologie, Hôpital Cochin, Assistance Publique Hôpitaux de Paris, 75014 Paris, France

**Keywords:** translational PBPK, monoclonal antibody, bevacizumab, TMDD

## Abstract

Interspecies translation of monoclonal antibodies (mAbs) pharmacokinetics (PK) in presence of target-mediated drug disposition (TMDD) is particularly challenging. Incorporation of TMDD in physiologically based PK (PBPK) modeling is recent and needs to be consolidated and generalized to provide better prediction of TMDD regarding inter-species translation during preclinical and clinical development steps of mAbs. The objective of this study was to develop a generic PBPK translational approach for mAbs using the open-source software (PK-Sim^®^ and Mobi^®^). The translation of bevacizumab based on data in non-human primates (NHP), healthy volunteers (HV), and cancer patients was used as a case example for model demonstration purpose. A PBPK model for bevacizumab concentration-time data was developed using data from literature and the Open Systems Pharmacology (OSP) Suite version 10. PK-sim^®^ was used to build the linear part of bevacizumab PK (mainly FcRn-mediated), whereas MoBi^®^ was used to develop the target-mediated part. The model was first developed for NHP and used for a priori PK prediction in HV. Then, the refined model obtained in HV was used for a priori prediction in cancer patients. A priori predictions were within 2-fold prediction error (predicted/observed) for both area under the concentration-time curve (AUC) and maximum concentration (C_max_) and all the predicted concentrations were within 2-fold average fold error (AFE) and average absolute fold error (AAFE). Sensitivity analysis showed that FcRn-mediated distribution and elimination processes must be accounted for at all mAb concentration levels, whereas the lower the mAb concentration, the more significant the target-mediated elimination. This project is the first step to generalize the full PBPK translational approach in Model-Informed Drug Development (MIDD) of mAbs using OSP Suite.

## 1. Introduction

The use of pharmacokinetic (PK) modelling during drug development has markedly increased, especially to predict the dose-concentration-response relationship in human models based on animal studies and to anticipate doses that should be investigated in upcoming clinical phases. Modelling methods and softwares were especially designed for small molecules, with the implementation of mechanistic data regarding drug transporters and metabolizing enzymes. However, many limitations remain in translational modelling of therapeutic proteins such as monoclonal antibodies (mAbs). Due to their large molecular weight, the mAbs volume of distribution is limited to a few liters represented by the vascular and interstitial spaces of highly perfused and leaky tissues, as antibody diffusion in tissues is allowed by the convective transport through paracellular pores in vascular endothelial cell membranes [1,2]. The non-specific elimination of mAbs consists in proteolysis in lysosomes after either target-independent fluid-phase or receptor-mediated endocytosis. The affinity of mAbs belonging to G-isotype immunoglobulins (IgG) to neonatal Fc receptor (FcRn) is the main receptor-mediated endocytosis mechanism that protects them from lysosomal degradation and explains their long elimination half-life in humans (11–30 days) while FcRn-mediated transcytosis is also involved in mAbs tissue distribution. mAb PK also depend on their interaction with target antigens (soluble and/or expressed on cell membranes). This phenomenon refers to target-mediated drug disposition (TMDD) and is responsible for non-linear PK profiles observed at lower concentrations when the target antigen is not saturated. TMDD depends on several factors such as the binding affinity, antigen turnover, elimination rate of the mAb-antigen complex, and finally on the mAb concentration. In case of drug development, inclusion of TMDD processes in the model could help in studying the PK/PD relationship that depends on antigen concentration in target tissues and provides a better evaluation of doses for further preclinical and clinical trials. However, the expression of target antigen is more complex than a simple one-compartment turnover that is assumed in TMDD modeling [3]. PBPK has the advantage to provide a more in-depth description of mAb biodistribution and may therefore account for mAb-target interaction not only in blood, but also in tissues and organs [4]. In addition, PBPK models consider the expression of FcRn in different tissues and mAb-FcRn binding affinity [5].

Although several PBPK models for mAbs disposition in humans [6,7] or in animals [5,8] have been reported in the literature, the methodology regarding the PK translation from animal to human including TMDD using full PBPK modeling remains limited. Glassman and Balthasar previously proposed a translational PBPK model including TMDD for mAbs scaled up from non-human primate (NHP) to human in the ADAPT software [9]. Their model was developed for mAbs exhibiting TMDD both in animals and humans. Nevertheless, not all mAbs show target-mediated profiles in animals and in such cases, prediction of PK in humans is more challenging. In addition, first-in-human (FIH) studies of mAbs can be also performed in healthy volunteers. Therefore, in the drug development process, the PK of mAbs should be first scaled up from preclinical species to healthy volunteers and then to patients. It could help to define the parameters related to TMDD in human and better predict the PK/PD relationship to avoid administering subtherapeutic doses in patients. In light of the above considerations, the objective of this study was to develop a full translational PBPK modelling approach including a TMDD mechanism for mAbs using the Open Systems Pharmacology (OSP) Suite. This modeling approach demonstration was based on a bevacizumab case example, using data from the literature. The model was used for a priori predictions of PK in human (healthy volunteers then patients) using monkey data during a theoretical drug development process.

## 2. Material and Methods

### 2.1. PK Data

Bevacizumab was selected for this retrospective analysis as it shows non-linear PK at very low doses due to TMDD and numerous PK studies in NHP, healthy volunteers (HV), and adult cancer patients after intravenous doses are available in the literature. Bevacizumab is a humanized monoclonal IgG1 antibody targeting vascular endothelial growth factor (VEGF) which results in blocking the angiogenesis and is approved for the treatment of several solid tumors [10]. Only complete PK profiles after intravenous doses were selected for this study to exclude potential sources of variability in subcutaneous absorption and to focus on drug distribution and elimination phases. Ten publications were selected, and data are summarized in Table 1. The majority of the data consisted of mean PK profiles (with or without standard deviation) although some individual profiles were also included. Data at 0.1 mg/kg dose from Gordon et al. [11] were excluded from this retrospective analysis due to large variability in the individual PK parameters reported in five patients (8- and 7-fold variation in clearance [CL] and area-under-the-concentration-time curve [AUC], respectively) and absence of individual PK profiles. The majority of the data were obtained in Caucasian populations, except for the study described by Li et al. in a Chinese population [12]. PK data were digitalized using the WebPlotDigitizer tool (https://apps.automeris.io/wpd/, accessed on 1 April 2022).

### 2.2. Software

PBPK modeling was performed using Open Systems Pharmacology (OSP) Suite version 10 (http://www.opensystemspharmacology.org, accessed on 1 April 2022) including the PBPK software PK-sim^®^. MoBi^®^ was used to extend the PBPK model with the specific TMDD process. Data processing and graph plotting were performed in R version 4.0.5 coupled with RStudio version 1.4.1.

### 2.3. PBPK Model Structure

**Figure 1:**
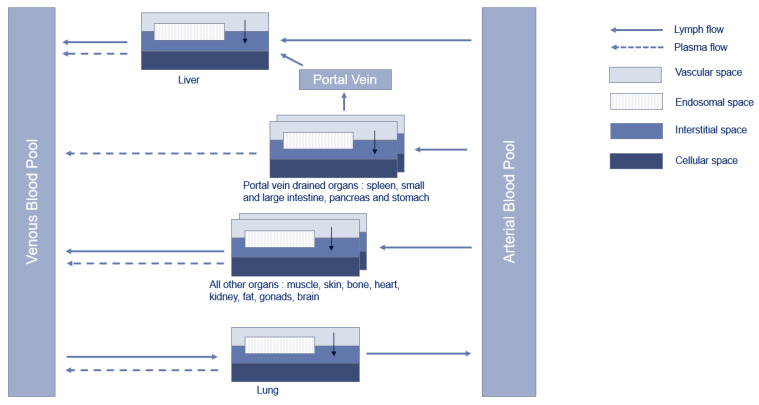
Schematic representation of the PBPK model in PK-Sim^®^, adapted from Niederalt et al. [21].

The PBPK model for mAbs in PK-sim^®^ was proposed by Niederalt et al. [21]. In this analysis, the generic model was used without further modifications with physiological values for NHP and humans implemented in the software [22,23,24]. The structure of the model consists of 15 compartments representing organs or tissues connected by blood and lymph flows. Each compartment was further divided into vascular (plasma and blood cells), endosomal, interstitial, and cellular spaces [21]. An additional compartment was added to represent endosomes and lysosomes within vascular endothelial cells [21] to account for the catabolism of the drug in the endosomal space and its binding to FcRn [7]. Schematic representation of the model is presented in Figure 1. The transcapillary exchange of the drug between plasma and interstitial space by convection or diffusion in each organ was described using a two-pores formalism [21]. The return of the drug from the interstitial space to the blood compartment was described by organ-specific lymph flow.

**Figure 2:**
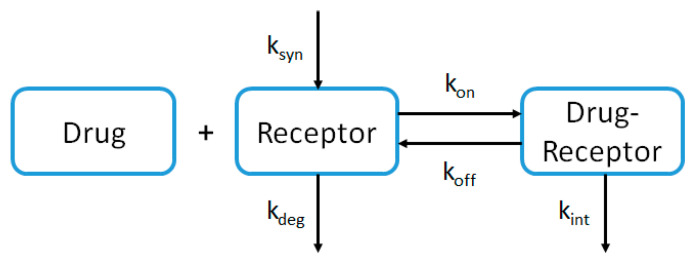
Schematic representation of the target-mediated drug disposition (TMDD) mechanism.

Since the PBPK model for proteins in PK-sim^®^ does not describe parameters related to the TMDD, the model was extended in Mobi^®^ software. TMDD describes the reversible binding of the drug with its receptor leading to internalization of the complex and its elimination (Figure 2). In the modeling analysis, TMDD was described as follows:

$$\frac{dR}{dt}=k_{syn}-k_{deg}\times R-k_{on}\times D\times R+k_{off}\times DR$$

$$\frac{dD}{dt}=-k_{on}\times D\times R+k_{off}\times DR$$

$$\frac{dDR}{dt}=k_{on}\times D\times R-k_{off}\times DR-k_{int}\times DR$$

$$\mathrm{With}\;k_{syn}=k_{deg}\times R_{0}$$

$$k_{on}=\frac{k_{off}}{K_{D,target}}$$

where R is the receptor concentration, R_0_ is the receptor concentration at steady-state, D is the drug concentration, DR is the drug-receptor complex concentration, k_syn_ is the zero-order synthesis rate constant of the receptor, k_deg_ is the first-order elimination rate constant of the receptor, k_on_ is the first-order binding rate constant, k_off_ is the first-order dissociation rate constant, k_int_ is the first-order internalization rate constant (elimination of the drug-receptor complex), and K_D,target_ is the equilibrium constant dissociation of DR. The TMDD mechanism was described in presence of the target in the extracellular space (plasma and interstitial fluid).

### 2.4. PBPK Modeling Approach

This retrospective PK analysis was performed using bevacizumab PK data from the literature to represent different steps of a theoretical development of a therapeutic protein. To evaluate the interest of this type of Model-Informed Drug Development (MIDD), the approach was divided in several sequential steps. The PBPK model for bevacizumab was first built for NHP, then refined based on observed PK data in NHP. The next step consisted in a priori prediction of PK profiles in HV with the NHP model and then the model was refined based on the observed data. Finally, the PBPK model in HV was used for a priori prediction of PK profiles in cancer patients. At each step, the model predictive performance was evaluated by comparing observed versus a priori predicted (obtained with the model without refined parameters) AUC from the first until the last observed or predicted concentration, and the maximal concentration (C_max_).

The values for each specific parameter used in the bevacizumab PBPK model in each population are summarized in Table 2. They include equilibrium dissociation constant with FcRn (K_D_-FcRn), K_D,target_ (equilibrium constant dissociation of bevacizumab with VEGF-A), VEGF-A concentration in each population, and parameters related to TMDD (k_deg_, k_int_, k_off_) in human.

#### 2.4.1. Non-Human Primate

The PBPK model was first developed in NHP in order to characterize the linear part of bevacizumab PK in a relevant species. The starting point of the model was to describe the physiology of the cynomolgus monkey with parameters referenced in the software and the physicochemical characteristics of bevacizumab (MW: 150 kDa and hydrodynamic radius: 5.34 nm). At this step, the key parameter to optimize was the dissociation constant of the IgG Fc portion with FcRn (K_D_-FcRn). This parameter was fitted in the model based on the observed data in NHP. To verify if K_D_-FcRn value used in the model was consistent and to confirm the PK prediction in NHP, the model was applied to PK data of xtend-bevacizumab. Xtend-bevacizumab is a lead Fc variant (M428L/N434S) of bevacizumab with greater affinity to human FcRn [13]. Values of K_D_-FcRn measured by Biacore in that study were 2460 and 218 nmol/L for bevacizumab and xtend-bevacizumab, respectively, which translates into an 11-fold higher affinity of xtend-bevacizumab to FcRn compared to bevacizumab [13]. The parameters related to bevacizumab binding to NHP VEGF-A were added in the model in order to consider the possible impact on its tissue distribution. K_D,target_ and k_off_ values for NHP (0.032 nmol/L and 3.5 day^−1^, respectively) were obtained from Ren et al. based on in vitro assays using a surface plasma resonance (SPR) Biacore system with NHP VEGF-A [25]. Since no data about VEGF-A concentration in NHP are available in the literature, it was assumed to be equivalent to that in human.

#### 2.4.2. Healthy Volunteers

First, the model tested in NHP was used to predict PK profile in HV, considering that bevacizumab PK in HV is linear and that no TMDD occurs at physiological values of VEGF-A. Physiological characteristics were adapted to human using PK-Sim^®^ references. K_D_-FcRn was considered 2-fold higher in humans than in NHP as described in the literature for IgG1 [27]. The K_D,target_ and k_off_ values for human (0.058 nmol/L and 2.7 day^−1^, respectively) were obtained from Papadopoulos et al. by in vitro assays using the SPR Biacore system with human VEGF-A [26]. Different values of VEGF-A plasma concentration in humans were reported in the literature (range: 0–3 nmol/L [28,29,30,31,32]). To account for this uncertainty in the model, different values ranging from 0 to 3 nmol/L were tested and evaluated in terms of best data fit. Large-scale gene expression data from publicly available sources are implemented in the OSP suite to be used directly in PBPK model building [33]. Expressed sequence tags (EST) is a gene expression database for many antigens and proteins extracted from relevant files in the human section of UniGene (National Center for Biotechnology Information) [34,35]. This EST gene database was used for VEGF-A relative expression across different organs and tissues in the model.

Second, the observed PK data collected in HV were used to refine the model. In order to characterize the non-linear profile observed in HV at very low doses (0.5 and 1 mg/kg), a TMDD mechanism was implemented in the model as described in Figure 2 and in the PBPK Model Structure Section. This mechanism was implemented in tissues and organs where VEGF-A is expressed according to EST gene database (Figure S1 in Supplementary Material). k_off_ was fixed to an in vitro value (2.7 day^−1^ [26]), whereas k_deg_ and k_int_ were fitted based on observed data at 0.5 and 1 mg/kg doses, respectively.

Finally, the model was validated using data in HV (single administration of a 3 mg/kg IV dose of bevacizumab) from the study by Liu et al. [36]. Observed data were compared to simulated data and AAFE and AFE were calculated.

#### 2.4.3. Adult Cancer Patients

The PBPK refined model in HV was used to predict PK profiles in adult cancer patients. According to the literature, VEGF-A concentration in cancer patients is 2- to 10-fold higher than in HV in several solid tumor patients including breast, prostate, and colorectal cancer patients [29,30,31]. In the model for cancer patients, VEGF-A concentration was multiplied by these factors (2- and 10-fold increase in VEGF-A concentration was tested) in each tissue and organ where VEGF-A is expressed according to EST gene database (Figure S1 in Supplementary Material). Other parameters were considered equivalent to those in HV. PK data observed in adult cancer patients were used to refine the model. Estimated parameters relative to the TMDD mechanism (k_deg_, k_int_ and VEGF-A concentrations) were adjusted to obtain the final model.

### 2.5. Model Evaluation

At each step, PK prediction obtained with the a priori model (i.e., PK prediction in HV obtained with NHP model, PK prediction in cancer patients obtained with HV model) was compared with the observed data. This allows to evaluate the predictive performance of this approach in case of a prospective drug development when PK data in a specie/population to which the PK was scaled is not yet available. The model evaluation was performed by comparing graphically observed and predicted AUC from the time of the first to the last observed or predicted concentration and the maximal plasma concentration (C_max_).

The overall predictability of the a priori model was also evaluated in terms of precision using AAFE (average absolute fold error) and AFE (average fold error) according to the following equations [37,38]:

$$AAFE=10^{\;\frac{1}{N\;}\sum |\log\left(\frac{C_{Predicted}}{C_{Observed}}\right)|}$$

$$AFE=10^{\;\frac{1}{N\;}\sum \log\left(\frac{C_{Predicted}}{C_{Observed}}\right)}$$

The model validation and verification criteria were based on the guidelines intended for regulatory submissions (EMA and FDA) reported by Abduljalil et al. and Shebley et al. [39,40]. Predictions were considered as very accurate if they fell within the 0.80–1.25 error range, acceptable if within 0.50/0.80–1.25/2.00, and inaccurate if outside the two-fold error range (0.50–2.00).

### 2.6. Sensitivity Analysis

Sensitivity analysis was performed on parameters of interest (K_D_-FcRn, K_D,target_, VEGF-A concentration, k_deg_, k_int_, k_off_) using the PK-Sim^®^ or Mobi^®^ tool. Parameter sensitivity was analyzed for AUC over the dosing interval at steady-state (AUC_tau,ss_) and C_max_ at steady-state (C_max,ss_). The sensitivity of the PK parameters (S) is calculated as the ratio of the relative change of the PK parameter and the relative variation of the model parameter (fixed at 10%) as follows:

$$S=\frac{\Delta Po\;}{\Delta pi\;}\times \frac{pi\;}{Po\;}$$

where $\frac{\Delta Po\;}{Po\;}$ is the relative change of the PK parameter (AUC_tau,ss_ or C_max,ss_) and $\frac{\Delta Pi\;}{pi\;}$ is the relative variation of the model parameter.

## 3. Results

### 3.1. Non-Human Primate

**Figure 3:**
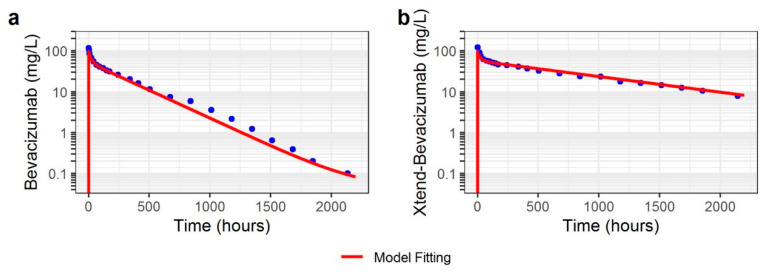
Observed and PBPK model-predicted concentrations in non-human primate: (**a**) 4 mg/kg IV dose of bevacizumab, (**b**) 4 mg/kg IV dose of xtend-bevacizumab.

Since no TMDD mechanism was necessary in the NHP model to fit the observed PK data, the key parameter in the model was K_D_-FcRn describing the affinity of bevacizumab to cynomolgus FcRn. This parameter was fitted to the observed data and the best fit was obtained with K_D_-FcRn of 450 nmol/L. This value was coherent with values found in the literature for IgG1 and bevacizumab in cynomolgus monkey [7,27]. Other parameters (such as concentration of VEGF-A, K_D,target_ and k_off_) were fixed to physiological or experimental values (Table 2). Concentration-time data were satisfactorily described by the model (Figure 3).

Then, the model was used to describe xtend-bevacizumab data. For that, the value of K_D_-FcRn was divided by 11 in the model for xtend-bevacizumab in comparison to bevacizumab (41 nmol/L, Table 2). The model adequately described xtend-bevacizumab data as shown in Figure 3. The details of the model development steps in PK-Sim^®^ are presented in Supplementary Material S2.

### 3.2. Healthy Volunteers

**Figure 4:**
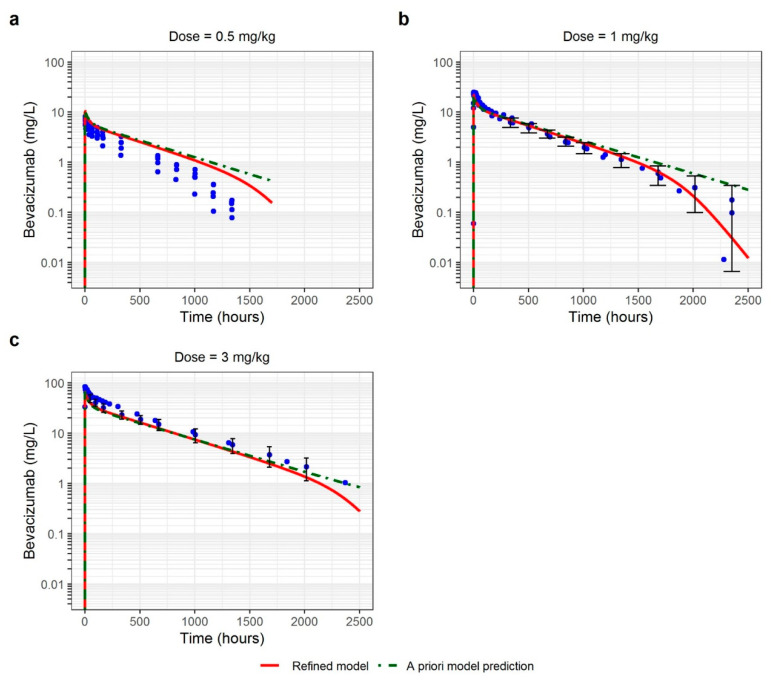
Observed and PBPK model-predicted concentrations in healthy volunteers for different intravenous doses: (**a**) 0.5 mg/kg; (**b**) 1 mg/kg; (**c**) 3 mg/kg. Green dashed curve represents a priori predictions of PK profiles using non-human primate model and red curve represents predictions obtained using refined model. Blue points are the observed data with their 95% confidence interval when available (black bars).

**Figure 5:**
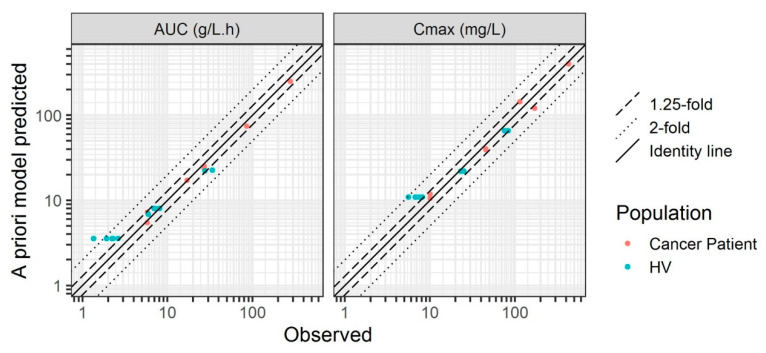
Observed versus a priori model-predicted AUC and C_max_ of bevacizumab. HV, healthy volunteers.

The PBPK model developed in NHP was used to predict PK in HV. A 2-fold higher K_D_-FcRn value than that in NHP (i.e., 900 nmol/L) was used in the model which allowed for adequate prediction of the observed data (Figure 4) [27]. The K_D,target_ was fixed to an in vitro value (0.058 nmol/L) [26]. The variation in VEGF-A concentration between 0 and 3 nmol/L had no impact on the predictions and was therefore fixed to a mid-range value (1.5 nmol/L). The model showed good prediction of the linear part of the PK, that is, at higher plasma concentrations (visible at 3 mg/kg and at the beginning of the curve at 1 mg/kg, Figure 4). However, a slight overprediction of the terminal slope at 1 mg/kg and of the entire PK profile at 0.5 mg/kg dose was observed. This overprediction was due to TMDD which was not integrated in the model at this step. However, this a priori PK prediction was quite good, with C_max_ and AUC within the 2-fold error threshold (except for one individual profile at 0.5 mg/kg dose in the Chinese population, Figure 5). For all the evaluated doses, AAFE and AFE ranged from 1.30 to 1.94 and from 0.72 to 1.94, respectively (Table 3). These PK profiles were in the acceptable range of prediction, with a slight overprediction trend for the 0.5 mg/kg dose. Parameter values are reported in Table 2. The details of the model development steps in PK-Sim^®^ are presented in Supplementary Material S3.

In the next step, the model was refined to improve the fitting of observed HV PK profiles. The K_D_-FcRn was adjusted from 900 to 940 nmol/L (Table 2). Then, the TMDD mechanism was implemented in the model. k_int_ and k_deg_ were fitted to the data at 0.043 day^−1^ and 0.18 day^−1^, respectively. The reference concentration of VEGF-A was adjusted to 0.3 nmol/L which allowed the description of TMDD Figure 4 (red profile). A slight overprediction of the entire PK profile was observed at 0.5 mg/kg dose. Nevertheless, the TMDD part of the model allowed a satisfactory description of these data.

The refined model was validated using data in HV (single administration of a 3 mg/kg IV dose of bevacizumab) from the study of Liu et al. [36]. Observed data were well predicted (Figure S2 in Supplementary Material) with AAFE and AFE of 1.24 and 1.23, respectively.

### 3.3. Adult Cancer Patients

**Figure 6:**
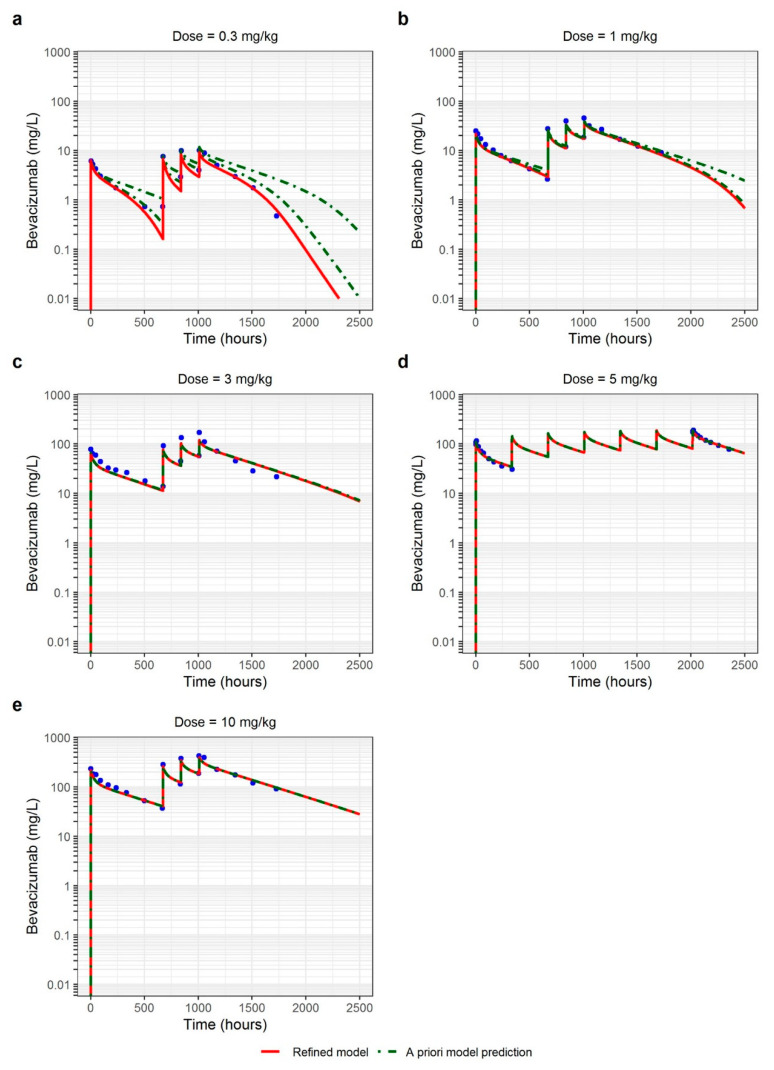
Observed and PBPK model-predicted concentrations in cancer patients at different intravenous doses: (**a**) 0.3 mg/kg; (**b**) 1 mg/kg; (**c**) 3 mg/kg; (**d**) 5 mg/kg; (**e**) 10 mg/kg. Green dashed curve represents a priori predictions of PK profiles using healthy volunteers’ model and red curve represents predictions obtained using refined model. Blue points are the observed data.

The PBPK model refined using HV data was used to predict PK profiles in adult cancer patients. The TMDD parameters k_deg_, k_int_, and k_off_ were assumed to be the same as in HV but it was considered that cancer patients have 2- to 10-fold higher concentrations of VEGF-A as described in the literature [29,30,31]. Therefore, different concentrations of VEGF-A were tested in the model (values ranging from 0.6 to 3 nmol/L corresponding to 2- to 10-fold variation compared to HV value). Increasing VEGF-A concentration resulted in higher bevacizumab CL and lower plasma concentrations. However, the impact of VEGF-A concentration on PK profiles was only visible for low doses (0.3 and 1 mg/kg), that is, in the presence of TMDD (Figure 6). The model showed good a priori predictive performance when compared to the observed data both in the linear and non-linear (TMDD) range of concentrations (especially when VEGF-A concentration was fixed to 3 nmol/L). Comparison of a priori PK predictions with the observed data is presented in Figure 5 and shows that the majority of the a priori predictions were within the 1.25-fold error threshold range and all the predictions were within the 2-fold error threshold range. AAFE and AFE ranged from 1.17 to 1.39 and from 0.75 to 1.04, respectively, for all the doses (Table 3). These PK profiles were mainly in the very accurate range of prediction, and in the acceptable range for 3 mg/kg C_max_. No trend of over- or underprediction could be observed.

Then, the observed data in adult cancer patients were used to fit TMDD model parameters to better describe the lowest observed concentrations which were overpredicted using the a priori model. The only fitted parameter was VEGF-A concentration (3.86 nmol/L). It allowed a better description of PK profiles regarding 1 and 3 mg/kg doses (Figure 6). This value is 12-fold higher than that in HV which is consistent with higher VEGF-A concentrations observed in cancer patients than in HV due to tumor burden and remains close to the value described in the literature [29,30,31]. The final parameter values are reported in Table 2.

### 3.4. Sensitivity Analysis

**Figure 7:**
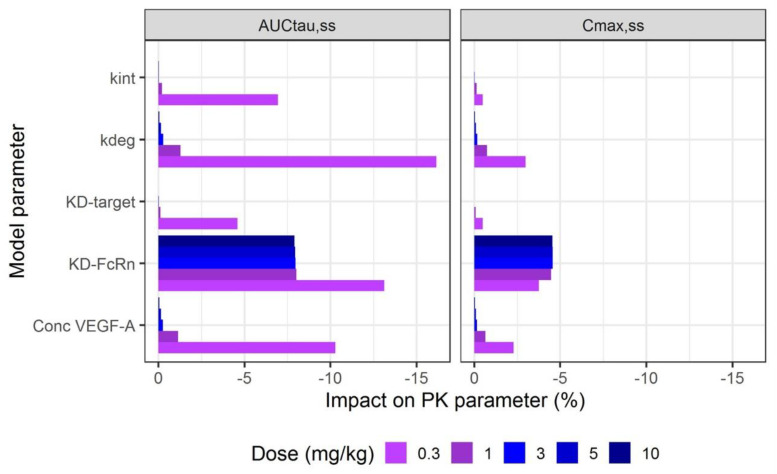
Results of the sensitivity analysis. k_int_: Internalization rate constant of the drug-target complex (1st order); k_deg_, degradation rate constant of VEGF (1st order); K_D,target_, equilibrium dissociation constant of bevacizumab to VEGF; K_D_-FcRn, equilibrium dissociation constant of bevacizumab to FcRn; Conc VEGF-A, concentration of VEGF.

Sensitivity analysis was performed for each fitted model and for each dose and the results are presented in Figure 7. K_D_-FcRn is the most important parameter to fit the linear part of the PK. Taking as an example the model in cancer patients and a 10 mg/kg dose, a 10% increase in K_D_-FcRn resulted in a decrease of 8% and 4.5% in AUC_tau,ss_ and C_max,ss_, respectively. Although the results show that K_D_-FcRn have an impact on PK profiles at each dose, parameters relative to the TMDD mechanism became more significant at lower doses. For a dose of 0.3 mg/kg in cancer patients, k_deg_ was the parameter with the highest impact on AUC_tau,ss_ and C_max,ss_, followed by K_D_-FcRn and VEGF-A concentration. At this dose, an increase of 10% in k_deg_ induced a 16% decrease in AUC_tau,ss_, whereas a 10% increase in VEGF-A concentration or K_D_-FcRn induced a decrease of 10% and 13% of AUC_tau,ss_, respectively. For HV models in single dose, only K_D_-FcRn impacted AUC_tau,ss_ with a similar effect at each dose. An increase of 10% in K_D_-FcRn induced an 8% decrease of AUC_tau,ss_.

## 4. Discussion

Translational PK of therapeutic proteins is usually performed using population approach when the drug shows only linear PK both in animal and in human [2]. However, this translation becomes more complex in case of non-linearity in PK regardless of its origin (e.g., TMDD, anti-drug antibodies [ADA], disease state). In such cases, more mechanistic translational models are needed, such as full PBPK or quantitative system pharmacology (QSP) models. Since this kind of translational tool is still little described in the literature, the interest of this work was to develop a full translational PBPK modeling approach in an open-source software (OSP Suite) based on literature data for bevacizumab.

The developed PBPK translational model showed good predictive performance at all steps of a theoretical drug development process. Interestingly, TMDD was observable in HV, especially at low doses. Since VEGF-A concentration is significantly lower in HV than in patients, this TMDD mechanism was not necessarily expected. According to the manufacturer label, bevacizumab PK is linear in HV for doses ranging from 1 to 10 mg/kg [10], although a slightly non-linear profile was visible at 1 mg/kg in several studies [14,15,16,17]. In addition, Li et al. used a mixed zero- and first-order model to describe elimination of bevacizumab at 0.5 mg/kg in HV as it provided better description of the data than a linear PK model [12]. In this study, the use of HV data allowed to better anticipate PK in cancer patients, mostly due to the description of TMDD processes. Although during this theoretical MIDD, PK in patients could have been anticipated directly from NHP data, no indication on k_deg_ and k_int_ parameters would have been available which could have limited model predictions. Therefore, this work supports the relevance of PK studies in HV during mAbs development. Phase 1 trials in patients are still a standard approach in the oncology field. However, as mAbs have a safer toxicity profile compared to conventional anticancer chemotherapy, it is more and more discussed to perform FIH studies in HV in the oncology field as well, which would help to avoid subtherapeutic doses in patients as discussed by Tranter et al. [41].

Previously, Baxter et al. [42] and Davda et al. [43] described the scaling up of linear mAbs PK using PBPK modeling. A platform PBPK model was also developed to describe the PK of mAbs including target-mediated elimination across several species by Shah and Betts [44], but no simulations were performed to analyze the ability of the model to predict PK in human. More recently, a catenary PBPK model developed by Chen and Balthasar [45] was scaled up to monkey [46] then to cancer patients [9]. That model showed good prediction of PK of four mAbs (cetuximab, dalotuzumab, figitumumab, and trastuzumab) but did not account for data in HV. Since FIH studies of mAbs are usually performed in HV, especially outside oncologic indications, the PK of mAbs in that case should be first scaled up from preclinical species to HV and then to patients. It could help to define the parameters related to TMDD in human and better predict the PK/PD relationship, especially at lower doses.

Parameter values obtained in cancer patients in the refitted model are consistent with those reported in the literature for bevacizumab using PBPK and population PK approaches [7,47,48]. However, the VEGF-A elimination half-life (i.e., 3.95 days, calculated as ln2/k_deg_) was higher than the value previously described in the literature based on in vitro assays (between 30 min and several hours [49,50,51]). Nevertheless, Papachristos et al. and Panoilia et al. reported similar values of VEGF-A elimination half-life estimated using a population approach in metastatic cancer patients (5.97 and 1.73 days, respectively) [47,48]. It could be explained by the fact that VEGF-A is largely bound to its receptor in the membrane which could slow down its elimination [49].

In our model, endosomal kinetics of mAb–FcRn complex was described with non-equilibrium differential equations as previously developed by Niederalt et al. [21] (details on model equations are presented in Supplementary Material S1). This non-equilibrium mAb-FcRn binding was already used in the catenary model by Chen and Balthasar [45]. Non-equilibrium mAb-FcRn binding predicts more modest changes in antibody elimination half-life due to modifications of the affinity to FcRn (K_D_-FcRn) than the equilibrium model [4,45], for example, a <2.5-fold change in half-life for a 10-fold increase in binding affinity [45]. We observed similar findings with our model, since the 11-fold increase in FcRn binding affinity of xtend-bevacizumab compared to bevacizumab led to a 3-fold decrease in elimination half-life, which is coherent with the observed data. Chen and Balthasar speculated that non-equilibrium binding could explain the lack of a clear relationship between equilibrium FcRn binding affinity at pH 6 and the observed in vivo half-life of IgG antibodies [45]. Indeed, because of the slow rates of dissociation of mAb with FcRn, the assumption that mAb–FcRn binding reaches equilibrium prior to endosomal sorting seems incorrect.

Overall, the developed PBPK modeling framework showed good predictive performance. All the a priori predictions were within a 2-fold threshold for PE, AAFE, and AFE compared to the observed data. Only one data set was slightly overpredicted (1.58, 1.78, 1.94 and 1.94-fold PE for C_max_, AUC, AAFE and AFE respectively). It corresponded to Chinese HV administered a 0.5 mg/kg dose (Figure 4 and Figure 5). This overprediction seems to be due to an inadequate description of the volume of distribution that could be related to a physiological difference in this ethnic group. However, in a population PK analysis by Han et al., the differences in bevacizumab PK between Asian and Caucasian patients were described solely by the difference in body weight [52], which was considered in our analysis. It suggests that there might be other ethnical differences in physiological parameters between Caucasian and Asian populations which were not well accounted for in the model.

Sensitivity analysis allowed to evaluate the importance of K_D_-FcRn and TMDD-related parameters on model predictions for different doses and populations (NHP, HV and cancer patients) and to understand which parameter needs to be optimized during development of mAbs PBPK model. As expected, the variation of TMDD parameters had the highest impact on PK profiles at the lowest doses (especially 0.3 mg/kg in cancer patients). Since TMDD parameters are important to predict PK in FIH phase 1 trials, these parameters should be obtained by in vitro assays and in vivo measurement of the target concentration in preclinical species and target populations prior to PBPK model building. Concerning K_D_-FcRn, this parameter is the most important to determine the linear part of mAbs PK. It is usually determined by in vitro assays prior to PBPK modelling. However, K_D_-FcRn found in vitro does not necessarily correspond to the in vivo value. The in vitro value is used in preclinical PBPK modelling as a starting point and the in vivo value is obtained by fitting the observed data. To anticipate K_D_-FcRn in human, the in vitro/in vivo ratio determined in animal and the in vitro value from SPR assays in human is used to develop a PBPK model. PK translation of rozanolixizumab from preclinical to clinical stages described in the study by Lledo-Garcia et al. is an example of this type of approach [53].

The translational PBPK modelling approach developed in this work could be extended to other antibodies using the databases integrated in OSP Suite. The ease of use of the OSP Suite for translational PBPK is the fact that the software requires limited number of in vitro data to predict PK profiles. Therefore, it can help a more rapid screening of mAb drug candidates as it is currently done for small molecule drugs. In addition, the use of a comprehensive software tool for whole-body PBPK enables rapid access to all relevant anatomical and physiological parameters for human and common laboratory animals (mouse, rat, minipig, dog, monkey and rabbit) which are available from an integrated database [54]. Also, the target expression in the PK-Sim^®^ EST database allows to easily develop linear or non-linear PK models for mAbs and to extrapolate inter-species PK with a reduced number of experimental data and with accurate predictions as shown in this example with bevacizumab. Finally, the PBPK model presented in this work can serve as a platform to develop models for other mAbs with more complex PK properties such as bi- or trispecific antibodies or antibody-drug conjugates (ADC). Indeed, in such cases, conventional population PK approaches might not allow to study the TMDD processes and PK-pharmacodynamic (PK/PD) relationship. Khot et al. used the example of T-DM1 (trastuzumab emtansine) to develop a full PBPK modelling platform in ADAPT software [55] whereas more recently, Zhang et al. described PK translation of a bispecific antibody using full PBPK approach in GastroPlus^®^ software [56]. Finally, translational QSP models have been developed for CD3 bispecific molecules [57]. Prediction of drug PK, efficacy and toxicity using mechanistic frameworks integrating in silico, in vitro and in vivo data stands for the future in the drug development especially in biologics which are more complex drugs than small molecules.

This study has several limitations. First, a prospective validation of this approach was not possible because no prospective bevacizumab data set was available in our study. To further validate and consolidate the use of this approach in MIDD of mAbs, other studies with mAbs with more complex PK (e.g., ADC, bi- or trispecific antibodies) are needed.

Other host-related factors such as disease state, tumor burden, and presence of ADA may impact on mAbs PK [1,2] and were not included in this analysis. Time-varying PK has been observed for several mAbs such as nivolumab and pembrolizumab and has been associated to changes in disease state (cachexia, inflammation) [58,59]. In addition, patients who present ADA can have higher mAbs CL [2]. Prediction of mAbs PK in patients using animal or HV data does not allow to take these factors into account which might result in a wrong dose selection. Thus, further improvements should be performed in the translational PBPK models in order to include these factors in PK predictions.

## 5. Conclusions

In this work, we report development of a PBPK modelling framework in OSP Suite for PK translation from NHP to cancer patients for a mAb presenting TMDD. This project is the first step to generalize this kind of approach in MIDD of mAbs using OSP suite. 

## Figures and Tables

**Table 1:** Summary of digitalized PK bevacizumab data used for the PBPK model development.

Population	Number of Subjects	Bevacizumab Dose	Study Description
Cynomolgus monkey	2 (bevacizumab) 2 (xtend-bevacizumab)	4 mg/kg Single dose	PK study in cynomolgus monkey, Mean profiles Zalevksy et al., 2010 [13]
HV	5	0.5 mg/kg Single dose	PK study of bevacizumab, Individual profiles in Chinese HV Li et al., 2017 [12]
HV	43	1 mg/kg Single dose	Phase I study in HV, Mean (sd) profiles Wu et al., 2019 [14]
HV	30	1 mg/kg Single dose	Phase I study in HV, mean profiles Hettema et al., 2017 [15]
HV	37	1 mg/kg Single dose	Phase I study in HV, Mean profiles Hummel et al., 2022 [16]
HV	39	1 mg/kg Single dose	Phase I study in HV, mean profiles Demarchi et al., 2021 [17]
HV	38	3 mg/kg Single dose	Phase I study in HV, Mean profiles Sinn et al., 2022 [18]
HV	40	3 mg/kg Single Dose	Phase I study in HV, Mean (sd) profiles Shin et al., 2020 [19]
Adult cancer patients	5 subjects per dose	0.3, 1, 3, 10 mg/kg Repeated dose	Phase I study in cancer patients, Mean profiles Gordon et al., 2001 [11]
Adult cancer patients	61	5 mg/kg Repeated dose	Biosimilar study in patients, Mean profiles Romera et al., 2018 [20]

HV, Healthy Volunteers, sd, standard deviation.

**Table 2:** Bevacizumab PBPK model parameters.

Parameter	Cynomolgus Monkey		Healthy Volunteers		Adult Cancer Patients	
	Bevacizumab	Xtend-Bevacizumab	A Priori Predicted	Refined	A Priori Predicted	Refined
K_D_-FcRn (nmol/L)	450	41	900	940	940	940
K_D,target_ ^a^ (nmol/L)	0.032 [25]	0.032 [25]	0.058 [26]	0.058 [26]	0.058 [26]	0.058 [26]
k_off_^a^ (day^−1^)	3.5 [25]	3.5 [25]	3.5 [26]	2.7 [26]	2.7 [26]	2.7 [26]
VEGF-A (nmol/L)	0 to 3 (min-max)	0 to 3 (min-max)	1.5	0.3	0.6 to 3 (min-max)	3.86
k_deg_ (day^−1^)	-	-	-	0.18	0.18	0.18
k_int_ (day^−1^)	-	-	-	0.043	0.043	0.043

K_D_-FcRn, equilibrium dissociation constant of FcRn-bevacizumab complex; K_D,target_, equilibrium dissociation constant of bevacizumab-VEGF-A complex; k_off_, first-order dissociation rate constant of bevacizumab-VEGF-A complex; VEGF-A, concentration of VEGF-A; k_deg_, first-order degradation rate constant of VEGF-A; k_int_, first-order internalization rate constant of bevacizumab-VEGF-A complex. ^a^ Parameter fixed to the literature value.

**Table 3:** AFE and AAFE evaluation for a priori predictions of adult cancer patients and healthy volunteers.

Population	Dose (mg/kg)	AFE	AAFE
Adult Cancer Patients	0.3	0.89	1.38
	1	0.75	1.39
	3	0.86	1.27
	5	1.04	1.17
	10	0.85	1.23
Healthy Volunteers	0.5	1.94	1.94
	1	1.05	1.30
	3	0.72	1.39

AAFE: average absolute fold error. AFE: average fold error.

## Data Availability

No new data were created in this study. Data sharing is not applicable to this article.

## Supplementary Materials

The following supporting information can be downloaded at: https://www.mdpi.com/article/10.3390/pharmaceutics15082129/s1. Supplementary Material S1. Details of the FcRn-mAb binding model from Niederalt et al. Supplementary Material S2. Details of the different steps of model development in non-human primate in PK-Sim^®^. Supplementary Material S3. Details of the different steps of model development in healthy volunteers in PK-Sim^®^. Figure S1. Relative expression of VEGF-A in tissues and organs in the full PBPK model in PK-Sim according to Expressed Sequence Tags (EST) database. Figure S2. Observed and PBPK model-predicted concentrations in healthy volunteers for a 3 mg/kg IV dose. Red curve represents predictions obtained using refined model. Blue points are the observed data. AAFE and AFE are 1.24 and 1.23, respectively.
