# Supplementary material for: Translation of Monoclonal Antibodies Pharmacokinetics from Animal to Human Using Physiologically Based Modeling in Open Systems Pharmacology (OSP) Suite: A Retrospective Analysis of Bevacizumab
Source: Pharmaceutics. 2023 Aug 14;15(8):2129. doi: 10.3390/pharmaceutics15082129 (PMC10459442; doi:10.3390/pharmaceutics15082129)
Supplement: Supplementary file 1 [file pharmaceutics-15-02129-s001.zip › Supplementary Material figrues and Supplementary Material S1.pdf]

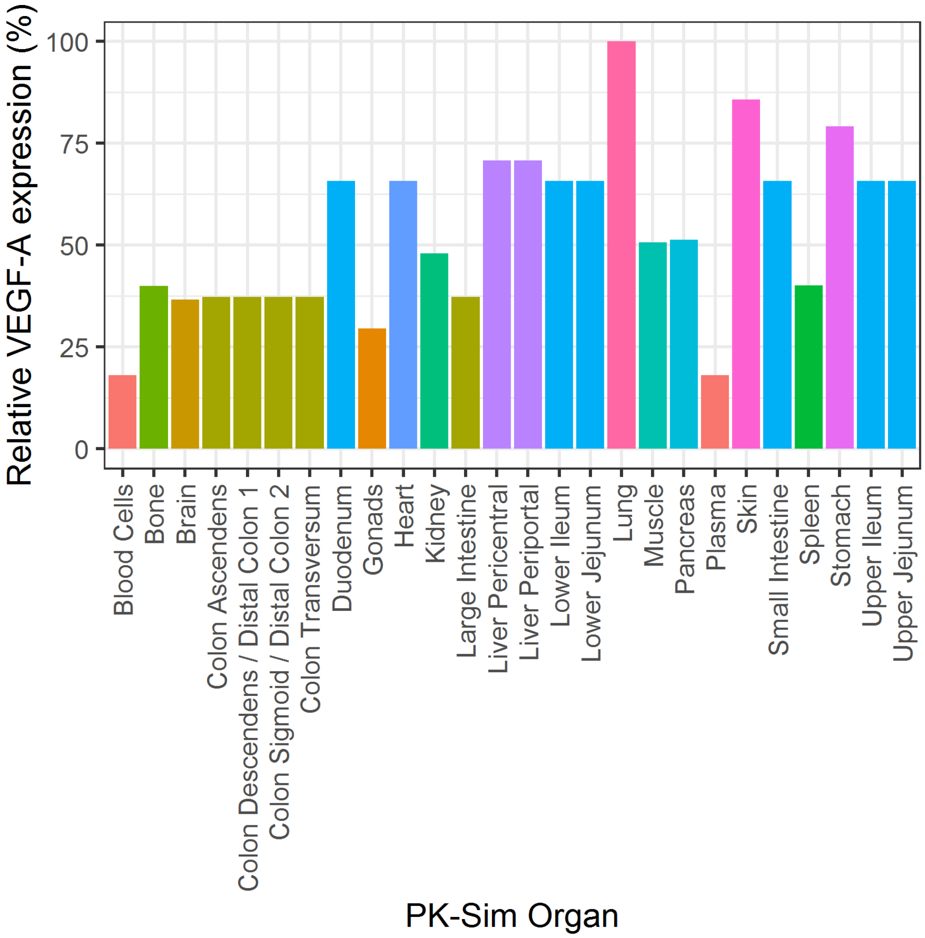

Figure S1. Relative expression of VEGF-A in tissues and organs in the full PBPK model in PK-Sim according to Expressed Sequence Tags (EST) database.

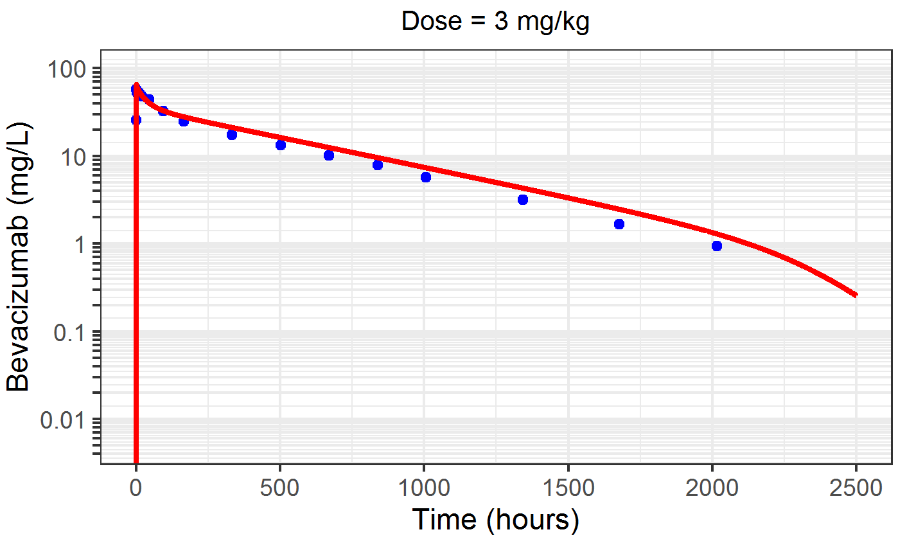

Figure S2. Observed and PBPK model-predicted concentrations in healthy volunteers for a 3 mg/kg IV dose. Red curve represents predictions obtained using refined model. Blue points are the observed data. AAFE and AFE are 1.24 and 1.23, respectively.

Supplementary Material S1. Details of the FcRn-mAb binding model from Niederalt *et al*. [1].

In the model, the specific CL of the drug not bound to FcRn from the endosomal space is calculated as the difference of the uptake and recycling rate constants kup – krec, thus CL from the endosomal space is given by the following equation:

$$\frac{dn^{drug}}{dt} = (k_{up} - k_{rec}) \cdot V_{end} \cdot C_{end}^{drug}$$

For the endogenous IgG sub-model, the rate equations are analogously to those given above for the drug. The FcRn binding reaction for the drug and the endogenous IgG in plasma, interstitial, or endosomal space is described by the following equations:

$$\frac{dC^{comp-FcRn}}{dt} = -\frac{dC^{comp}}{dt} = -\frac{dC^{FcRn}}{dt} = k_{ass} \cdot C^{comp} \cdot C^{FcRn} - k_d \cdot k_{ass} \cdot C^{comp-FcRn}$$

$$\frac{dn^{comp-FcRn}}{dt} = f_{vas}^{res} \cdot k_{rec} \cdot V_{end} \cdot C_{end}^{comp-FcRn}$$

where C_{comp} is the concentration of the drug in different organs or of endogenous IgG in the sub-model for the endogenous IgG/FcRn, C_{FcRn} is the concentration of FcRn in the sub-model for the endogenous IgG/FcRn, $C_{comp-FcRn}$ is the concentration of the FcRn – drug complex in different organs or endogenous IgG in the sub-model for the endogenous IgG/FcRn, k_{ass} is the association rate constant for FcRn binding and k_d is the dissociation constant for FcRn binding. $n^{comp-FcRn}$ is the amount of substance of the drug–FcRn or endogenous IgG–FcRn complex, f_{vas}^{res} is the fraction of recycling of the FcRn complex from endosomal space to plasma, k_{rec} is the recycling rate constant, V_{end} is the endosomal volume.

1. Niederalt C, Kuepfer L, Solodenko J, Eissing T, Siegmund HU, Block M, et al. A generic whole body physiologically based pharmacokinetic model for therapeutic proteins in PK-Sim. J Pharmacokinet Pharmacodyn. 2018;45(2):235-57.
