# Supplementary material for: Translation of Monoclonal Antibodies Pharmacokinetics from Animal to Human Using Physiologically Based Modeling in Open Systems Pharmacology (OSP) Suite: A Retrospective Analysis of Bevacizumab
Source: Pharmaceutics. 2023 Aug 14;15(8):2129. doi: 10.3390/pharmaceutics15082129 (PMC10459442; doi:10.3390/pharmaceutics15082129)
Supplement: Supplementary file 1 [file pharmaceutics-15-02129-s001.zip › Supplementary Material S2.pdf]

Project
Fit-singe

bpasquiers

August 3, 2023

Generated using PK-Sim® 10.0.257.

Contents

I Building Blocks 6

1 Individuals 7
1.1 monkey 7
1.1.1 Biometrics 7
1.1.2 Anatomy & Physiology 7
1.1.3 Expression 8
1.1.3.1 Metabolizing Enzymes 8
1.1.3.2 Transport Proteins 8
1.1.3.3 Protein Binding Partners 8
1.2 HV_sim 8
1.2.1 Biometrics 8
1.2.2 Anatomy & Physiology 9
1.2.3 Expression 9
1.2.3.1 Metabolizing Enzymes 9
1.2.3.2 Transport Proteins 9
1.2.3.3 Protein Binding Partners 9
1.3 HV_sim_VEGF 9
1.3.1 Biometrics 9
1.3.2 Anatomy & Physiology 10
1.3.3 Expression 10
1.3.3.1 Metabolizing Enzymes 10
1.3.3.2 Transport Proteins 10
1.3.3.3 Protein Binding Partners 10
1.4 monkey_VEGF 10
1.4.1 Biometrics 10
1.4.2 Anatomy & Physiology 11
1.4.3 Expression 11
1.4.3.1 Metabolizing Enzymes 11
1.4.3.2 Transport Proteins 11
1.4.3.3 Protein Binding Partners 11

2 Compounds 12
2.1 bevacizumab_monkey 12
2.1.1 Basic Physico-chemistry 12
2.1.1.1 Molecular weight 12
2.1.1.2 Lipophilicity 12
2.1.1.3 Fraction unbound (plasma, reference value) 12
2.1.1.4 Solubility 13
2.1.2 ADME 13
2.1.2.1 Absorption 13
2.1.2.2 Distribution 13
2.2 bevacizumab_xtend_monkey 13
2.2.1 Basic Physico-chemistry 13
2.2.1.1 Molecular weight 14
2.2.1.2 Lipophilicity 14
2.2.1.3 Fraction unbound (plasma, reference value) 14
2.2.1.4 Solubility 14

2.2.2 ADME . . . 14
2.2.2.1 Absorption . . . 14
2.2.2.2 Distribution . . . 15
2.3 Bevacizumab_human_sim . . . 15
2.3.1 Basic Physico-chemistry . . . 15
2.3.1.1 Molecular weight . . . 15
2.3.1.2 Lipophilicity . . . 15
2.3.1.3 Fraction unbound (plasma, reference value) . . . 16
2.3.1.4 Solubility . . . 16
2.3.2 ADME . . . 16
2.3.2.1 Absorption . . . 16
2.3.2.2 Distribution . . . 16
2.4 bevacizumab_monkey_VEGF . . . 17
2.4.1 Basic Physico-chemistry . . . 17
2.4.1.1 Molecular weight . . . 17
2.4.1.2 Lipophilicity . . . 17
2.4.1.3 Fraction unbound (plasma, reference value) . . . 17
2.4.1.4 Solubility . . . 18
2.4.2 ADME . . . 18
2.4.2.1 Absorption . . . 18
2.4.2.2 Distribution . . . 18

3 Protocols **20**
3.1 Perfusion 60 min_4 mg/kg . . . 20
3.2 Perfusion 90 min_1mg/kg . . . 20
3.3 Perfusion 90 min_3mg/kg . . . 21
3.4 Perfusion 90 min_0.5mg/kg . . . 21

II Simulations **22**

1 Monkey_beva_4mpk **23**
1.1 Used building blocks . . . 23
1.2 Simulation Properties . . . 23
1.2.1 Model Structure . . . 23
1.2.2 Compounds . . . 23
1.2.2.1 bevacizumab_monkey . . . 23
1.2.3 Administration . . . 24
1.2.3.1 bevacizumab_monkey . . . 24
1.3 Charts . . . 24
1.3.1 Time Profile Analysis . . . 25
1.3.1.1 Global PK-Analyses . . . 25
1.3.1.2 PK-Analyses . . . 25

2 Monkey_beva-xtend_4mpk **27**
2.1 Used building blocks . . . 27
2.2 Simulation Properties . . . 27
2.2.1 Model Structure . . . 27
2.2.2 Compounds . . . 27
2.2.2.1 bevacizumab_xtend_monkey . . . 27
2.2.3 Administration . . . 28
2.2.3.1 bevacizumab_xtend_monkey . . . 28
2.3 Charts . . . 28
2.3.1 Time Profile Analysis . . . 29
2.3.1.1 Global PK-Analyses . . . 29
2.3.1.2 PK-Analyses . . . 29

3 Sim_HV_1mg/kg 31
3.1 Used building blocks 31
3.2 Simulation Properties 31
3.2.1 Model Structure 31
3.2.2 Compounds 31
3.2.2.1 Bevacizumab_human_sim 31
3.2.3 Administration 32
3.2.3.1 Bevacizumab_human_sim 32
3.3 Charts 32
3.3.1 Time Profile Analysis 33
3.3.1.1 Global PK-Analyses 33
3.3.1.2 PK-Analyses 33

4 Sim_HV_3mg/kg 38
4.1 Used building blocks 38
4.2 Simulation Properties 38
4.2.1 Model Structure 38
4.2.2 Compounds 38
4.2.2.1 Bevacizumab_human_sim 38
4.2.3 Administration 39
4.2.3.1 Bevacizumab_human_sim 39
4.3 Charts 39
4.3.1 Time Profile Analysis 40
4.3.1.1 Global PK-Analyses 40
4.3.1.2 PK-Analyses 40

5 Sim_HV_0.5mg/kg 44
5.1 Used building blocks 44
5.2 Simulation Properties 44
5.2.1 Model Structure 44
5.2.2 Compounds 44
5.2.2.1 Bevacizumab_human_sim 44
5.2.3 Administration 45
5.2.3.1 Bevacizumab_human_sim 45
5.3 Charts 45
5.3.1 Time Profile Analysis 46
5.3.1.1 Global PK-Analyses 46
5.3.1.2 PK-Analyses 46

6 Sim_HV_0.5mg/kg_VEGF 50
6.1 Used building blocks 50
6.2 Simulation Properties 50
6.2.1 Model Structure 50
6.2.2 Compounds 50
6.2.2.1 Bevacizumab_human_sim 50
6.2.3 Processes 51
6.2.3.1 Bevacizumab_human_sim 51
6.2.4 Administration 51
6.2.4.1 Bevacizumab_human_sim 51
6.3 Charts 51
6.3.1 Time Profile Analysis 52
6.3.1.1 Global PK-Analyses 52
6.3.1.2 PK-Analyses 52

7 Sim_HV_1mg/kg_VEGF **56**
7.1 Used building blocks 56
7.2 Simulation Properties 56
7.2.1 Model Structure 56
7.2.2 Compounds 56
7.2.2.1 Bevacizumab_human_sim 56
7.2.3 Processes 57
7.2.3.1 Bevacizumab_human_sim 57
7.2.4 Administration 57
7.2.4.1 Bevacizumab_human_sim 57
7.3 Charts 57
7.3.1 Time Profile Analysis 58
7.3.1.1 Global PK-Analyses 58
7.3.1.2 PK-Analyses 58

8 Sim_HV_3mg/kg_VEGF **63**
8.1 Used building blocks 63
8.2 Simulation Properties 63
8.2.1 Model Structure 63
8.2.2 Compounds 63
8.2.2.1 Bevacizumab_human_sim 63
8.2.3 Processes 64
8.2.3.1 Bevacizumab_human_sim 64
8.2.4 Administration 64
8.2.4.1 Bevacizumab_human_sim 64
8.3 Charts 64
8.3.1 Time Profile Analysis 65
8.3.1.1 Global PK-Analyses 65
8.3.1.2 PK-Analyses 65

9 Monkey_beva_4mpk_vegf **69**
9.1 Used building blocks 69
9.2 Simulation Properties 69
9.2.1 Model Structure 69
9.2.2 Compounds 69
9.2.2.1 bevacizumab_monkey_VEGF 69
9.2.3 Processes 70
9.2.3.1 bevacizumab_monkey_VEGF 70
9.2.4 Administration 70
9.2.4.1 bevacizumab_monkey_VEGF 70
9.3 Charts 70
9.3.1 Time Profile Analysis 71
9.3.1.1 Global PK-Analyses 71
9.3.1.2 PK-Analyses 71

III Simulation Comparisons **73**

1 Comparison_Sim_HV_3mg/kg **74**

2 Comparison_Sim_HV_1mg/kg **75**

3 Comparison_Sim_HV_0.5mg/kg **76**

IV Observed Data **77**

1 Beva_Zalevksy2010_monkey_4mpk..Monkey.iv perfusion.4.bevacizumab_monkey **78**

2 Beva-xtend_Zalevksy2010_monkey_4mpk..Monkey.iv perfusion.4.bevacizumab_xtend_monkey **80**

3 Beva_Demarchi2021_HV_1mpk..Human.iv perfusion.1.Bevacizumab_human_sim 82

4 Beva_Hetema2017_HV_1mpk..Human.iv perfusion.1.Bevacizumab_human_sim 83

5 Beva_Hummel2022_HV_1mpk..Human.iv perfusion.1.Bevacizumab_human_sim 84

6 Beva_Wang2019_HV_1mpk..Human.iv perfusion.1.Bevacizumab_human_sim 86

7 Beva_wu2019_HV_1mpk..Human.iv perfusion.1.Bevacizumab_human_sim.1 87

8 Beva_wu2019_HV_1mpk..Human.iv perfusion.1.Bevacizumab_human_sim.2 88

9 Beva_wu2019_HV_1mpk..Human.iv perfusion.1.Bevacizumab_human_sim.3 89

10 Beva_shin2020_HV_3mpk..Human.iv perfusion.3.Bevacizumab_human_sim.1 90

11 Beva_shin2020_HV_3mpk..Human.iv perfusion.3.Bevacizumab_human_sim.2 91

12 Beva_shin2020_HV_3mpk..Human.iv perfusion.3.Bevacizumab_human_sim.3 92

13 Beva_sinn2021_HV_3mpk..Human.iv perfusion.3.Bevacizumab_human_sim 93

14 Beva_li2017_HV_0.5mpk..Human.iv perfusion.0.5.Bevacizumab_human_sim.1 95

15 Beva_li2017_HV_0.5mpk..Human.iv perfusion.0.5.Bevacizumab_human_sim.2 96

16 Beva_li2017_HV_0.5mpk..Human.iv perfusion.0.5.Bevacizumab_human_sim.3 97

17 Beva_li2017_HV_0.5mpk..Human.iv perfusion.0.5.Bevacizumab_human_sim.4 98

18 Beva_li2017_HV_0.5mpk..Human.iv perfusion.0.5.Bevacizumab_human_sim.5 99

Part I

Building Blocks

Chapter 1

Individuals

1.1 monkey

1.1.1 Biometrics

Population Properties

Population Properties	Value
Species	Monkey
Population	Monkey
Gender	Unknown

Table 1.1: Population Properties

Individual Parameters

Individual Parameters	Value	Unit
Weight	3.00	kg

Table 1.2: Individual Parameters

Calculation methods

Category	Calculation methods
Endothelial surface areas	Organ vascularization

Table 1.3: Calculation methods

1.1.2 Anatomy & Physiology

Default

1.1.3 Expression

1.1.3.1 Metabolizing Enzymes

None

1.1.3.2 Transport Proteins

None

1.1.3.3 Protein Binding Partners

VEGFA
Reference concentration: 3.00E-4 µmol/l
t1/2 (liver): 36.00 h
t1/2 (intestine): 23.00 h
Ontogeny/Variability like: Undefined
Localization: Interstitial, BloodCellsMembrane, VascMembraneTissueSide
Expression Levels

Relative expression	Name	Value
VEGFA	0.18	0.18 %
VEGFA	0.18	0.18 %

Table 1.4: Expression Levels

1.2 HV_sim

1.2.1 Biometrics

Population Properties

Population Properties	Value
Species	Human
Population	European (ICRP, 2002)
Gender	Male

Table 1.5: Population Properties

Individual Parameters

Individual Parameters	Value	Unit
Age	30.00	year(s)
Weight	73.00	kg
Height	176.00	cm
BMI	23.57	kg/m^2

Table 1.6: Individual Parameters

Calculation methods

Category	Calculation methods
Endothelial surface areas	Organ vascularization
Body surface area	Mosteller

Table 1.7: Calculation methods

1.2.2 Anatomy & Physiology

Default

1.2.3 Expression

1.2.3.1 Metabolizing Enzymes

None

1.2.3.2 Transport Proteins

None

1.2.3.3 Protein Binding Partners

None

1.3 HV_sim_VEGF

1.3.1 Biometrics

Population Properties

Population Properties	Value
Species	Human
Population	European (ICRP, 2002)
Gender	Male

Table 1.8: Population Properties

Individual Parameters

Individual Parameters	Value	Unit
Age	30.00	year(s)
Weight	73.00	kg
Height	176.00	cm
BMI	23.57	kg/m^2

Table 1.9: Individual Parameters

Calculation methods

Category	Calculation methods
Endothelial surface areas	Organ vascularization
Body surface area	Mosteller

Table 1.10: Calculation methods

1.3.2 Anatomy & Physiology

Default

1.3.3 Expression

1.3.3.1 Metabolizing Enzymes

None

1.3.3.2 Transport Proteins

None

1.3.3.3 Protein Binding Partners

VEGFA
Reference concentration: 3.00E-3 µmol/l
t1/2 (liver): 36.00 h
t1/2 (intestine): 23.00 h
Ontogeny/Variability like: Undefined
Localization: Interstitial, BloodCellsMembrane, VascMembraneTissueSide
Expression Levels

Relative expression	Name	Value
VEGFA	0.18	0.18 %
VEGFA	0.18	0.18 %

Table 1.11: Expression Levels

1.4 monkey_VEGF

1.4.1 Biometrics

Population Properties

Population Properties	Value
Species	Monkey
Population	Monkey
Gender	Unknown

Table 1.12: Population Properties

Individual Parameters

Individual Parameters	Value	Unit
Weight	3.00	kg

Table 1.13: Individual Parameters

Calculation methods

Category	Calculation methods
Endothelial surface areas	Organ vascularization

Table 1.14: Calculation methods

1.4.2 Anatomy & Physiology

Default

1.4.3 Expression

1.4.3.1 Metabolizing Enzymes

None

1.4.3.2 Transport Proteins

None

1.4.3.3 Protein Binding Partners

VEGFA
Reference concentration: 3.00E-3 µmol/l
t1/2 (liver): 36.00 h
t1/2 (intestine): 23.00 h
Ontogeny/Variability like: Undefined
Localization: Interstitial, BloodCellsMembrane, VascMembraneTissueSide
Expression Levels

Relative expression	Name	Value
VEGFA	0.18	0.18 %
VEGFA	0.18	0.18 %

Table 1.15: Expression Levels

Chapter 2

Compounds

2.1 bevacizumab_monkey

2.1.1 Basic Physico-chemistry

Is small molecule: No

2.1.1.1 Molecular weight

Parameter	Value	Unit
Molecular weight	150.00	kDa
Effective molecular weight	150000.00	g/mol
I	0	
F	0	
Cl	0	
Br	0	

Table 2.1: Molecular weight

2.1.1.2 Lipophilicity

Experiment	Lipophilicity [Log Units]
Measurement	−5.00

Table 2.2: Lipophilicity

Table 2.2 lists lipophilicity values for compound bevacizumab_monkey.

2.1.1.3 Fraction unbound (plasma, reference value)

Experiment	Fraction Unbound	Species
Measurement	1.00	Monkey

Table 2.3: Fraction unbound (plasma, reference value)

Table 2.3 lists fraction unbound values for compound bevacizumab_monkey.

2.1.1.4 Solubility

Experiment	Ref-pH	Solubility at Ref-pH [mg/l]	Solubility gain per charge
Measurement	7.00	9999.00	1000.00

Table 2.4: Solubility

Table 2.4 lists solubility values for compound bevacizumab_monkey.

2.1.2 ADME

2.1.2.1 Absorption

Specific intestinal permeability

Experiment	Lipophilicity	Permeability [cm/min]
Calculated	Measurement	0

Table 2.5: Specific intestinal permeability

Table 2.5 lists intestinal permeability values for compound bevacizumab_monkey.

2.1.2.2 Distribution

Specific organ permeability

Experiment	Lipophilicity	Permeability [cm/min]
Calculated	Measurement	0

Table 2.6: Specific organ permeability

Table 2.6 lists organ permeability values for compound bevacizumab_monkey.

Calculation methods

Category	Calculation methods
Partition coefficients	PK-Sim Standard
Cellular permeabilities	PK-Sim Standard

Table 2.7: Calculation methods

2.2 bevacizumab_xtend_monkey

2.2.1 Basic Physico-chemistry

Is small molecule: No

2.2.1.1 Molecular weight

Parameter	Value	Unit
Molecular weight	150.00	kDa
Effective molecular weight	150000.00	g/mol
I	0	
F	0	
Cl	0	
Br	0	

Table 2.8: Molecular weight

2.2.1.2 Lipophilicity

Experiment	Lipophilicity [Log Units]
Measurement	−5.00

Table 2.9: Lipophilicity

Table 2.9 lists lipophilicity values for compound bevacizumab_xtend_monkey.

2.2.1.3 Fraction unbound (plasma, reference value)

Experiment	Fraction Unbound	Species
Measurement	1.00	Monkey

Table 2.10: Fraction unbound (plasma, reference value)

Table 2.10 lists fraction unbound values for compound bevacizumab_xtend_monkey.

2.2.1.4 Solubility

Experiment	Ref-pH	Solubility at Ref-pH [mg/l]	Solubility gain per charge
Measurement	7.00	9999.00	1000.00

Table 2.11: Solubility

Table 2.11 lists solubility values for compound bevacizumab_xtend_monkey.

2.2.2 ADME

2.2.2.1 Absorption

Specific intestinal permeability

Experiment	Lipophilicity	Permeability [cm/min]
Calculated	Measurement	0

Table 2.12: Specific intestinal permeability

Table 2.12 lists intestinal permeability values for compound bevacizumab_xtend_monkey.

2.2.2.2 Distribution

Specific organ permeability

Experiment	Lipophilicity	Permeability [cm/min]
Calculated	Measurement	0

Table 2.13: Specific organ permeability

Table 2.13 lists organ permeability values for compound bevacizumab_xtend_monkey.

Calculation methods

Category	Calculation methods
Partition coefficients	PK-Sim Standard
Cellular permeabilities	PK-Sim Standard

Table 2.14: Calculation methods

2.3 Bevacizumab_human_sim

2.3.1 Basic Physico-chemistry

Is small molecule: No

2.3.1.1 Molecular weight

Parameter	Value	Unit
Molecular weight	150.00	kDa
Effective molecular weight	150000.00	g/mol
I	0	
F	0	
Cl	0	
Br	0	

Table 2.15: Molecular weight

2.3.1.2 Lipophilicity

Experiment	Lipophilicity [Log Units]
Measurement	−5.00

Table 2.16: Lipophilicity

Table 2.16 lists lipophilicity values for compound Bevacizumab_human_sim.

2.3.1.3 Fraction unbound (plasma, reference value)

Experiment	Fraction Unbound	Species
Measurement	1.00	Human

Table 2.17: Fraction unbound (plasma, reference value)

Table 2.17 lists fraction unbound values for compound Bevacizumab_human_sim.

2.3.1.4 Solubility

Experiment	Ref-pH	Solubility at Ref-pH [mg/l]	Solubility gain per charge
Measurement	7.00	9999.00	1000.00

Table 2.18: Solubility

Table 2.18 lists solubility values for compound Bevacizumab_human_sim.

2.3.2 ADME

2.3.2.1 Absorption

Specific intestinal permeability

Experiment	Lipophilicity	Permeability [cm/min]
Calculated	Measurement	0

Table 2.19: Specific intestinal permeability

Table 2.19 lists intestinal permeability values for compound Bevacizumab_human_sim.

2.3.2.2 Distribution

Specific organ permeability

Experiment	Lipophilicity	Permeability [cm/min]
Calculated	Measurement	0

Table 2.20: Specific organ permeability

Table 2.20 lists organ permeability values for compound Bevacizumab_human_sim.

Calculation methods

Category	Calculation methods
Partition coefficients	PK-Sim Standard
Cellular permeabilities	PK-Sim Standard

Table 2.21: Calculation methods

VEGF 2-Paper 2
Process Type: Specific Binding

Parameter	Value	Unit
koff	3.10×10^{-5}	1/s
Kd	0.06	nmol/l

Table 2.22: Parameters

VEGFA-Paper
Process Type: Specific Binding

Parameter	Value	Unit
koff	3.10×10^{-5}	1/s
Kd	0.06	nmol/l

Table 2.23: Parameters

2.4 bevacizumab_monkey_VEGF

2.4.1 Basic Physico-chemistry

Is small molecule: No

2.4.1.1 Molecular weight

Parameter	Value	Unit
Molecular weight	150.00	kDa
Effective molecular weight	150000.00	g/mol
I	0	
F	0	
Cl	0	
Br	0	

Table 2.24: Molecular weight

2.4.1.2 Lipophilicity

Experiment	Lipophilicity [Log Units]
Measurement	−5.00

Table 2.25: Lipophilicity

Table 2.25 lists lipophilicity values for compound bevacizumab_monkey_VEGF.

2.4.1.3 Fraction unbound (plasma, reference value)

Experiment	Fraction Unbound	Species
Measurement	1.00	Monkey

Table 2.26: Fraction unbound (plasma, reference value)

Table 2.26 lists fraction unbound values for compound bevacizumab_monkey_VEGF.

2.4.1.4 Solubility

Experiment	Ref-pH	Solubility at Ref-pH [mg/l]	Solubility gain per charge
Measurement	7.00	9999.00	1000.00

Table 2.27: Solubility

Table 2.27 lists solubility values for compound bevacizumab_monkey_VEGF.

2.4.2 ADME

2.4.2.1 Absorption

Specific intestinal permeability

Experiment	Lipophilicity	Permeability [cm/min]
Calculated	Measurement	0

Table 2.28: Specific intestinal permeability

Table 2.28 lists intestinal permeability values for compound bevacizumab_monkey_VEGF.

2.4.2.2 Distribution

Specific organ permeability

Experiment	Lipophilicity	Permeability [cm/min]
Calculated	Measurement	0

Table 2.29: Specific organ permeability

Table 2.29 lists organ permeability values for compound bevacizumab_monkey_VEGF.

Calculation methods

Category	Calculation methods
Partition coefficients	PK-Sim Standard
Cellular permeabilities	PK-Sim Standard

Table 2.30: Calculation methods

VEGFA-paper
Process Type: Specific Binding

Parameter	Value	Unit
koff	4.06	1/s
Kd	0.03	nmol/l

Table 2.31: Parameters

Chapter 3

Protocols

3.1 Perfusion 60 min_4 mg/kg

Property	Value
Process Type	Simple protocol
Administration type	Intravenous Infusion
Dosing interval	Single

Table 3.1

Parameter	Value	Unit
Dose	4.00	mg/kg
Infusion time	60.00	min

Table 3.2: Parameters

3.2 Perfusion 90 min_1mg/kg

Property	Value
Process Type	Simple protocol
Administration type	Intravenous Infusion
Dosing interval	Single

Table 3.3

Parameter	Value	Unit
Dose	1.00	mg/kg
Infusion time	90.00	min

Table 3.4: Parameters

3.3 Perfusion 90 min_3mg/kg

Property	Value
Process Type	Simple protocol
Administration type	Intravenous Infusion
Dosing interval	Single

Table 3.5

Parameter	Value	Unit
Dose	3.00	mg/kg
Infusion time	90.00	min

Table 3.6: Parameters

3.4 Perfusion 90 min_0.5mg/kg

Property	Value
Process Type	Simple protocol
Administration type	Intravenous Infusion
Dosing interval	Single

Table 3.7

Parameter	Value	Unit
Dose	0.50	mg/kg
Infusion time	90.00	min

Table 3.8: Parameters

Part II

Simulations

Chapter 1

Monkey_beva_4mpk

1.1 Used building blocks

Building Block	Name
Individual	monkey (see section 1.1 in Part I)
Compound	bevacizumab_monkey (see section 2.1 in Part I)
Protocol	Perfusion 60 min_4 mg/kg (see section 3.1 in Part I)

Table 1.1: Building Block

1.2 Simulation Properties

1.2.1 Model Structure

Allow aging
No

Calculation methods

Category	Calculation methods
Endothelial surface areas	Organ vascularization

Table 1.2: Calculation methods

1.2.2 Compounds

1.2.2.1 bevacizumab_monkey

Compound Configuration

Parameter	Alternative in compound	Value	Unit
Solubility	Measurement	9999.00	mg/l
Lipophilicity	Measurement	−5.00	Log Units
Fraction unbound (plasma, reference value)	Measurement	1.00	
Specific organ permeability	Calculated	0	cm/min

continued on next page

continued from previous page			
**Parameter**	**Alternative in compound**	**Value**	**Unit**
Specific intestinal permeability	Calculated	0	cm/min

Table 1.3: Compound Configuration

Calculation methods

Category	Calculation methods
Partition coefficients	PK-Sim Standard
Cellular permeabilities	PK-Sim Standard

Table 1.4: Calculation methods

1.2.3 Administration

1.2.3.1 bevacizumab_monkey

Simple protocol
Intravenous Infusion
Dosing interval: Single
Dose: 4.00 mg/kg
Infusion time: 60.00 min

1.3 Charts

1.3.1 Time Profile Analysis

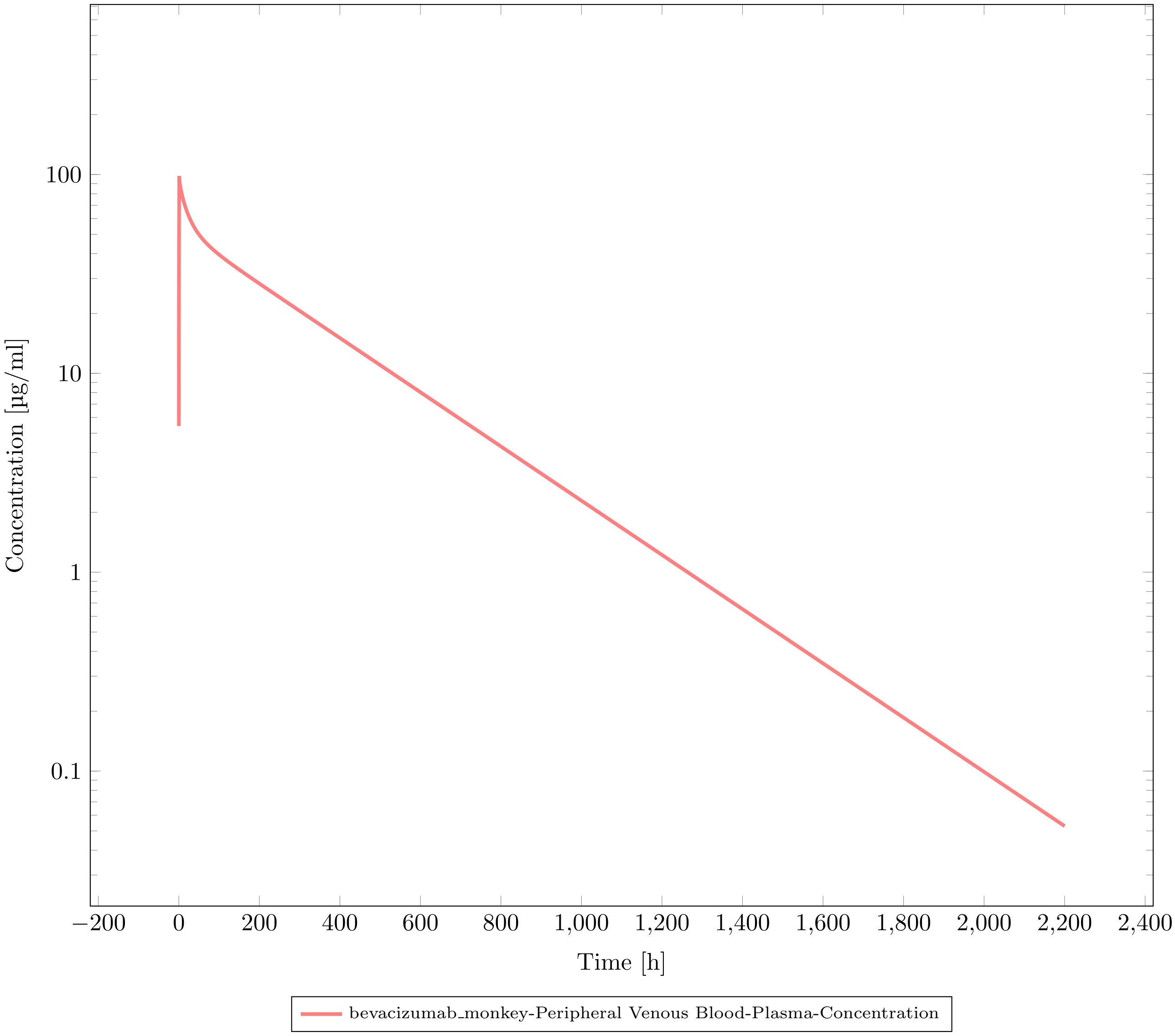

Figure 1.1

1.3.1.1 Global PK-Analyses

Parameter	Compound	Value	Unit
Vss (plasma)	bevacizumab_monkey	68.43	ml/kg
Vd (plasma)	bevacizumab_monkey	71.82	ml/kg
Vss (phys-chem)	bevacizumab_monkey	644.98	ml/kg
Total plasma clearance	bevacizumab_monkey	3.76×10^{-3}	ml/min/kg

Table 1.5: Global PK-Analyses

1.3.1.2 PK-Analyses

bevacizumab_monkey-Peripheral Venous Blood-Plasma-Concentration

Parameter	Value	Unit
C_max	0.65	µmol/l
C_max_norm	2.45×10^{7}	mg/l
t_max	1.00	h
C_tEnd	3.52×10^{-4}	µmol/l
AUC_tEnd	7086.18	µmol*min/l
AUC_tEnd_norm	2.66×10^{14}	µg*min/l
AUC_inf	7092.91	µmol*min/l
AUC_inf_norm	2.66×10^{14}	µg*min/l
MRT	303.35	h
Half-Life	220.69	h
% AUC (tlast-∞)	9.49×10^{-4}	
Total body clearance/F	3.76×10^{-3}	ml/min/kg
Vss (plasma)/F	68.43	ml/kg
Vd (plasma)/F	71.82	ml/kg

Table 1.6: PK-Analyses for bevacizumab_monkey-Peripheral Venous Blood-Plasma-Concentration

Beva_Zalevksy2010_monkey_4mpk..Monkey.iv perfusion.4.bevacizumab_monkey-bevacizumab_monkey-Measurement

Parameter	Value	Unit
C_max	0.77	µmol/l
C_max_norm	2.90×10^{7}	mg/l
t_max	1.92	h
C_tEnd	6.74×10^{-4}	µmol/l
AUC_tEnd	7847.21	µmol*min/l
AUC_tEnd_norm	2.94×10^{14}	µg*min/l
AUC_inf	7863.87	µmol*min/l
AUC_inf_norm	2.95×10^{14}	µg*min/l
MRT	339.82	h
Half-Life	285.61	h
% AUC (tlast-∞)	2.12×10^{-3}	
Total body clearance/F	3.39×10^{-3}	ml/min/kg
Vss (plasma)/F	69.14	ml/kg
Vd (plasma)/F	83.84	ml/kg

Table 1.7: PK-Analyses for Beva_Zalevksy2010_monkey_4mpk..Monkey.iv perfusion.4.bevacizumab_monkey-bevacizumab_monkey-Measurement

Chapter 2

Monkey_beva-xtend_4mpk

2.1 Used building blocks

Building Block	Name
Individual	monkey (see section 1.1 in Part I)
Compound	bevacizumab_xtend_monkey (see section 2.2 in Part I)
Protocol	Perfusion 60 min_4 mg/kg (see section 3.1 in Part I)

Table 2.1: Building Block

2.2 Simulation Properties

2.2.1 Model Structure

Allow aging
No

Calculation methods

Category	Calculation methods
Endothelial surface areas	Organ vascularization

Table 2.2: Calculation methods

2.2.2 Compounds

2.2.2.1 bevacizumab_xtend_monkey

Compound Configuration

Parameter	Alternative in compound	Value	Unit
Solubility	Measurement	9999.00	mg/l
Lipophilicity	Measurement	−5.00	Log Units
Fraction unbound (plasma, reference value)	Measurement	1.00	
Specific organ permeability	Calculated	0	cm/min
			continued on next page

continued from previous page			
**Parameter**	**Alternative in compound**	**Value**	**Unit**
Specific intestinal permeability	Calculated	0	cm/min

Table 2.3: Compound Configuration

Calculation methods

Category	Calculation methods
Partition coefficients	PK-Sim Standard
Cellular permeabilities	PK-Sim Standard

Table 2.4: Calculation methods

2.2.3 Administration

2.2.3.1 bevacizumab_xtend_monkey

Simple protocol
Intravenous Infusion
Dosing interval: Single
Dose: 4.00 mg/kg
Infusion time: 60.00 min

2.3 Charts

2.3.1 Time Profile Analysis

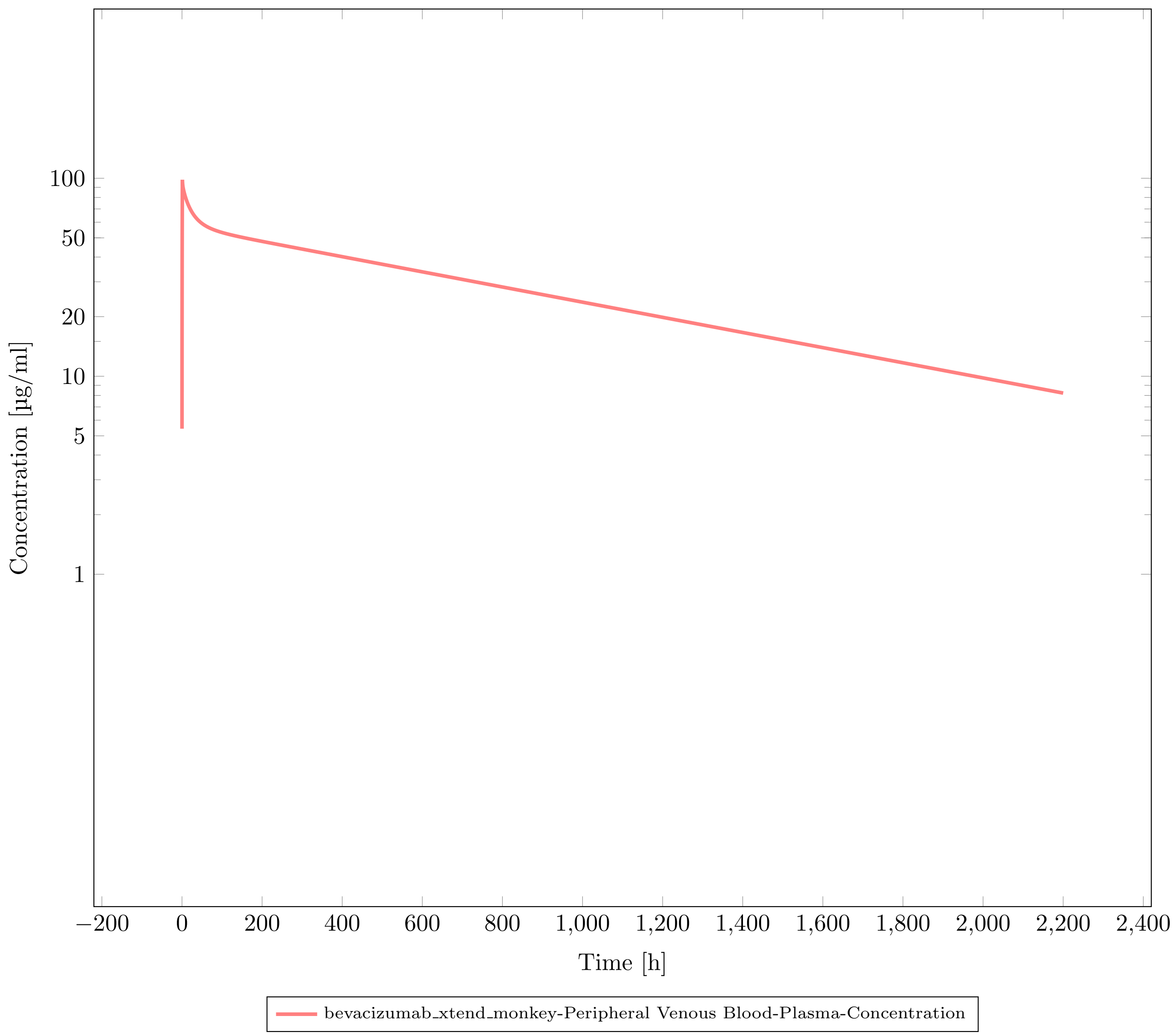

Figure 2.1

2.3.1.1 Global PK-Analyses

Parameter	Compound	Value	Unit
Vss (plasma)	bevacizumab_xtend_monkey	68.25	ml/kg
Vd (plasma)	bevacizumab_xtend_monkey	69.13	ml/kg
Vss (phys-chem)	bevacizumab_xtend_monkey	644.98	ml/kg
Total plasma clearance	bevacizumab_xtend_monkey	1.02×10^{-3}	ml/min/kg

Table 2.5: Global PK-Analyses

2.3.1.2 PK-Analyses

bevacizumab_xtend_monkey-Peripheral Venous Blood-Plasma-Concentration

Parameter	Value	Unit
C_max	0.66	µmol/l
C_max_norm	2.46×10^{7}	mg/l
t_max	1.00	h
C_tEnd	0.05	µmol/l
AUC_tEnd	22535.66	µmol*min/l
AUC_tEnd_norm	8.45×10^{14}	µg*min/l
AUC_inf	26269.32	µmol*min/l
AUC_inf_norm	9.85×10^{14}	µg*min/l
MRT	1120.59	h
Half-Life	786.73	h
% AUC (tlast-∞)	0.14	
Total body clearance/F	1.02×10^{-3}	ml/min/kg
Vss (plasma)/F	68.25	ml/kg
Vd (plasma)/F	69.13	ml/kg

Table 2.6: PK-Analyses for bevacizumab_xtend_monkey-Peripheral Venous Blood-Plasma-Concentration

Beva-xtend_Zalevksy2010_monkey_4mpk..Monkey.iv perfusion.4.bevacizumab_xtend_monkey-bevacizumab_-xtend_monkey-Measurement

Parameter	Value	Unit
C_max	0.81	µmol/l
C_max_norm	3.05×10^{7}	mg/l
t_max	1.93	h
C_tEnd	0.05	µmol/l
AUC_tEnd	21298.20	µmol*min/l
AUC_tEnd_norm	7.99×10^{14}	µg*min/l
AUC_inf	24317.36	µmol*min/l
AUC_inf_norm	9.12×10^{14}	µg*min/l
MRT	1020.02	h
Half-Life	668.24	h
% AUC (tlast-∞)	0.12	
Total body clearance/F	1.10×10^{-3}	ml/min/kg
Vss (plasma)/F	67.11	ml/kg
Vd (plasma)/F	63.43	ml/kg

Table 2.7: PK-Analyses for Beva-xtend_Zalevksy2010_monkey_4mpk..Monkey.iv perfusion.4.bevacizumab_-xtend_monkey-bevacizumab_xtend_monkey-Measurement

Chapter 3

Sim_HV_1mg/kg

3.1 Used building blocks

Building Block	Name
Individual	HV_sim (see section 1.2 in Part I)
Compound	Bevacizumab_human_sim (see section 2.3 in Part I)
Protocol	Perfusion 90 min_1mg/kg (see section 3.2 in Part I)

Table 3.1: Building Block

3.2 Simulation Properties

3.2.1 Model Structure

Allow aging
No

Calculation methods

Category	Calculation methods
Endothelial surface areas	Organ vascularization
Body surface area	Mosteller

Table 3.2: Calculation methods

3.2.2 Compounds

3.2.2.1 Bevacizumab_human_sim

Compound Configuration

Parameter	Alternative in compound	Value	Unit
Solubility	Measurement	9999.00	mg/l
Lipophilicity	Measurement	−5.00	Log Units
Fraction unbound (plasma, reference value)	Measurement	1.00	
continued on next page			

continued from previous page			
**Parameter**	**Alternative in compound**	**Value**	**Unit**
Specific organ permeability	Calculated	0	cm/min
Specific intestinal permeability	Calculated	0	cm/min

Table 3.3: Compound Configuration

Calculation methods

Category	Calculation methods
Partition coefficients	PK-Sim Standard
Cellular permeabilities	PK-Sim Standard

Table 3.4: Calculation methods

3.2.3 Administration

3.2.3.1 Bevacizumab_human_sim

Simple protocol
Intravenous Infusion
Dosing interval: Single
Dose: 1.00 mg/kg
Infusion time: 90.00 min

3.3 Charts

3.3.1 Time Profile Analysis

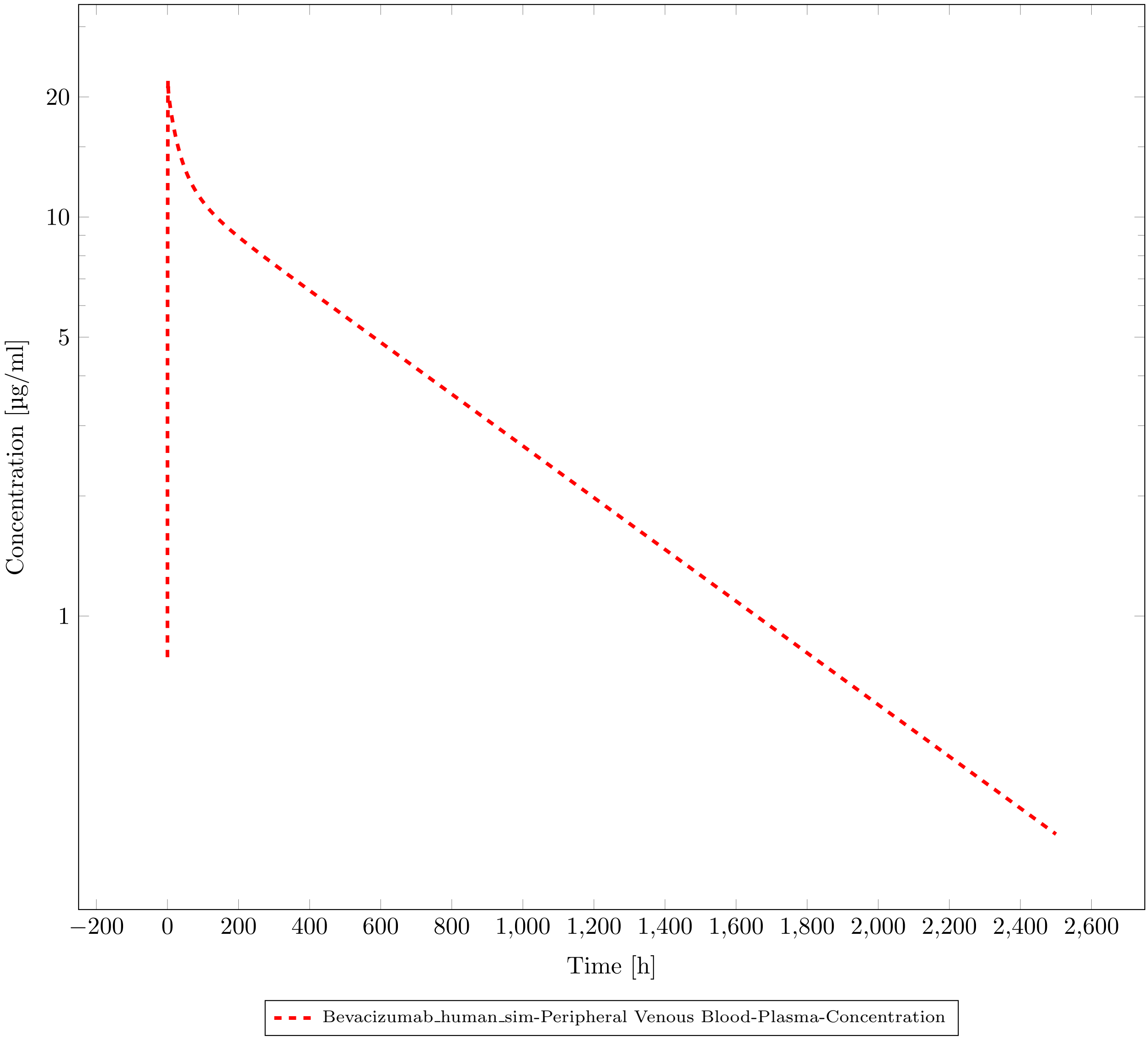

Figure 3.1

3.3.1.1 Global PK-Analyses

Parameter	Compound	Value	Unit
Vss (plasma)	Bevacizumab_human_sim	77.87	ml/kg
Vd (plasma)	Bevacizumab_human_sim	80.88	ml/kg
Vss (phys-chem)	Bevacizumab_human_sim	623.05	ml/kg
Total plasma clearance	Bevacizumab_human_sim	2.01×10^{-3}	ml/min/kg

Table 3.5: Global PK-Analyses

3.3.1.2 PK-Analyses

Bevacizumab_human_sim-Peripheral Venous Blood-Plasma-Concentration

Parameter	Value	Unit
C_max	0.15	µmol/l
C_max_norm	2.21×10^{7}	mg/l
t_max	1.50	h
C_tEnd	1.89×10^{-3}	µmol/l
AUC_tEnd	3235.50	µmol*min/l
AUC_tEnd_norm	4.85×10^{14}	µg*min/l
AUC_inf	3311.59	µmol*min/l
AUC_inf_norm	4.97×10^{14}	µg*min/l
MRT	644.69	h
Half-Life	464.14	h
% AUC (tlast-∞)	0.02	
Total body clearance/F	2.01×10^{-3}	ml/min/kg
Vss (plasma)/F	77.87	ml/kg
Vd (plasma)/F	80.88	ml/kg

Table 3.6: PK-Analyses for Bevacizumab_human_sim-Peripheral Venous Blood-Plasma-Concentration

Beva_Demarchi2021_HV_1mpk..Human.iv perfusion.1.Bevacizumab_human_sim-Bevacizumab_human_-sim-Measurement

Parameter	Value	Unit
C_max	0.15	µmol/l
C_max_norm	2.30×10^{7}	mg/l
t_max	4.86	h
C_tEnd	7.71×10^{-5}	µmol/l
AUC_tEnd	2942.18	µmol*min/l
AUC_tEnd_norm	4.41×10^{14}	µg*min/l
AUC_inf	2942.77	µmol*min/l
AUC_inf_norm	4.41×10^{14}	µg*min/l
MRT	448.08	h
Half-Life	89.57	h
% AUC (tlast-∞)	2.03×10^{-4}	
Total body clearance/F	2.27×10^{-3}	ml/min/kg
Vss (plasma)/F	60.91	ml/kg
Vd (plasma)/F	17.56	ml/kg

Table 3.7: PK-Analyses for Beva_Demarchi2021_HV_1mpk..Human.iv perfusion.1.Bevacizumab_human_sim-Bevacizumab_human_sim-Measurement

Beva_Hetema2017_HV_1mpk..Human.iv perfusion.1.Bevacizumab_human_sim-Bevacizumab_human_-sim-Measurement

Parameter	Value	Unit
C_max	0.16	µmol/l
C_max_norm	2.45×10^{7}	mg/l
t_max	0.64	h
C_tEnd	8.38×10^{-3}	µmol/l
AUC_tEnd	2638.13	µmol*min/l
AUC_tEnd_norm	3.96×10^{14}	µg*min/l
AUC_inf	NaN	µmol*min/l
AUC_inf_norm	NaN	µg*min/l
MRT	NaN	h
Half-Life	Infinity	h
% AUC (tlast-∞)	NaN	
Total body clearance/F	NaN	ml/min/kg
Vss (plasma)/F	NaN	ml/kg
Vd (plasma)/F	NaN	ml/kg

Table 3.8: PK-Analyses for Beva_Hetema2017_HV_1mpk..Human.iv perfusion.1.Bevacizumab_human_sim-Bevacizumab_human_sim-Measurement

Beva_Hummel2022_HV_1mpk..Human.iv perfusion.1.Bevacizumab_human_sim-Bevacizumab_human_-sim-Measurement

Parameter	Value	Unit
C_max	0.17	µmol/l
C_max_norm	2.50×10^{7}	mg/l
t_max	1.50	h
C_tEnd	6.47×10^{-4}	µmol/l
AUC_tEnd	3230.16	µmol*min/l
AUC_tEnd_norm	4.85×10^{14}	µg*min/l
AUC_inf	3241.46	µmol*min/l
AUC_inf_norm	4.86×10^{14}	µg*min/l
MRT	476.41	h
Half-Life	201.77	h
% AUC (tlast-∞)	3.49×10^{-3}	
Total body clearance/F	2.06×10^{-3}	ml/min/kg
Vss (plasma)/F	58.79	ml/kg
Vd (plasma)/F	35.92	ml/kg

Table 3.9: PK-Analyses for Beva_Hummel2022_HV_1mpk..Human.iv perfusion.1.Bevacizumab_human_sim-Bevacizumab_human_sim-Measurement

Beva_Wang2019_HV_1mpk..Human.iv perfusion.1.Bevacizumab_human_sim-Bevacizumab_human_-sim-Measurement

Parameter	Value	Unit
C_max	0.11	µmol/l

continued on next page

continued from previous page		
**Parameter**	**Value**	**Unit**
C_max_norm	1.70×10^{7}	mg/l
t_max	1.50	h
C_tEnd	1.38×10^{-3}	µmol/l
AUC_tEnd	2209.27	µmol*min/l
AUC_tEnd_norm	3.31×10^{14}	µg*min/l
AUC_inf	6061.43	µmol*min/l
AUC_inf_norm	9.09×10^{14}	µg*min/l
MRT	31340.36	h
Half-Life	32359.70	h
% AUC (tlast-∞)	0.64	
Total body clearance/F	1.10×10^{-3}	ml/min/kg
Vss (plasma)/F	2068.18	ml/kg
Vd (plasma)/F	3080.80	ml/kg

Table 3.10: PK-Analyses for Beva_Wang2019_HV_1mpk..Human.iv perfusion.1.Bevacizumab_human_sim-Bevacizumab_human_sim-Measurement

Beva_wu2019_HV_1mpk..Human.iv perfusion.1.Bevacizumab_human_sim.1-Bevacizumab_human_-sim-Measurement

Parameter	Value	Unit
C_max	0.17	µmol/l
C_max_norm	2.50×10^{7}	mg/l
t_max	1.50	h
C_tEnd	1.18×10^{-3}	µmol/l
AUC_tEnd	2779.89	µmol*min/l
AUC_tEnd_norm	4.17×10^{14}	µg*min/l
AUC_inf	2821.02	µmol*min/l
AUC_inf_norm	4.23×10^{14}	µg*min/l
MRT	532.48	h
Half-Life	404.25	h
% AUC (tlast-∞)	0.01	
Total body clearance/F	2.36×10^{-3}	ml/min/kg
Vss (plasma)/F	75.50	ml/kg
Vd (plasma)/F	82.70	ml/kg

Table 3.11: PK-Analyses for Beva_wu2019_HV_1mpk..Human.iv perfusion.1.Bevacizumab_human_sim.1-Bevacizumab_human_sim-Measurement

Beva_wu2019_HV_1mpk..Human.iv perfusion.1.Bevacizumab_human_sim.2-Bevacizumab_human_-sim-Measurement

Parameter	Value	Unit
C_max	0.12	µmol/l
		continued on next page

continued from previous page		
**Parameter**	**Value**	**Unit**
C_max_norm	1.81×10^{7}	mg/l
t_max	14.58	h
C_tEnd	2.20×10^{-4}	µmol/l
AUC_tEnd	2257.21	µmol*min/l
AUC_tEnd_norm	3.39×10^{14}	µg*min/l
AUC_inf	NaN	µmol*min/l
AUC_inf_norm	NaN	µg*min/l
MRT	NaN	h
Half-Life	Infinity	h
% AUC (tlast-∞)	NaN	
Total body clearance/F	NaN	ml/min/kg
Vss (plasma)/F	NaN	ml/kg
Vd (plasma)/F	NaN	ml/kg

Table 3.12: PK-Analyses for Beva_wu2019_HV_1mpk..Human.iv perfusion.1.Bevacizumab_human_sim.2-Bevacizumab_human_sim-Measurement

Beva_wu2019_HV_1mpk..Human.iv perfusion.1.Bevacizumab_human_sim.3-Bevacizumab_human_-sim-Measurement

Parameter	Value	Unit
C_max	0.12	µmol/l
C_max_norm	1.81×10^{7}	mg/l
t_max	14.58	h
C_tEnd	2.48×10^{-3}	µmol/l
AUC_tEnd	2995.12	µmol*min/l
AUC_tEnd_norm	4.49×10^{14}	µg*min/l
AUC_inf	NaN	µmol*min/l
AUC_inf_norm	NaN	µg*min/l
MRT	NaN	h
Half-Life	Infinity	h
% AUC (tlast-∞)	NaN	
Total body clearance/F	NaN	ml/min/kg
Vss (plasma)/F	NaN	ml/kg
Vd (plasma)/F	NaN	ml/kg

Table 3.13: PK-Analyses for Beva_wu2019_HV_1mpk..Human.iv perfusion.1.Bevacizumab_human_sim.3-Bevacizumab_human_sim-Measurement

Chapter 4

Sim_HV_3mg/kg

4.1 Used building blocks

Building Block	Name
Individual	HV_sim (see section 1.2 in Part I)
Compound	Bevacizumab_human_sim (see section 2.3 in Part I)
Protocol	Perfusion 90 min_3mg/kg (see section 3.3 in Part I)

Table 4.1: Building Block

4.2 Simulation Properties

4.2.1 Model Structure

Allow aging
No

Calculation methods

Category	Calculation methods
Endothelial surface areas	Organ vascularization
Body surface area	Mosteller

Table 4.2: Calculation methods

4.2.2 Compounds

4.2.2.1 Bevacizumab_human_sim

Compound Configuration

Parameter	Alternative in compound	Value	Unit
Solubility	Measurement	9999.00	mg/l
Lipophilicity	Measurement	−5.00	Log Units
Fraction unbound (plasma, reference value)	Measurement	1.00	
continued on next page			

continued from previous page			
**Parameter**	**Alternative in compound**	**Value**	**Unit**
Specific organ permeability	Calculated	0	cm/min
Specific intestinal permeability	Calculated	0	cm/min

Table 4.3: Compound Configuration

Calculation methods

Category	Calculation methods
Partition coefficients	PK-Sim Standard
Cellular permeabilities	PK-Sim Standard

Table 4.4: Calculation methods

4.2.3 Administration

4.2.3.1 Bevacizumab_human_sim

Simple protocol
Intravenous Infusion
Dosing interval: Single
Dose: 3.00 mg/kg
Infusion time: 90.00 min

4.3 Charts

4.3.1 Time Profile Analysis

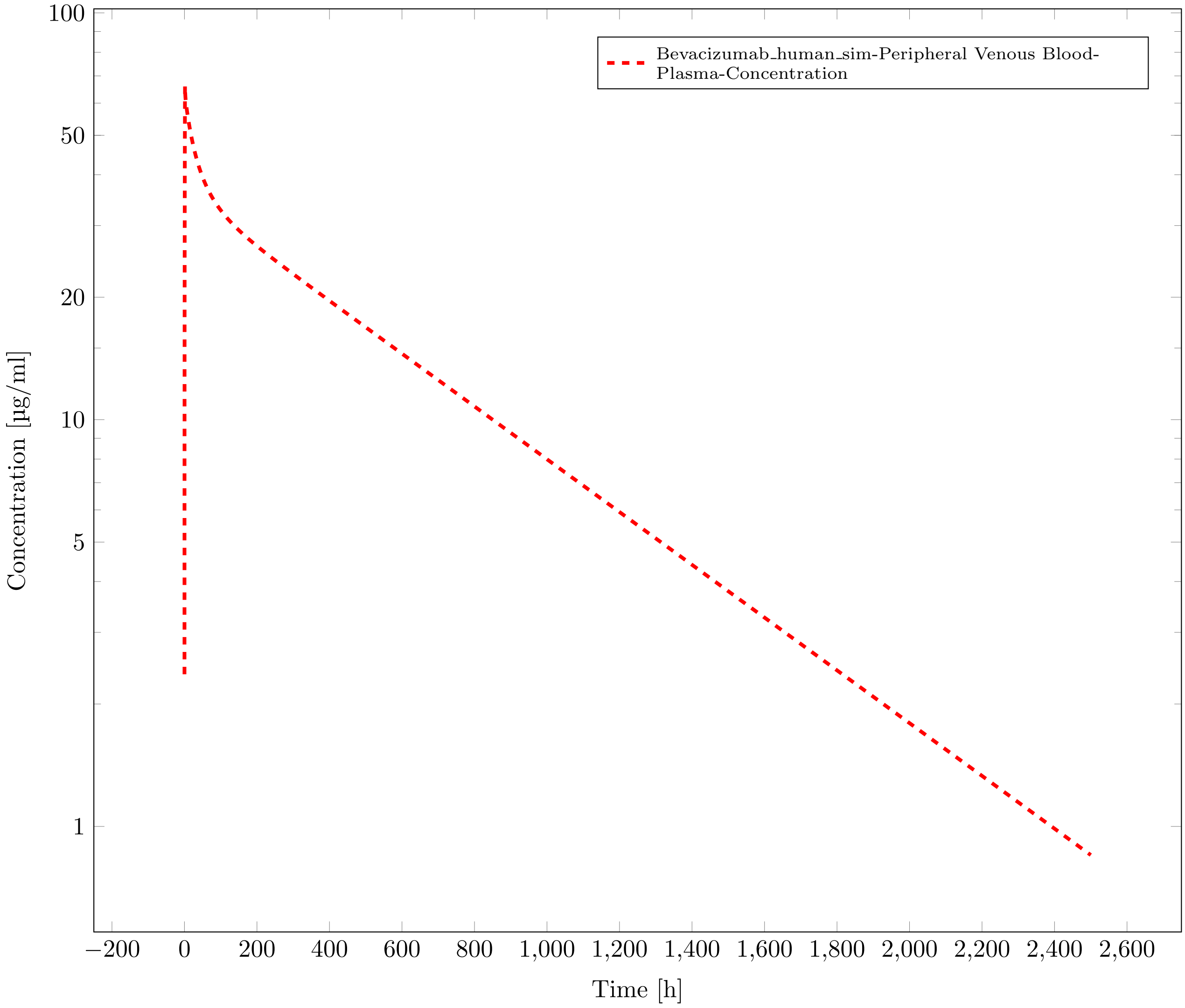

Figure 4.1

4.3.1.1 Global PK-Analyses

Parameter	Compound	Value	Unit
Vss (plasma)	Bevacizumab_human_sim	77.98	ml/kg
Vd (plasma)	Bevacizumab_human_sim	81.01	ml/kg
Vss (phys-chem)	Bevacizumab_human_sim	623.05	ml/kg
Total plasma clearance	Bevacizumab_human_sim	2.02×10^{-3}	ml/min/kg

Table 4.5: Global PK-Analyses

4.3.1.2 PK-Analyses

Bevacizumab_human_sim-Peripheral Venous Blood-Plasma-Concentration

Parameter	Value	Unit
C_max	0.44	µmol/l
C_max_norm	2.21×10^{7}	mg/l
t_max	1.50	h
C_tEnd	5.67×10^{-3}	µmol/l
AUC_tEnd	9690.83	µmol*min/l
AUC_tEnd_norm	4.85×10^{14}	µg*min/l
AUC_inf	9918.62	µmol*min/l
AUC_inf_norm	4.96×10^{14}	µg*min/l
MRT	644.51	h
Half-Life	464.10	h
% AUC (tlast-∞)	0.02	
Total body clearance/F	2.02×10^{-3}	ml/min/kg
Vss (plasma)/F	77.98	ml/kg
Vd (plasma)/F	81.01	ml/kg

Table 4.6: PK-Analyses for Bevacizumab_human_sim-Peripheral Venous Blood-Plasma-Concentration

Beva_sinn2021_HV_3mpk..Human.iv perfusion.3.Bevacizumab_human_sim-Bevacizumab_human_-sim-Measurement

Parameter	Value	Unit
C_max	0.56	µmol/l
C_max_norm	2.78×10^{7}	mg/l
t_max	0.94	h
C_tEnd	6.84×10^{-3}	µmol/l
AUC_tEnd	13496.98	µmol*min/l
AUC_tEnd_norm	6.75×10^{14}	µg*min/l
AUC_inf	13722.24	µmol*min/l
AUC_inf_norm	6.86×10^{14}	µg*min/l
MRT	569.85	h
Half-Life	380.52	h
% AUC (tlast-∞)	0.02	
Total body clearance/F	1.46×10^{-3}	ml/min/kg
Vss (plasma)/F	49.83	ml/kg
Vd (plasma)/F	48.01	ml/kg

Table 4.7: PK-Analyses for Beva_sinn2021_HV_3mpk..Human.iv perfusion.3.Bevacizumab_human_sim-Bevacizumab_human_sim-Measurement

Beva_shin2020_HV_3mpk..Human.iv perfusion.3.Bevacizumab_human_sim.3-Bevacizumab_human_-sim-Measurement

Parameter	Value	Unit
C_max	0.39	µmol/l
C_max_norm	1.96×10^{7}	mg/l
t_max	47.73	h
C_tEnd	0.02	µmol/l
AUC_tEnd	12330.29	µmol*min/l
AUC_tEnd_norm	6.17×10^{14}	µg*min/l
AUC_inf	NaN	µmol*min/l
AUC_inf_norm	NaN	µg*min/l
MRT	NaN	h
Half-Life	Infinity	h
% AUC (tlast-∞)	NaN	
Total body clearance/F	NaN	ml/min/kg
Vss (plasma)/F	NaN	ml/kg
Vd (plasma)/F	NaN	ml/kg

Table 4.8: PK-Analyses for Beva_shin2020_HV_3mpk..Human.iv perfusion.3.Bevacizumab_human_sim.3-Bevacizumab_human_sim-Measurement

Beva_shin2020_HV_3mpk..Human.iv perfusion.3.Bevacizumab_human_sim.2-Bevacizumab_human_-sim-Measurement

Parameter	Value	Unit
C_max	0.27	µmol/l
C_max_norm	1.37×10^{7}	mg/l
t_max	47.73	h
C_tEnd	7.79×10^{-3}	µmol/l
AUC_tEnd	7591.91	µmol*min/l
AUC_tEnd_norm	3.80×10^{14}	µg*min/l
AUC_inf	NaN	µmol*min/l
AUC_inf_norm	NaN	µg*min/l
MRT	NaN	h
Half-Life	Infinity	h
% AUC (tlast-∞)	NaN	
Total body clearance/F	NaN	ml/min/kg
Vss (plasma)/F	NaN	ml/kg
Vd (plasma)/F	NaN	ml/kg

Table 4.9: PK-Analyses for Beva_shin2020_HV_3mpk..Human.iv perfusion.3.Bevacizumab_human_sim.2-Bevacizumab_human_sim-Measurement

Beva_shin2020_HV_3mpk..Human.iv perfusion.3.Bevacizumab_human_sim.1-Bevacizumab_human_-sim-Measurement

Parameter	Value	Unit
C_max	0.50	µmol/l

continued on next page

continued from previous page		
**Parameter**	**Value**	**Unit**
C_max_norm	2.51×10^{7}	mg/l
t_max	1.50	h
C_tEnd	0.01	µmol/l
AUC_tEnd	11014.24	µmol*min/l
AUC_tEnd_norm	5.51×10^{14}	µg*min/l
AUC_inf	NaN	µmol*min/l
AUC_inf_norm	NaN	µg*min/l
MRT	NaN	h
Half-Life	Infinity	h
% AUC (tlast-∞)	NaN	
Total body clearance/F	NaN	ml/min/kg
Vss (plasma)/F	NaN	ml/kg
Vd (plasma)/F	NaN	ml/kg

Table 4.10: PK-Analyses for Beva_shin2020_HV_3mpk..Human.iv perfusion.3.Bevacizumab_human_sim.1-Bevacizumab_human_sim-Measurement

Chapter 5

Sim_HV_0.5mg/kg

5.1 Used building blocks

Building Block	Name
Individual	HV_sim (see section 1.2 in Part I)
Compound	Bevacizumab_human_sim (see section 2.3 in Part I)
Protocol	Perfusion 90 min_0.5mg/kg (see section 3.4 in Part I)

Table 5.1: Building Block

5.2 Simulation Properties

5.2.1 Model Structure

Allow aging
No

Calculation methods

Category	Calculation methods
Endothelial surface areas	Organ vascularization
Body surface area	Mosteller

Table 5.2: Calculation methods

5.2.2 Compounds

5.2.2.1 Bevacizumab_human_sim

Compound Configuration

Parameter	Alternative in compound	Value	Unit
Solubility	Measurement	9999.00	mg/l
Lipophilicity	Measurement	-5.00	Log Units
Fraction unbound (plasma, reference value)	Measurement	1.00	
continued on next page			

continued from previous page			
**Parameter**	**Alternative in compound**	**Value**	**Unit**
Specific organ permeability	Calculated	0	cm/min
Specific intestinal permeability	Calculated	0	cm/min

Table 5.3: Compound Configuration

Calculation methods

Category	Calculation methods
Partition coefficients	PK-Sim Standard
Cellular permeabilities	PK-Sim Standard

Table 5.4: Calculation methods

5.2.3 Administration

5.2.3.1 Bevacizumab_human_sim

Simple protocol
Intravenous Infusion
Dosing interval: Single
Dose: 0.50 mg/kg
Infusion time: 90.00 min

5.3 Charts

5.3.1 Time Profile Analysis

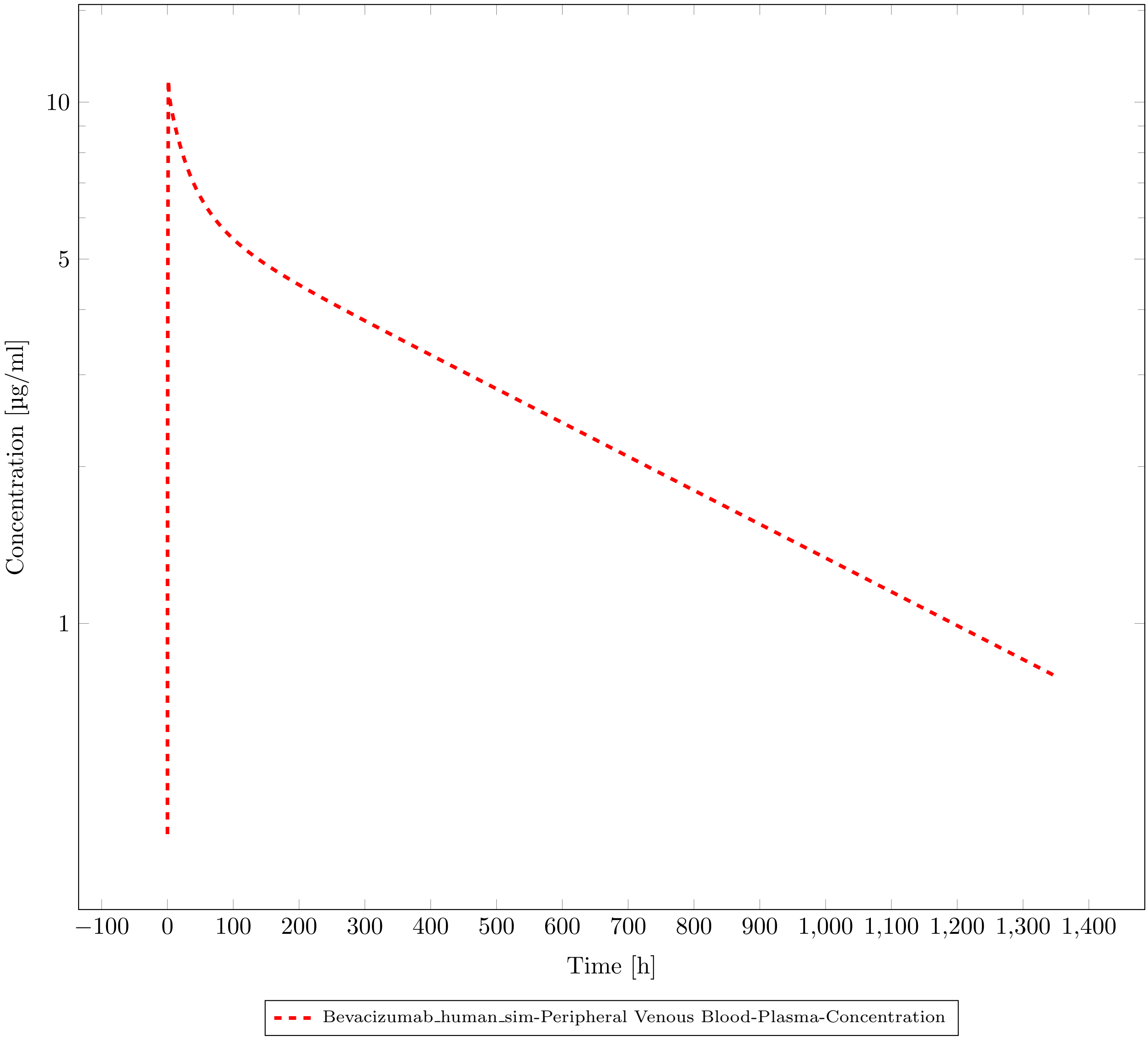

Figure 5.1

5.3.1.1 Global PK-Analyses

Parameter	Compound	Value	Unit
Vss (plasma)	Bevacizumab_human_sim	77.84	ml/kg
Vd (plasma)	Bevacizumab_human_sim	80.84	ml/kg
Vss (phys-chem)	Bevacizumab_human_sim	623.05	ml/kg
Total plasma clearance	Bevacizumab_human_sim	2.01×10^{-3}	ml/min/kg

Table 5.5: Global PK-Analyses

5.3.1.2 PK-Analyses

Bevacizumab_human_sim-Peripheral Venous Blood-Plasma-Concentration

Parameter	Value	Unit
C_max	0.07	µmol/l
C_max_norm	2.21×10^{7}	mg/l
t_max	1.50	h
C_tEnd	5.28×10^{-3}	µmol/l
AUC_tEnd	1444.42	µmol*min/l
AUC_tEnd_norm	4.33×10^{14}	µg*min/l
AUC_inf	1656.48	µmol*min/l
AUC_inf_norm	4.97×10^{14}	µg*min/l
MRT	644.74	h
Half-Life	464.11	h
% AUC (tlast-∞)	0.13	
Total body clearance/F	2.01×10^{-3}	ml/min/kg
Vss (plasma)/F	77.84	ml/kg
Vd (plasma)/F	80.84	ml/kg

Table 5.6: PK-Analyses for Bevacizumab_human_sim-Peripheral Venous Blood-Plasma-Concentration

Beva_li2017_HV_0.5mpk..Human.iv perfusion.0.5.Bevacizumab_human_sim.1-Bevacizumab_human_-sim-Measurement

Parameter	Value	Unit
C_max	0.04	µmol/l
C_max_norm	1.35×10^{7}	mg/l
t_max	2.00	h
C_tEnd	1.00×10^{-3}	µmol/l
AUC_tEnd	889.66	µmol*min/l
AUC_tEnd_norm	2.67×10^{14}	µg*min/l
AUC_inf	NaN	µmol*min/l
AUC_inf_norm	NaN	µg*min/l
MRT	NaN	h
Half-Life	Infinity	h
% AUC (tlast-∞)	NaN	
Total body clearance/F	NaN	ml/min/kg
Vss (plasma)/F	NaN	ml/kg
Vd (plasma)/F	NaN	ml/kg

Table 5.7: PK-Analyses for Beva_li2017_HV_0.5mpk..Human.iv perfusion.0.5.Bevacizumab_human_sim.1-Bevacizumab_human_sim-Measurement

Beva_li2017_HV_0.5mpk..Human.iv perfusion.0.5.Bevacizumab_human_sim.2-Bevacizumab_human_-sim-Measurement

Parameter	Value	Unit
C_max	0.04	µmol/l
C_max_norm	1.12×10^{7}	mg/l
t_max	2.00	h
C_tEnd	5.21×10^{-4}	µmol/l
AUC_tEnd	538.67	µmol*min/l
AUC_tEnd_norm	1.62×10^{14}	µg*min/l
AUC_inf	NaN	µmol*min/l
AUC_inf_norm	NaN	µg*min/l
MRT	NaN	h
Half-Life	Infinity	h
% AUC (tlast-∞)	NaN	
Total body clearance/F	NaN	ml/min/kg
Vss (plasma)/F	NaN	ml/kg
Vd (plasma)/F	NaN	ml/kg

Table 5.8: PK-Analyses for Beva_li2017_HV_0.5mpk..Human.iv perfusion.0.5.Bevacizumab_human_sim.2-Bevacizumab_human_sim-Measurement

Beva_li2017_HV_0.5mpk..Human.iv perfusion.0.5.Bevacizumab_human_sim.3-Bevacizumab_human_-sim-Measurement

Parameter	Value	Unit
C_max	0.05	µmol/l
C_max_norm	1.63×10^{7}	mg/l
t_max	2.00	h
C_tEnd	7.56×10^{-4}	µmol/l
AUC_tEnd	1054.14	µmol*min/l
AUC_tEnd_norm	3.16×10^{14}	µg*min/l
AUC_inf	NaN	µmol*min/l
AUC_inf_norm	NaN	µg*min/l
MRT	NaN	h
Half-Life	Infinity	h
% AUC (tlast-∞)	NaN	
Total body clearance/F	NaN	ml/min/kg
Vss (plasma)/F	NaN	ml/kg
Vd (plasma)/F	NaN	ml/kg

Table 5.9: PK-Analyses for Beva_li2017_HV_0.5mpk..Human.iv perfusion.0.5.Bevacizumab_human_sim.3-Bevacizumab_human_sim-Measurement

Beva_li2017_HV_0.5mpk..Human.iv perfusion.0.5.Bevacizumab_human_sim.4-Bevacizumab_human_-sim-Measurement

Parameter	Value	Unit
C_max	0.05	µmol/l
		continued on next page

continued from previous page		
**Parameter**	**Value**	**Unit**
C_max_norm	1.50×10^{7}	mg/l
t_max	2.00	h
C_tEnd	1.17×10^{-3}	µmol/l
AUC_tEnd	914.90	µmol*min/l
AUC_tEnd_norm	2.74×10^{14}	µg*min/l
AUC_inf	NaN	µmol*min/l
AUC_inf_norm	NaN	µg*min/l
MRT	NaN	h
Half-Life	Infinity	h
% AUC (tlast-∞)	NaN	
Total body clearance/F	NaN	ml/min/kg
Vss (plasma)/F	NaN	ml/kg
Vd (plasma)/F	NaN	ml/kg

Table 5.10: PK-Analyses for Beva_li2017_HV_0.5mpk..Human.iv perfusion.0.5.Bevacizumab_human_sim.4-Bevacizumab_human_sim-Measurement

Beva_li2017_HV_0.5mpk..Human.iv perfusion.0.5.Bevacizumab_human_sim.5-Bevacizumab_human_-sim-Measurement

Parameter	Value	Unit
C_max	0.05	µmol/l
C_max_norm	1.37×10^{7}	mg/l
t_max	2.00	h
C_tEnd	1.08×10^{-3}	µmol/l
AUC_tEnd	766.72	µmol*min/l
AUC_tEnd_norm	2.30×10^{14}	µg*min/l
AUC_inf	NaN	µmol*min/l
AUC_inf_norm	NaN	µg*min/l
MRT	NaN	h
Half-Life	Infinity	h
% AUC (tlast-∞)	NaN	
Total body clearance/F	NaN	ml/min/kg
Vss (plasma)/F	NaN	ml/kg
Vd (plasma)/F	NaN	ml/kg

Table 5.11: PK-Analyses for Beva_li2017_HV_0.5mpk..Human.iv perfusion.0.5.Bevacizumab_human_sim.5-Bevacizumab_human_sim-Measurement

Chapter 6

Sim_HV_0.5mg/kg_VEGF

6.1 Used building blocks

Building Block	Name
Individual	HV_sim_VEGF (see section 1.3 in Part I)
Compound	Bevacizumab_human_sim (see section 2.3 in Part I)
Protocol	Perfusion 90 min_0.5mg/kg (see section 3.4 in Part I)

Table 6.1: Building Block

6.2 Simulation Properties

6.2.1 Model Structure

Allow aging
No

Calculation methods

Category	Calculation methods
Endothelial surface areas	Organ vascularization
Body surface area	Mosteller

Table 6.2: Calculation methods

6.2.2 Compounds

6.2.2.1 Bevacizumab_human_sim

Compound Configuration

Parameter	Alternative in compound	Value	Unit
Solubility	Measurement	9999.00	mg/l
Lipophilicity	Measurement	−5.00	Log Units
Fraction unbound (plasma, reference value)	Measurement	1.00	
			continued on next page

continued from previous page			
**Parameter**	**Alternative in compound**	**Value**	**Unit**
Specific organ permeability	Calculated	0	cm/min
Specific intestinal permeability	Calculated	0	cm/min

Table 6.3: Compound Configuration

Calculation methods

Category	Calculation methods
Partition coefficients	PK-Sim Standard
Cellular permeabilities	PK-Sim Standard

Table 6.4: Calculation methods

6.2.3 Processes

6.2.3.1 Bevacizumab_human_sim

Specific Binding
Mapping VEGFA with VEGFA-Paper

6.2.4 Administration

6.2.4.1 Bevacizumab_human_sim

Simple protocol
Intravenous Infusion
Dosing interval: Single
Dose: 0.50 mg/kg
Infusion time: 90.00 min

6.3 Charts

6.3.1 Time Profile Analysis

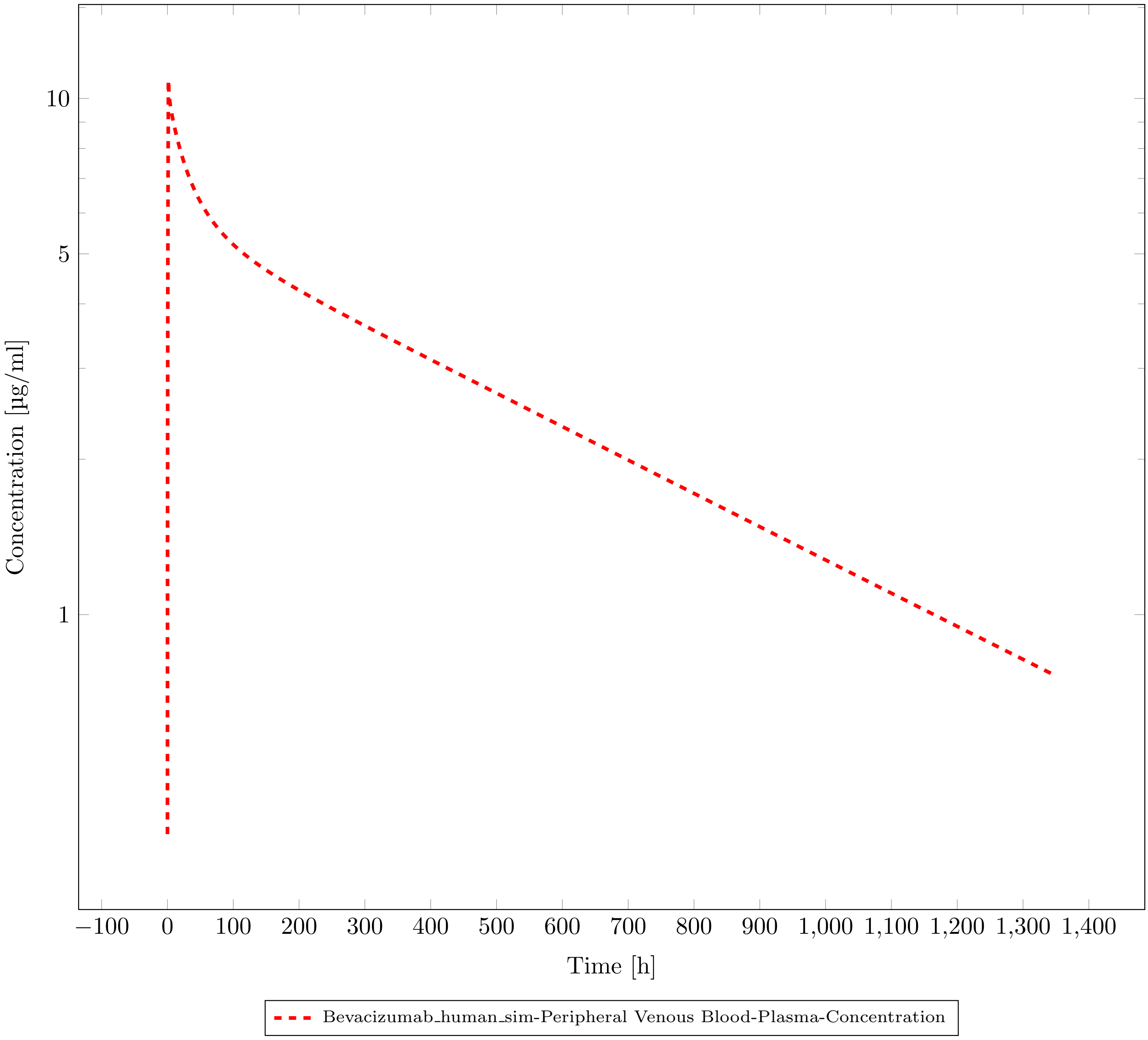

Figure 6.1

6.3.1.1 Global PK-Analyses

Parameter	Compound	Value	Unit
Vss (plasma)	Bevacizumab_human_sim	81.96	ml/kg
Vd (plasma)	Bevacizumab_human_sim	85.87	ml/kg
Vss (phys-chem)	Bevacizumab_human_sim	623.05	ml/kg
Total plasma clearance	Bevacizumab_human_sim	2.10×10^{-3}	ml/min/kg

Table 6.5: Global PK-Analyses

6.3.1.2 PK-Analyses

Bevacizumab_human_sim-Peripheral Venous Blood-Plasma-Concentration

Parameter	Value	Unit
C_max	0.07	µmol/l
C_max_norm	2.17×10^{7}	mg/l
t_max	1.50	h
C_tEnd	5.07×10^{-3}	µmol/l
AUC_tEnd	1379.55	µmol*min/l
AUC_tEnd_norm	4.14×10^{14}	µg*min/l
AUC_inf	1586.95	µmol*min/l
AUC_inf_norm	4.76×10^{14}	µg*min/l
MRT	650.30	h
Half-Life	472.26	h
% AUC (tlast-∞)	0.13	
Total body clearance/F	2.10×10^{-3}	ml/min/kg
Vss (plasma)/F	81.96	ml/kg
Vd (plasma)/F	85.87	ml/kg

Table 6.6: PK-Analyses for Bevacizumab_human_sim-Peripheral Venous Blood-Plasma-Concentration

Beva_li2017_HV_0.5mpk..Human.iv perfusion.0.5.Bevacizumab_human_sim.1-Bevacizumab_human_-sim-Measurement

Parameter	Value	Unit
C_max	0.04	µmol/l
C_max_norm	1.35×10^{7}	mg/l
t_max	2.00	h
C_tEnd	1.00×10^{-3}	µmol/l
AUC_tEnd	889.66	µmol*min/l
AUC_tEnd_norm	2.67×10^{14}	µg*min/l
AUC_inf	NaN	µmol*min/l
AUC_inf_norm	NaN	µg*min/l
MRT	NaN	h
Half-Life	Infinity	h
% AUC (tlast-∞)	NaN	
Total body clearance/F	NaN	ml/min/kg
Vss (plasma)/F	NaN	ml/kg
Vd (plasma)/F	NaN	ml/kg

Table 6.7: PK-Analyses for Beva_li2017_HV_0.5mpk..Human.iv perfusion.0.5.Bevacizumab_human_sim.1-Bevacizumab_human_sim-Measurement

Beva_li2017_HV_0.5mpk..Human.iv perfusion.0.5.Bevacizumab_human_sim.2-Bevacizumab_human_-sim-Measurement

Parameter	Value	Unit
C_max	0.04	µmol/l
C_max_norm	1.12×10^{7}	mg/l
t_max	2.00	h
C_tEnd	5.21×10^{-4}	µmol/l
AUC_tEnd	538.67	µmol*min/l
AUC_tEnd_norm	1.62×10^{14}	µg*min/l
AUC_inf	NaN	µmol*min/l
AUC_inf_norm	NaN	µg*min/l
MRT	NaN	h
Half-Life	Infinity	h
% AUC (tlast-∞)	NaN	
Total body clearance/F	NaN	ml/min/kg
Vss (plasma)/F	NaN	ml/kg
Vd (plasma)/F	NaN	ml/kg

Table 6.8: PK-Analyses for Beva_li2017_HV_0.5mpk..Human.iv perfusion.0.5.Bevacizumab_human_sim.2-Bevacizumab_human_sim-Measurement

Beva_li2017_HV_0.5mpk..Human.iv perfusion.0.5.Bevacizumab_human_sim.3-Bevacizumab_human_-sim-Measurement

Parameter	Value	Unit
C_max	0.05	µmol/l
C_max_norm	1.63×10^{7}	mg/l
t_max	2.00	h
C_tEnd	7.56×10^{-4}	µmol/l
AUC_tEnd	1054.14	µmol*min/l
AUC_tEnd_norm	3.16×10^{14}	µg*min/l
AUC_inf	NaN	µmol*min/l
AUC_inf_norm	NaN	µg*min/l
MRT	NaN	h
Half-Life	Infinity	h
% AUC (tlast-∞)	NaN	
Total body clearance/F	NaN	ml/min/kg
Vss (plasma)/F	NaN	ml/kg
Vd (plasma)/F	NaN	ml/kg

Table 6.9: PK-Analyses for Beva_li2017_HV_0.5mpk..Human.iv perfusion.0.5.Bevacizumab_human_sim.3-Bevacizumab_human_sim-Measurement

Beva_li2017_HV_0.5mpk..Human.iv perfusion.0.5.Bevacizumab_human_sim.4-Bevacizumab_human_-sim-Measurement

Parameter	Value	Unit
C_max	0.05	µmol/l

continued on next page

continued from previous page		
**Parameter**	**Value**	**Unit**
C_max_norm	1.50×10^{7}	mg/l
t_max	2.00	h
C_tEnd	1.17×10^{-3}	µmol/l
AUC_tEnd	914.90	µmol*min/l
AUC_tEnd_norm	2.74×10^{14}	µg*min/l
AUC_inf	NaN	µmol*min/l
AUC_inf_norm	NaN	µg*min/l
MRT	NaN	h
Half-Life	Infinity	h
% AUC (tlast-∞)	NaN	
Total body clearance/F	NaN	ml/min/kg
Vss (plasma)/F	NaN	ml/kg
Vd (plasma)/F	NaN	ml/kg

Table 6.10: PK-Analyses for Beva_li2017_HV_0.5mpk..Human.iv perfusion.0.5.Bevacizumab_human_sim.4-Bevacizumab_human_sim-Measurement

Beva_li2017_HV_0.5mpk..Human.iv perfusion.0.5.Bevacizumab_human_sim.5-Bevacizumab_human_-sim-Measurement

Parameter	Value	Unit
C_max	0.05	µmol/l
C_max_norm	1.37×10^{7}	mg/l
t_max	2.00	h
C_tEnd	1.08×10^{-3}	µmol/l
AUC_tEnd	766.72	µmol*min/l
AUC_tEnd_norm	2.30×10^{14}	µg*min/l
AUC_inf	NaN	µmol*min/l
AUC_inf_norm	NaN	µg*min/l
MRT	NaN	h
Half-Life	Infinity	h
% AUC (tlast-∞)	NaN	
Total body clearance/F	NaN	ml/min/kg
Vss (plasma)/F	NaN	ml/kg
Vd (plasma)/F	NaN	ml/kg

Table 6.11: PK-Analyses for Beva_li2017_HV_0.5mpk..Human.iv perfusion.0.5.Bevacizumab_human_sim.5-Bevacizumab_human_sim-Measurement

Chapter 7

Sim_HV_1mg/kg_VEGF

7.1 Used building blocks

Building Block	Name
Individual	HV_sim_VEGF (see section 1.3 in Part I)
Compound	Bevacizumab_human_sim (see section 2.3 in Part I)
Protocol	Perfusion 90 min_1mg/kg (see section 3.2 in Part I)

Table 7.1: Building Block

7.2 Simulation Properties

7.2.1 Model Structure

Allow aging
No

Calculation methods

Category	Calculation methods
Endothelial surface areas	Organ vascularization
Body surface area	Mosteller

Table 7.2: Calculation methods

7.2.2 Compounds

7.2.2.1 Bevacizumab_human_sim

Compound Configuration

Parameter	Alternative in compound	Value	Unit
Solubility	Measurement	9999.00	mg/l
Lipophilicity	Measurement	−5.00	Log Units
Fraction unbound (plasma, reference value)	Measurement	1.00	
continued on next page			

continued from previous page			
**Parameter**	**Alternative in compound**	**Value**	**Unit**
Specific organ permeability	Calculated	0	cm/min
Specific intestinal permeability	Calculated	0	cm/min

Table 7.3: Compound Configuration

Calculation methods

Category	Calculation methods
Partition coefficients	PK-Sim Standard
Cellular permeabilities	PK-Sim Standard

Table 7.4: Calculation methods

7.2.3 Processes

7.2.3.1 Bevacizumab_human_sim

Specific Binding
Mapping VEGFA with VEGFA-Paper

7.2.4 Administration

7.2.4.1 Bevacizumab_human_sim

Simple protocol
Intravenous Infusion
Dosing interval: Single
Dose: 1.00 mg/kg
Infusion time: 90.00 min

7.3 Charts

7.3.1 Time Profile Analysis

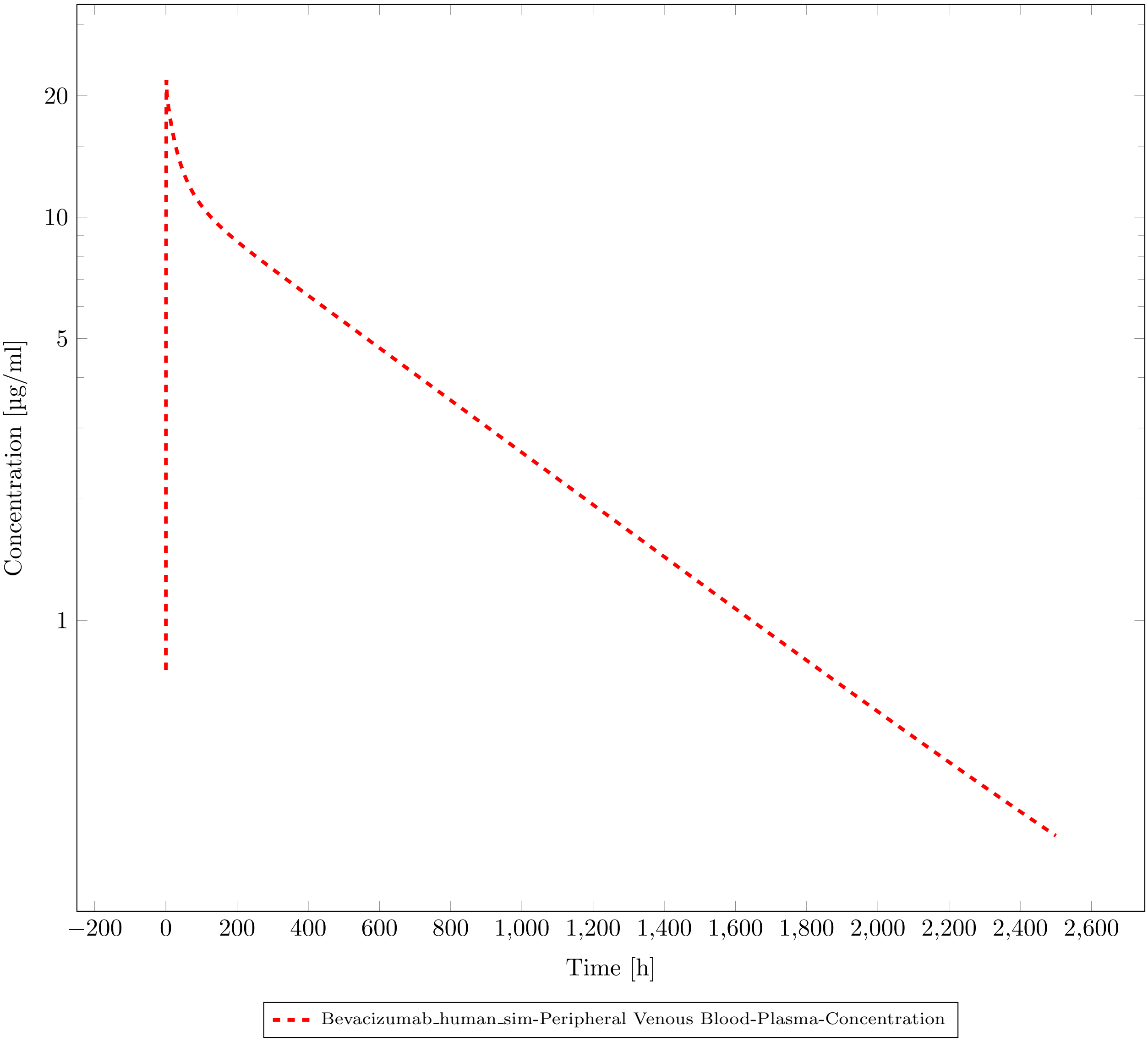

Figure 7.1

7.3.1.1 Global PK-Analyses

Parameter	Compound	Value	Unit
Vss (plasma)	Bevacizumab_human_sim	80.74	ml/kg
Vd (plasma)	Bevacizumab_human_sim	89.79	ml/kg
Vss (phys-chem)	Bevacizumab_human_sim	623.05	ml/kg
Total plasma clearance	Bevacizumab_human_sim	2.05×10^{-3}	ml/min/kg

Table 7.5: Global PK-Analyses

7.3.1.2 PK-Analyses

Bevacizumab_human_sim-Peripheral Venous Blood-Plasma-Concentration

Parameter	Value	Unit
C_max	0.15	µmol/l
C_max_norm	2.19×10^{7}	mg/l
t_max	1.50	h
C_tEnd	1.95×10^{-3}	µmol/l
AUC_tEnd	3165.25	µmol*min/l
AUC_tEnd_norm	4.75×10^{14}	µg*min/l
AUC_inf	3250.56	µmol*min/l
AUC_inf_norm	4.88×10^{14}	µg*min/l
MRT	656.16	h
Half-Life	505.79	h
% AUC (tlast-∞)	0.03	
Total body clearance/F	2.05×10^{-3}	ml/min/kg
Vss (plasma)/F	80.74	ml/kg
Vd (plasma)/F	89.79	ml/kg

Table 7.6: PK-Analyses for Bevacizumab_human_sim-Peripheral Venous Blood-Plasma-Concentration

Beva_Demarchi2021_HV_1mpk..Human.iv perfusion.1.Bevacizumab_human_sim-Bevacizumab_human_-sim-Measurement

Parameter	Value	Unit
C_max	0.15	µmol/l
C_max_norm	2.30×10^{7}	mg/l
t_max	4.86	h
C_tEnd	7.71×10^{-5}	µmol/l
AUC_tEnd	2942.18	µmol*min/l
AUC_tEnd_norm	4.41×10^{14}	µg*min/l
AUC_inf	2942.77	µmol*min/l
AUC_inf_norm	4.41×10^{14}	µg*min/l
MRT	448.08	h
Half-Life	89.57	h
% AUC (tlast-∞)	2.03×10^{-4}	
Total body clearance/F	2.27×10^{-3}	ml/min/kg
Vss (plasma)/F	60.91	ml/kg
Vd (plasma)/F	17.56	ml/kg

Table 7.7: PK-Analyses for Beva_Demarchi2021_HV_1mpk..Human.iv perfusion.1.Bevacizumab_human_sim-Bevacizumab_human_sim-Measurement

Beva_Hetema2017_HV_1mpk..Human.iv perfusion.1.Bevacizumab_human_sim-Bevacizumab_human_-sim-Measurement

Parameter	Value	Unit
C_max	0.16	µmol/l
C_max_norm	2.45×10^{7}	mg/l
t_max	0.64	h
C_tEnd	8.38×10^{-3}	µmol/l
AUC_tEnd	2638.13	µmol*min/l
AUC_tEnd_norm	3.96×10^{14}	µg*min/l
AUC_inf	NaN	µmol*min/l
AUC_inf_norm	NaN	µg*min/l
MRT	NaN	h
Half-Life	Infinity	h
% AUC (tlast-∞)	NaN	
Total body clearance/F	NaN	ml/min/kg
Vss (plasma)/F	NaN	ml/kg
Vd (plasma)/F	NaN	ml/kg

Table 7.8: PK-Analyses for Beva_Hetema2017_HV_1mpk..Human.iv perfusion.1.Bevacizumab_human_sim-Bevacizumab_human_sim-Measurement

Beva_Hummel2022_HV_1mpk..Human.iv perfusion.1.Bevacizumab_human_sim-Bevacizumab_human_-sim-Measurement

Parameter	Value	Unit
C_max	0.17	µmol/l
C_max_norm	2.50×10^{7}	mg/l
t_max	1.50	h
C_tEnd	6.47×10^{-4}	µmol/l
AUC_tEnd	3230.16	µmol*min/l
AUC_tEnd_norm	4.85×10^{14}	µg*min/l
AUC_inf	3241.46	µmol*min/l
AUC_inf_norm	4.86×10^{14}	µg*min/l
MRT	476.41	h
Half-Life	201.77	h
% AUC (tlast-∞)	3.49×10^{-3}	
Total body clearance/F	2.06×10^{-3}	ml/min/kg
Vss (plasma)/F	58.79	ml/kg
Vd (plasma)/F	35.92	ml/kg

Table 7.9: PK-Analyses for Beva_Hummel2022_HV_1mpk..Human.iv perfusion.1.Bevacizumab_human_sim-Bevacizumab_human_sim-Measurement

Beva_Wang2019_HV_1mpk..Human.iv perfusion.1.Bevacizumab_human_sim-Bevacizumab_human_-sim-Measurement

Parameter	Value	Unit
C_max	0.11	µmol/l

continued on next page

continued from previous page		
**Parameter**	**Value**	**Unit**
C_max_norm	1.70×10^{7}	mg/l
t_max	1.50	h
C_tEnd	1.38×10^{-3}	µmol/l
AUC_tEnd	2209.27	µmol*min/l
AUC_tEnd_norm	3.31×10^{14}	µg*min/l
AUC_inf	6061.43	µmol*min/l
AUC_inf_norm	9.09×10^{14}	µg*min/l
MRT	31340.36	h
Half-Life	32359.70	h
% AUC (tlast-∞)	0.64	
Total body clearance/F	1.10×10^{-3}	ml/min/kg
Vss (plasma)/F	2068.18	ml/kg
Vd (plasma)/F	3080.80	ml/kg

Table 7.10: PK-Analyses for Beva_Wang2019_HV_1mpk..Human.iv perfusion.1.Bevacizumab_human_sim-Bevacizumab_human_sim-Measurement

Beva_wu2019_HV_1mpk..Human.iv perfusion.1.Bevacizumab_human_sim.1-Bevacizumab_human_-sim-Measurement

Parameter	Value	Unit
C_max	0.17	µmol/l
C_max_norm	2.50×10^{7}	mg/l
t_max	1.50	h
C_tEnd	1.18×10^{-3}	µmol/l
AUC_tEnd	2779.89	µmol*min/l
AUC_tEnd_norm	4.17×10^{14}	µg*min/l
AUC_inf	2821.02	µmol*min/l
AUC_inf_norm	4.23×10^{14}	µg*min/l
MRT	532.48	h
Half-Life	404.25	h
% AUC (tlast-∞)	0.01	
Total body clearance/F	2.36×10^{-3}	ml/min/kg
Vss (plasma)/F	75.50	ml/kg
Vd (plasma)/F	82.70	ml/kg

Table 7.11: PK-Analyses for Beva_wu2019_HV_1mpk..Human.iv perfusion.1.Bevacizumab_human_sim.1-Bevacizumab_human_sim-Measurement

Beva_wu2019_HV_1mpk..Human.iv perfusion.1.Bevacizumab_human_sim.2-Bevacizumab_human_-sim-Measurement

Parameter	Value	Unit
C_max	0.12	µmol/l
		continued on next page

continued from previous page		
**Parameter**	**Value**	**Unit**
C_max_norm	1.81×10^{7}	mg/l
t_max	14.58	h
C_tEnd	2.20×10^{-4}	µmol/l
AUC_tEnd	2257.21	µmol*min/l
AUC_tEnd_norm	3.39×10^{14}	µg*min/l
AUC_inf	NaN	µmol*min/l
AUC_inf_norm	NaN	µg*min/l
MRT	NaN	h
Half-Life	Infinity	h
% AUC (tlast-∞)	NaN	
Total body clearance/F	NaN	ml/min/kg
Vss (plasma)/F	NaN	ml/kg
Vd (plasma)/F	NaN	ml/kg

Table 7.12: PK-Analyses for Beva_wu2019_HV_1mpk..Human.iv perfusion.1.Bevacizumab_human_sim.2-Bevacizumab_human_sim-Measurement

Beva_wu2019_HV_1mpk..Human.iv perfusion.1.Bevacizumab_human_sim.3-Bevacizumab_human_-sim-Measurement

Parameter	Value	Unit
C_max	0.12	µmol/l
C_max_norm	1.81×10^{7}	mg/l
t_max	14.58	h
C_tEnd	2.48×10^{-3}	µmol/l
AUC_tEnd	2995.12	µmol*min/l
AUC_tEnd_norm	4.49×10^{14}	µg*min/l
AUC_inf	NaN	µmol*min/l
AUC_inf_norm	NaN	µg*min/l
MRT	NaN	h
Half-Life	Infinity	h
% AUC (tlast-∞)	NaN	
Total body clearance/F	NaN	ml/min/kg
Vss (plasma)/F	NaN	ml/kg
Vd (plasma)/F	NaN	ml/kg

Table 7.13: PK-Analyses for Beva_wu2019_HV_1mpk..Human.iv perfusion.1.Bevacizumab_human_sim.3-Bevacizumab_human_sim-Measurement

Chapter 8

Sim_HV_3mg/kg_VEGF

8.1 Used building blocks

Building Block	Name
Individual	HV_sim_VEGF (see section 1.3 in Part I)
Compound	Bevacizumab_human_sim (see section 2.3 in Part I)
Protocol	Perfusion 90 min_3mg/kg (see section 3.3 in Part I)

Table 8.1: Building Block

8.2 Simulation Properties

8.2.1 Model Structure

Allow aging
No

Calculation methods

Category	Calculation methods
Endothelial surface areas	Organ vascularization
Body surface area	Mosteller

Table 8.2: Calculation methods

8.2.2 Compounds

8.2.2.1 Bevacizumab_human_sim

Compound Configuration

Parameter	Alternative in compound	Value	Unit
Solubility	Measurement	9999.00	mg/l
Lipophilicity	Measurement	−5.00	Log Units
Fraction unbound (plasma, reference value)	Measurement	1.00	

continued on next page

continued from previous page			
**Parameter**	**Alternative in compound**	**Value**	**Unit**
Specific organ permeability	Calculated	0	cm/min
Specific intestinal permeability	Calculated	0	cm/min

Table 8.3: Compound Configuration

Calculation methods

Category	Calculation methods
Partition coefficients	PK-Sim Standard
Cellular permeabilities	PK-Sim Standard

Table 8.4: Calculation methods

8.2.3 Processes

8.2.3.1 Bevacizumab_human_sim

Specific Binding
Mapping VEGFA with VEGFA-Paper

8.2.4 Administration

8.2.4.1 Bevacizumab_human_sim

Simple protocol
Intravenous Infusion
Dosing interval: Single
Dose: 3.00 mg/kg
Infusion time: 90.00 min

8.3 Charts

8.3.1 Time Profile Analysis

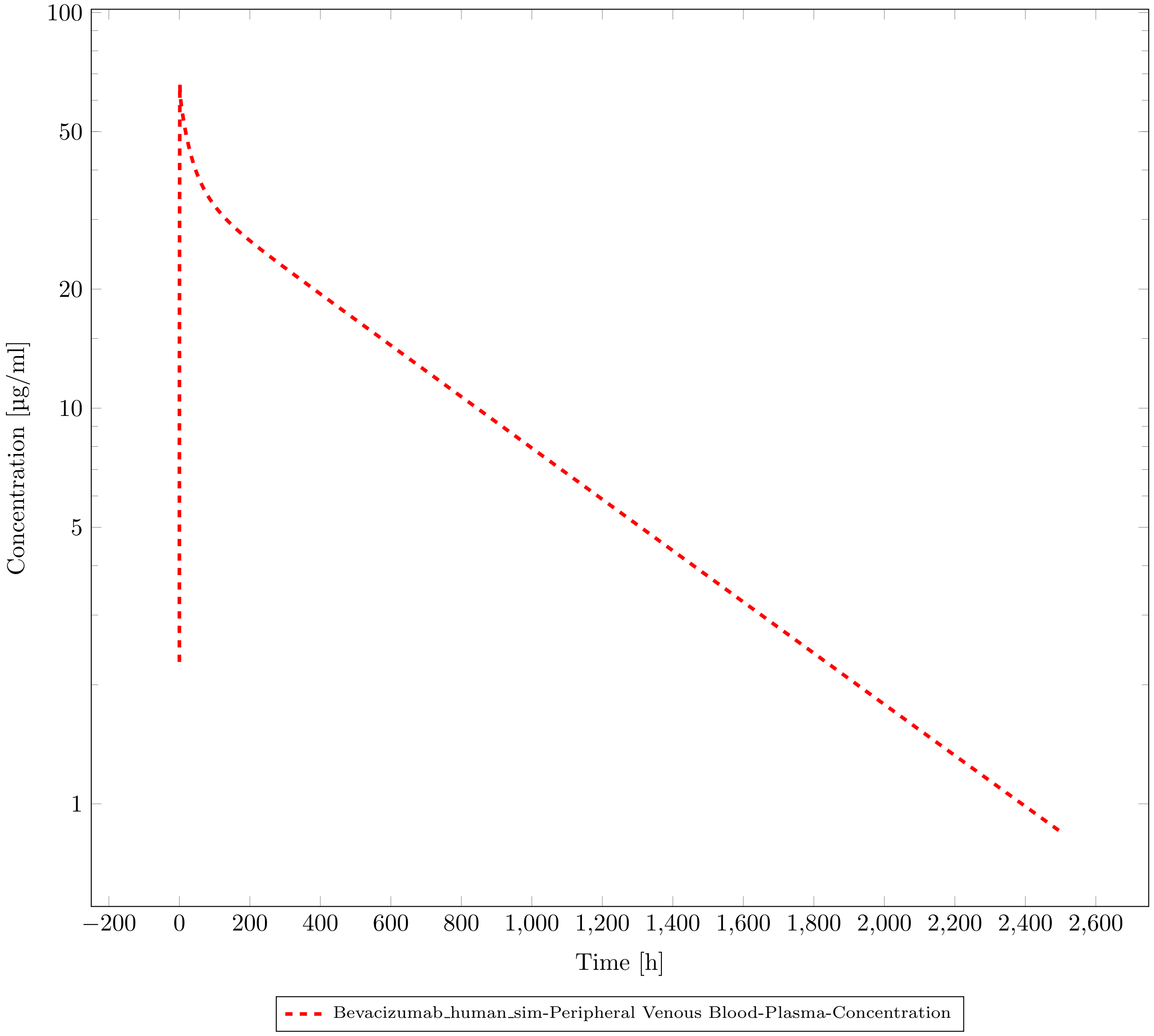

Figure 8.1

8.3.1.1 Global PK-Analyses

Parameter	Compound	Value	Unit
Vss (plasma)	Bevacizumab_human_sim	78.73	ml/kg
Vd (plasma)	Bevacizumab_human_sim	82.77	ml/kg
Vss (phys-chem)	Bevacizumab_human_sim	623.05	ml/kg
Total plasma clearance	Bevacizumab_human_sim	2.03×10^{-3}	ml/min/kg

Table 8.5: Global PK-Analyses

8.3.1.2 PK-Analyses

Bevacizumab_human_sim-Peripheral Venous Blood-Plasma-Concentration

Parameter	Value	Unit
C_max	0.44	µmol/l
C_max_norm	2.20×10^{7}	mg/l
t_max	1.50	h
C_tEnd	5.67×10^{-3}	µmol/l
AUC_tEnd	9617.67	µmol*min/l
AUC_tEnd_norm	4.81×10^{14}	µg*min/l
AUC_inf	9848.75	µmol*min/l
AUC_inf_norm	4.92×10^{14}	µg*min/l
MRT	646.13	h
Half-Life	470.89	h
% AUC (tlast-∞)	0.02	
Total body clearance/F	2.03×10^{-3}	ml/min/kg
Vss (plasma)/F	78.73	ml/kg
Vd (plasma)/F	82.77	ml/kg

Table 8.6: PK-Analyses for Bevacizumab_human_sim-Peripheral Venous Blood-Plasma-Concentration

Beva_sinn2021_HV_3mpk..Human.iv perfusion.3.Bevacizumab_human_sim-Bevacizumab_human_-sim-Measurement

Parameter	Value	Unit
C_max	0.56	µmol/l
C_max_norm	2.78×10^{7}	mg/l
t_max	0.94	h
C_tEnd	6.84×10^{-3}	µmol/l
AUC_tEnd	13496.98	µmol*min/l
AUC_tEnd_norm	6.75×10^{14}	µg*min/l
AUC_inf	13722.24	µmol*min/l
AUC_inf_norm	6.86×10^{14}	µg*min/l
MRT	569.85	h
Half-Life	380.52	h
% AUC (tlast-∞)	0.02	
Total body clearance/F	1.46×10^{-3}	ml/min/kg
Vss (plasma)/F	49.83	ml/kg
Vd (plasma)/F	48.01	ml/kg

Table 8.7: PK-Analyses for Beva_sinn2021_HV_3mpk..Human.iv perfusion.3.Bevacizumab_human_sim-Bevacizumab_human_sim-Measurement

Beva_shin2020_HV_3mpk..Human.iv perfusion.3.Bevacizumab_human_sim.3-Bevacizumab_human_-sim-Measurement

Parameter	Value	Unit
C_max	0.39	µmol/l
C_max_norm	1.96×10^{7}	mg/l
t_max	47.73	h
C_tEnd	0.02	µmol/l
AUC_tEnd	12330.29	µmol*min/l
AUC_tEnd_norm	6.17×10^{14}	µg*min/l
AUC_inf	NaN	µmol*min/l
AUC_inf_norm	NaN	µg*min/l
MRT	NaN	h
Half-Life	Infinity	h
% AUC (tlast-∞)	NaN	
Total body clearance/F	NaN	ml/min/kg
Vss (plasma)/F	NaN	ml/kg
Vd (plasma)/F	NaN	ml/kg

Table 8.8: PK-Analyses for Beva_shin2020_HV_3mpk..Human.iv perfusion.3.Bevacizumab_human_sim.3-Bevacizumab_human_sim-Measurement

Beva_shin2020_HV_3mpk..Human.iv perfusion.3.Bevacizumab_human_sim.2-Bevacizumab_human_-sim-Measurement

Parameter	Value	Unit
C_max	0.27	µmol/l
C_max_norm	1.37×10^{7}	mg/l
t_max	47.73	h
C_tEnd	7.79×10^{-3}	µmol/l
AUC_tEnd	7591.91	µmol*min/l
AUC_tEnd_norm	3.80×10^{14}	µg*min/l
AUC_inf	NaN	µmol*min/l
AUC_inf_norm	NaN	µg*min/l
MRT	NaN	h
Half-Life	Infinity	h
% AUC (tlast-∞)	NaN	
Total body clearance/F	NaN	ml/min/kg
Vss (plasma)/F	NaN	ml/kg
Vd (plasma)/F	NaN	ml/kg

Table 8.9: PK-Analyses for Beva_shin2020_HV_3mpk..Human.iv perfusion.3.Bevacizumab_human_sim.2-Bevacizumab_human_sim-Measurement

Beva_shin2020_HV_3mpk..Human.iv perfusion.3.Bevacizumab_human_sim.1-Bevacizumab_human_-sim-Measurement

Parameter	Value	Unit
C_max	0.50	µmol/l

continued on next page

continued from previous page		
**Parameter**	**Value**	**Unit**
C_max_norm	2.51×10^{7}	mg/l
t_max	1.50	h
C_tEnd	0.01	µmol/l
AUC_tEnd	11014.24	µmol*min/l
AUC_tEnd_norm	5.51×10^{14}	µg*min/l
AUC_inf	NaN	µmol*min/l
AUC_inf_norm	NaN	µg*min/l
MRT	NaN	h
Half-Life	Infinity	h
% AUC (tlast-∞)	NaN	
Total body clearance/F	NaN	ml/min/kg
Vss (plasma)/F	NaN	ml/kg
Vd (plasma)/F	NaN	ml/kg

Table 8.10: PK-Analyses for Beva_shin2020_HV_3mpk..Human.iv perfusion.3.Bevacizumab_human_sim.1-Bevacizumab_human_sim-Measurement

Chapter 9

Monkey_beva_4mpk_vegf

9.1 Used building blocks

Building Block	Name
Individual	monkey_VEGF (see section 1.4 in Part I)
Compound	bevacizumab_monkey_VEGF (see section 2.4 in Part I)
Protocol	Perfusion 60 min_4 mg/kg (see section 3.1 in Part I)

Table 9.1: Building Block

9.2 Simulation Properties

9.2.1 Model Structure

Allow aging
No

Calculation methods

Category	Calculation methods
Endothelial surface areas	Organ vascularization

Table 9.2: Calculation methods

9.2.2 Compounds

9.2.2.1 bevacizumab_monkey_VEGF

Compound Configuration

Parameter	Alternative in compound	Value	Unit
Solubility	Measurement	9999.00	mg/l
Lipophilicity	Measurement	−5.00	Log Units
Fraction unbound (plasma, reference value)	Measurement	1.00	
Specific organ permeability	Calculated	0	cm/min
continued on next page			

continued from previous page			
**Parameter**	**Alternative in compound**	**Value**	**Unit**
Specific intestinal permeability	Calculated	0	cm/min

Table 9.3: Compound Configuration

Calculation methods

Category	Calculation methods
Partition coefficients	PK-Sim Standard
Cellular permeabilities	PK-Sim Standard

Table 9.4: Calculation methods

9.2.3 Processes

9.2.3.1 bevacizumab_monkey_VEGF

Specific Binding
Mapping VEGFA with VEGFA-paper

9.2.4 Administration

9.2.4.1 bevacizumab_monkey_VEGF

Simple protocol
Intravenous Infusion
Dosing interval: Single
Dose: 4.00 mg/kg
Infusion time: 60.00 min

9.3 Charts

9.3.1 Time Profile Analysis

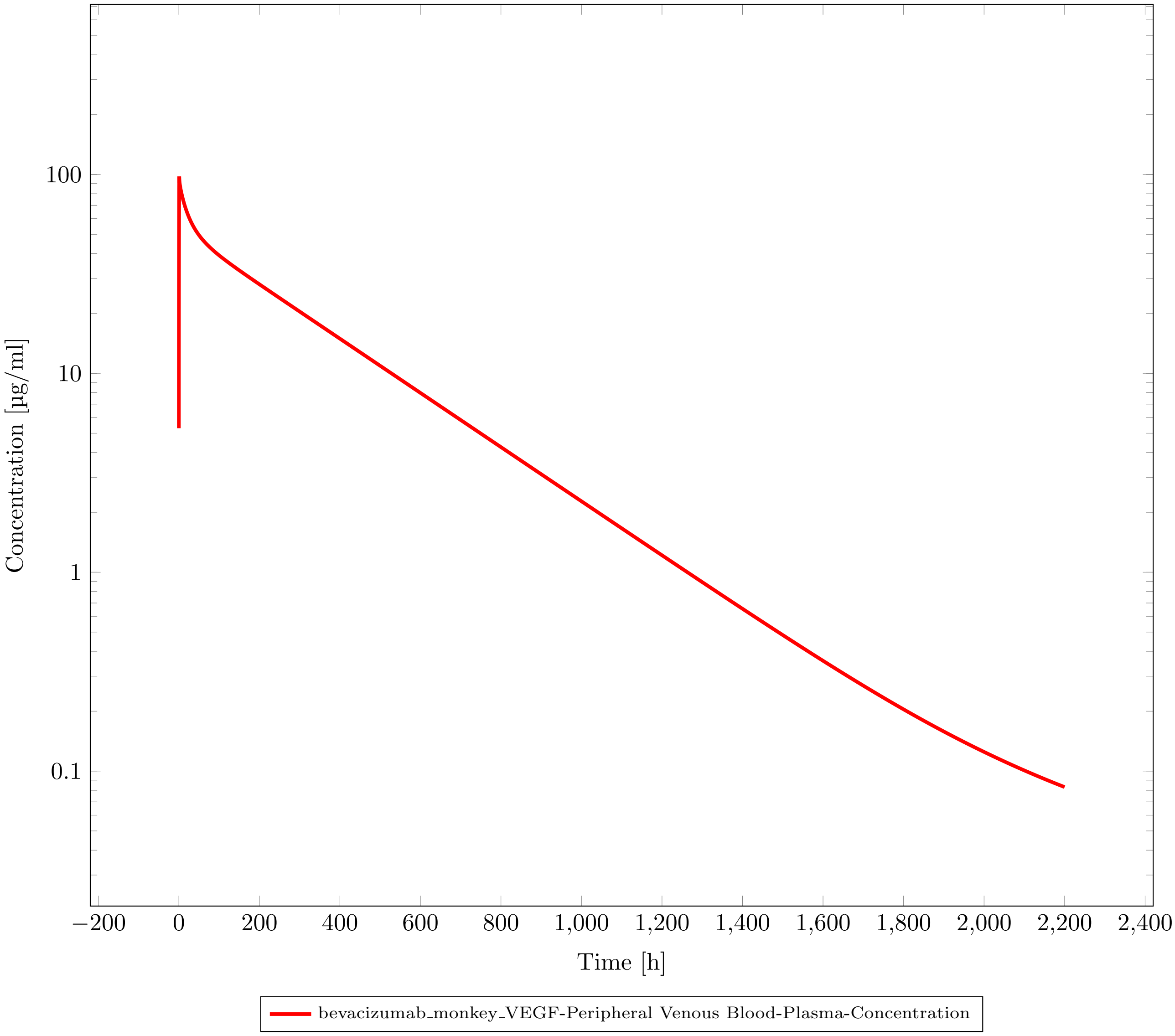

Figure 9.1

9.3.1.1 Global PK-Analyses

Parameter	Compound	Value	Unit
Vss (plasma)	bevacizumab_monkey_VEGF	70.05	ml/kg
Vd (plasma)	bevacizumab_monkey_VEGF	124.41	ml/kg
Vss (phys-chem)	bevacizumab_monkey_VEGF	644.98	ml/kg
Total plasma clearance	bevacizumab_monkey_VEGF	3.78×10^{-3}	ml/min/kg

Table 9.5: Global PK-Analyses

9.3.1.2 PK-Analyses

bevacizumab_monkey_VEGF-Peripheral Venous Blood-Plasma-Concentration

Parameter	Value	Unit
C_max	0.65	µmol/l
C_max_norm	2.45×10^{7}	mg/l
t_max	1.00	h
C_tEnd	5.54×10^{-4}	µmol/l
AUC_tEnd	7040.74	µmol*min/l
AUC_tEnd_norm	2.64×10^{14}	µg*min/l
AUC_inf	7058.98	µmol*min/l
AUC_inf_norm	2.65×10^{14}	µg*min/l
MRT	309.05	h
Half-Life	380.44	h
% AUC (tlast-∞)	2.58×10^{-3}	
Total body clearance/F	3.78×10^{-3}	ml/min/kg
Vss (plasma)/F	70.05	ml/kg
Vd (plasma)/F	124.41	ml/kg

Table 9.6: PK-Analyses for bevacizumab_monkey_VEGF-Peripheral Venous Blood-Plasma-Concentration

Beva_Zalevksy2010_monkey_4mpk..Monkey.iv perfusion.4.bevacizumab_monkey-bevacizumab_monkey-Measurement

Parameter	Value	Unit
C_max	0.77	µmol/l
C_max_norm	NaN	mg/l
t_max	1.92	h
C_tEnd	6.74×10^{-4}	µmol/l
AUC_tEnd	7847.21	µmol*min/l
AUC_tEnd_norm	NaN	µg*min/l
AUC_inf	7863.87	µmol*min/l
AUC_inf_norm	NaN	µg*min/l
MRT	339.82	h
Half-Life	285.61	h
% AUC (tlast-∞)	2.12×10^{-3}	
Total body clearance/F	NaN	ml/min/kg
Vss (plasma)/F	NaN	ml/kg
Vd (plasma)/F	NaN	ml/kg

Table 9.7: PK-Analyses for Beva_Zalevksy2010_monkey_4mpk..Monkey.iv perfusion.4.bevacizumab_monkey-bevacizumab_monkey-Measurement

Part III

Simulation Comparisons

Chapter 1

Comparison_Sim_HV_3mg/kg

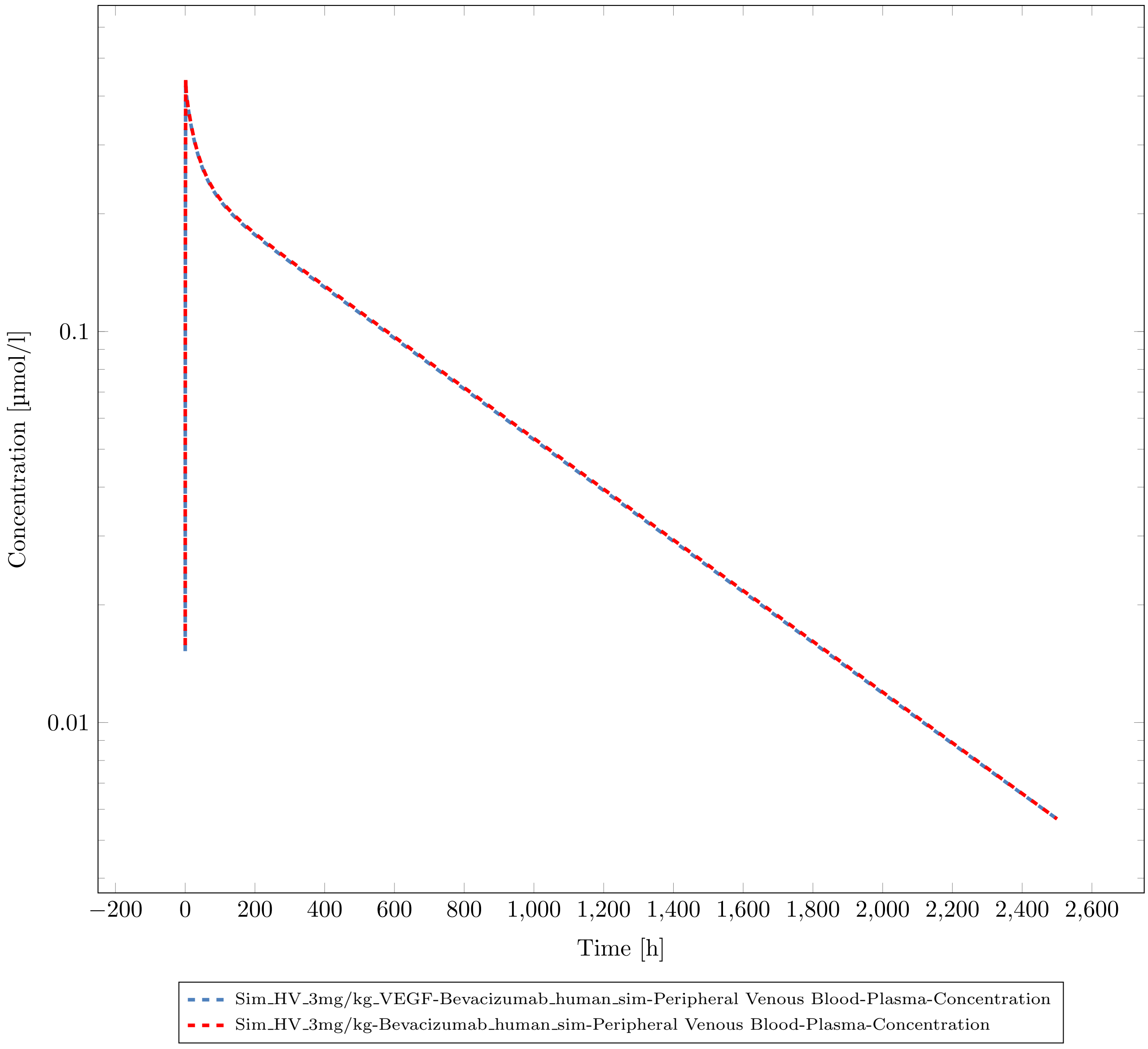

Figure 1.1

Chapter 2

Comparison_Sim_HV_1mg/kg

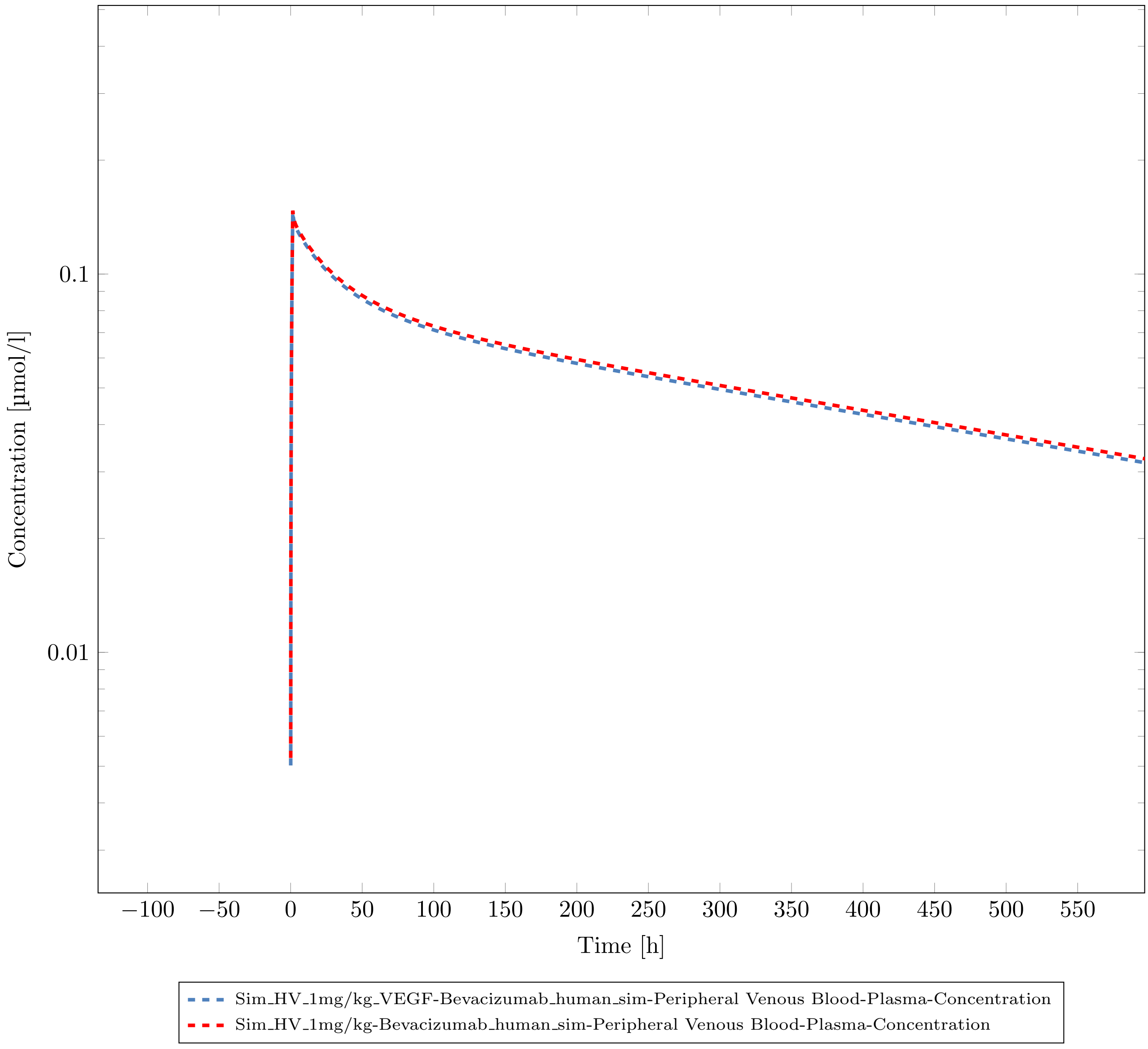

Figure 2.1

Chapter 3

Comparison_Sim_HV_0.5mg/kg

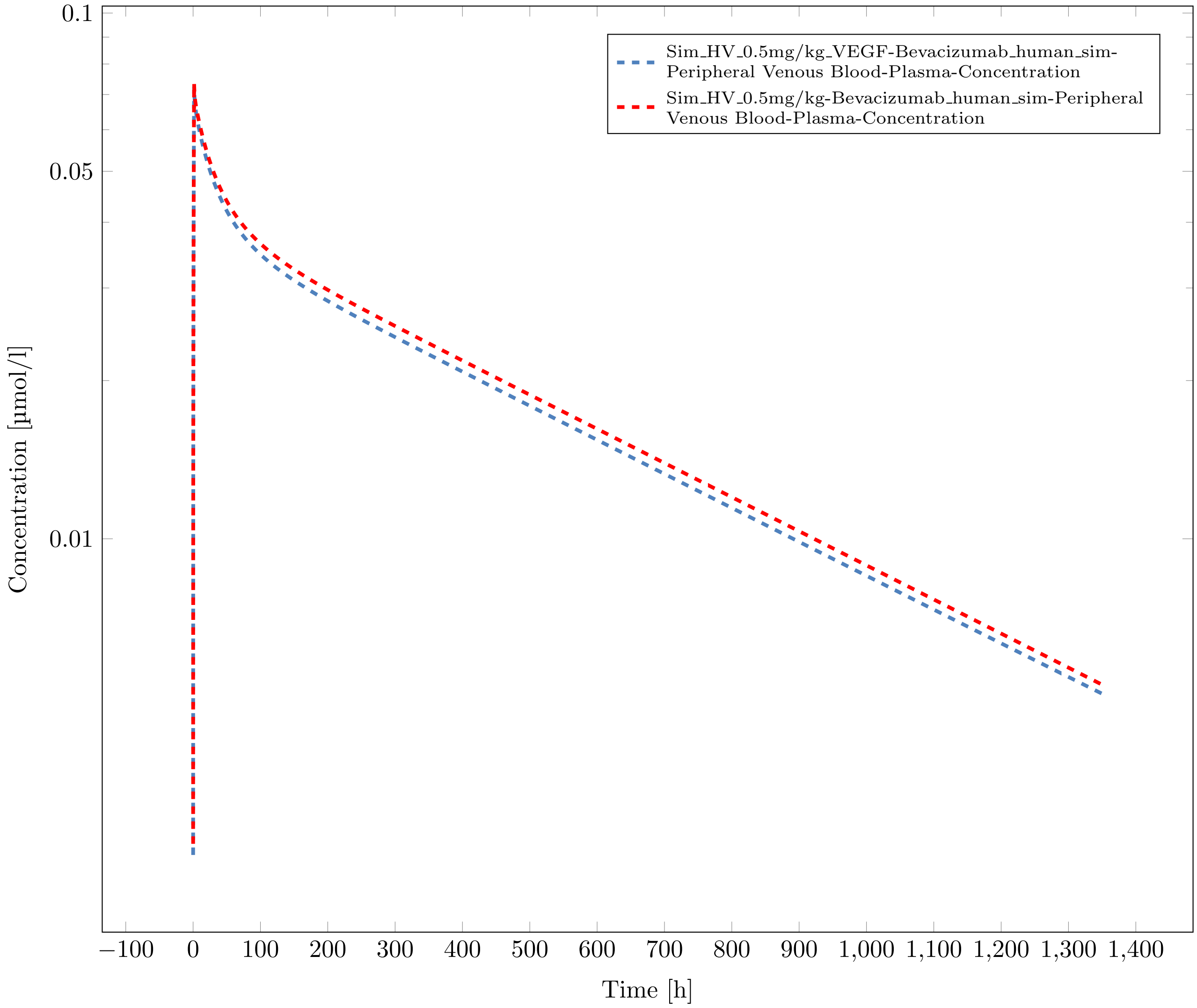

Figure 3.1

Part IV

Observed Data

Chapter 1

Beva_Zalevksy2010_monkey_-4mpk..Monkey.iv perfusion.4.bevacizumab_monkey

Observed Data
Beva_Zalevksy2010_monkey_4mpk..Monkey.iv perfusion.4.bevacizumab_monkey
Source: J:\PhInC\Suivi_Echanges\Stage\Salih Benamara_2022\Gastro Plus\Bevacizumab_all_Raw_Data\Donne propres\Beva_Zalevksy2010_monkey_4mpk.csv
Sheet:
Species: Monkey
Route: iv perfusion
Dose: 4
Molecule: bevacizumab_monkey

Time [h]	Measurement [µg/ml]
1.92	116.08
3.84	103.94
7.68	85.21
23.04	68.33
42.24	54.79
65.28	45.92
90.24	41.13
119.04	38.07
147.84	33.35
170.88	31.56
241.92	25.88
339.84	20.31
410.88	16.11
504.96	11.57
673.92	7.37
840.96	5.91
1011.84	3.60
1178.88	2.14
1347.84	1.22
1512.96	0.65
1681.92	0.39
1848.96	0.20

continued on next page

continued from previous page	
**Time [h]**	**Measurement [µg/ml]**
2135.04	0.10

Table 1.1: Beva_Zalevksy2010_monkey_4mpk

Chapter 2

Beva-xtend_Zalevksy2010_monkey_-4mpk..Monkey.iv perfusion.4.bevacizumab_xtend_monkey

Observed Data
Beva-xtend_Zalevksy2010_monkey_4mpk..Monkey.iv perfusion.4.bevacizumab_xtend_monkey
Source: J:\PhInC\Suivi_Echanges\Stage\Salih Benamara_2022\Gastro Plus\Bevacizumab_all_Raw_Data\Donne propres\Beva-xtend_Zalevksy2010_monkey_4mpk.csv
Sheet:
Species: Monkey
Route: iv perfusion
Dose: 4
Molecule: bevacizumab_xtend_monkey

Time [h]	Measurement [µg/ml]
1.93	122.17
15.44	91.49
25.09	75.72
48.26	62.68
73.35	57.34
98.45	54.84
123.54	51.88
146.70	49.07
169.87	46.94
241.29	44.89
337.80	40.62
409.22	36.75
505.74	32.51
675.60	28.45
843.54	24.08
1013.40	23.29
1177.48	17.83
1345.42	16.31
1515.28	14.44
1683.22	12.49
1853.08	10.57

continued on next page

continued from previous page	
**Time [h]**	**Measurement [µg/ml]**
2142.63	7.83

Table 2.1: Beva-xtend_Zalevksy2010_monke

Chapter 3

Beva_Demarchi2021_HV_-1mpk..Human.iv perfusion.1.Bevacizumab_human_sim

Observed Data
Beva_Demarchi2021_HV_1mpk..Human.iv perfusion.1.Bevacizumab_human_sim
Source: J:\PhInC\Suivi_Echanges\Stage\Salih Benamara_2022\Gastro Plus\Bevacizumab_all_Raw_Data\Donne propres\Beva_Demarchi2021_HV_1mpk.csv
Sheet:
Species: Human
Route: iv perfusion
Dose: 1
Molecule: Bevacizumab_human_sim

**Time [h]**	**Measurement [mg/l]**
0	0.06
0.75	12.00
4.86	23.00
16.21	22.50
25.93	22.00
43.75	19.00
58.34	14.69
71.30	13.08
95.61	12.19
183.12	8.90
358.14	6.06
526.67	5.85
696.83	3.23
862.12	2.47
1030.65	1.84
1199.19	1.41
1534.64	0.76
1704.79	0.49
1871.71	0.27
2278.46	0.01

Table 3.1: Beva_Demarchi2021_HV_1mpk..Hu

Chapter 4

Beva_Hetema2017_HV_-1mpk..Human.iv perfusion.1.Bevacizumab_human_sim

Observed Data
Beva_Hetema2017_HV_1mpk..Human.iv perfusion.1.Bevacizumab_human_sim
Source: J:\PhInC\Suivi_Echanges\Stage\Salih Benamara_2022\Gastro Plus\Bevacizumab_all_Raw_Data\Donne propres\Beva_Hetema2017_HV_1mpk.csv
Sheet:
Species: Human
Route: iv perfusion
Dose: 1
Molecule: Bevacizumab_human_sim

Time [h]	Measurement [mg/l]
0.64	24.50
1.29	22.93
1.93	21.48
2.57	21.06
24.44	18.23
46.95	14.39
91.32	11.39
169.14	9.88
333.78	7.08
502.29	5.12
838.00	2.53
1179.50	1.26

Table 4.1: Beva_Hetema2017_HV_1mpk..Huma

Chapter 5

Beva_Hummel2022_HV_1mpk..Human.iv perfusion.1.Bevacizumab_human_sim

Observed Data
Beva_Hummel2022_HV_1mpk..Human.iv perfusion.1.Bevacizumab_human_sim
Source: J:\PhInC\Suivi_Echanges\Stage\Salih Benamara_2022\Gastro Plus\Bevacizumab_all_Raw_Data\Donne propres\Beva_Hummel2022_HV_1mpk.csv
Sheet:
Species: Human
Route: iv perfusion
Dose: 1
Molecule: Bevacizumab_human_sim

**Time [h]**	**Measurement [mg/l]**
0.33	5.00
1.00	15.00
1.50	25.00
2.00	24.90
3.00	24.80
4.00	24.60
5.00	24.50
6.00	24.40
8.00	24.20
20.05	24.07
30.74	20.16
41.43	17.65
60.14	15.46
81.52	13.73
102.90	12.39
134.97	11.25
163.04	10.30
204.46	9.50
273.95	8.76
350.13	7.61
510.49	5.22
672.19	4.00

continued on next page

continued from previous page	
**Time [h]**	**Measurement [mg/l]**
1014.30	2.20
1348.39	1.15
1679.81	0.63
2013.90	0.31
2353.34	0.10

Table 5.1: Beva_Hummel2022_HV_1mpk..Huma

Chapter 6

Beva_Wang2019_HV_1mpk..Human.iv perfusion.1.Bevacizumab_human_sim

Observed Data
Beva_Wang2019_HV_1mpk..Human.iv perfusion.1.Bevacizumab_human_sim
Source: J:\PhInC\Suivi_Echanges\Stage\Salih Benamara_2022\Gastro Plus\Bevacizumab_all_Raw_Data\Donne propres\Beva_Wang2019_HV_1mpk.csv
Sheet:
Species: Human
Route: iv perfusion
Dose: 1
Molecule: Bevacizumab_human_sim

**Time [h]**	**Measurement [mg/l]**
1.00	8.34
1.50	17.00
2.50	16.00
3.50	15.50
5.18	15.11
9.50	14.50
22.02	13.36
45.33	11.15
93.25	9.23
165.78	6.96
238.31	6.48
334.15	5.40
502.52	3.79
669.59	2.76
839.26	2.08
1006.33	1.52
1343.07	0.81
1679.81	0.41
2015.26	0.21
2353.30	0.21

Table 6.1: Beva_Wang2019_HV_1mpk..Human.

Chapter 7

Beva_wu2019_HV_1mpk..Human.iv perfusion.1.Bevacizumab_human_sim.1

Observed Data
Beva_wu2019_HV_1mpk..Human.iv perfusion.1.Bevacizumab_human_sim.1
Source: J:\PhInC\Suivi_Echanges\Stage\Salih Benamara_2022\Gastro Plus\Bevacizumab_all_Raw_Data\Donne propres\Beva_wu2019_HV_1mpk.csv
Sheet:
Species: Human
Route: iv perfusion
Dose: 1
Molecule: Bevacizumab_human_sim
Subject Id: 1

**Time [h]**	**Measurement [mg/l]**
0.75	12.00
1.50	25.00
2.50	24.50
3.50	24.00
5.50	22.50
9.50	20.00
14.58	18.09
19.92	16.04
46.98	13.86
94.96	11.28
168.77	8.42
237.64	7.40
336.02	6.29
503.26	4.86
671.74	3.69
840.22	2.62
1007.47	1.97
1344.42	1.13
1680.16	0.60
2015.89	0.31
2351.62	0.18

Table 7.1: Beva_wu2019_HV_1mpk..Human.iv

Chapter 8

Beva_wu2019_HV_1mpk..Human.iv perfusion.1.Bevacizumab_human_sim.2

Observed Data
Beva_wu2019_HV_1mpk..Human.iv perfusion.1.Bevacizumab_human_sim.2
Source: J:\PhInC\Suivi_Echanges\Stage\Salih Benamara_2022\Gastro Plus\Bevacizumab_all_Raw_Data\Donne propres\Beva_wu2019_HV_1mpk.csv
Sheet:
Species: Human
Route: iv perfusion
Dose: 1
Molecule: Bevacizumab_human_sim
Subject Id: 2

Time [h]	Measurement [mg/l]
14.58	18.09
19.92	16.04
46.98	13.86
94.96	11.28
168.77	8.42
237.64	7.40
336.02	4.99
503.26	3.92
671.74	2.98
840.22	2.04
1007.47	1.40
1344.42	0.74
1680.16	0.30
2015.89	0.10
2351.62	0.03

Table 8.1: Beva_wu2019_HV_1mpk..Human.iv

Chapter 9

Beva_wu2019_HV_1mpk..Human.iv perfusion.1.Bevacizumab_human_sim.3

Observed Data
Beva_wu2019_HV_1mpk..Human.iv perfusion.1.Bevacizumab_human_sim.3
Source: J:\PhInC\Suivi_Echanges\Stage\Salih Benamara_2022\Gastro Plus\Bevacizumab_all_Raw_Data\Donne propres\Beva_wu2019_HV_1mpk.csv
Sheet:
Species: Human
Route: iv perfusion
Dose: 1
Molecule: Bevacizumab_human_sim
Subject Id: 3

Time [h]	Measurement [mg/l]
14.58	18.09
19.92	16.04
46.98	13.86
94.96	11.28
168.77	8.42
237.64	7.40
336.02	7.66
503.26	5.92
671.74	4.35
840.22	3.14
1007.47	2.26
1344.42	1.35
1680.16	0.65
2015.89	0.53
2351.62	0.37

Table 9.1: Beva_wu2019_HV_1mpk..Human.iv

Chapter 10

Beva_shin2020_HV_3mpk..Human.iv perfusion.3.Bevacizumab_human_sim.1

Observed Data
Beva_shin2020_HV_3mpk..Human.iv perfusion.3.Bevacizumab_human_sim.1
Source: J:\PhInC\Suivi_Echanges\Stage\Salih Benamara_2022\Gastro Plus\Bevacizumab_all_Raw_Data\Donne propres\Beva_shin2020_HV_3mpk.csv
Sheet:
Species: Human
Route: iv perfusion
Dose: 3
Molecule: Bevacizumab_human_sim
Subject Id: 1

Time [h]	Measurement [mg/l]
0.75	33.20
1.50	75.45
4.15	74.26
8.30	65.68
12.45	59.41
24.90	55.56
47.73	49.14
96.49	40.42
168.09	31.80
335.14	23.01
506.33	18.61
672.35	14.89
1006.44	9.21
1344.69	5.83
1679.83	3.69
2016.00	2.14

Table 10.1: Beva_shin2020_HV_3mpk..Human.

Chapter 11

Beva_shin2020_HV_3mpk..Human.iv perfusion.3.Bevacizumab_human_sim.2

Observed Data
Beva_shin2020_HV_3mpk..Human.iv perfusion.3.Bevacizumab_human_sim.2
Source: J:\PhInC\Suivi_Echanges\Stage\Salih Benamara_2022\Gastro Plus\Bevacizumab_all_Raw_Data\Donne propres\Beva_shin2020_HV_3mpk.csv
Sheet:
Species: Human
Route: iv perfusion
Dose: 3
Molecule: Bevacizumab_human_sim
Subject Id: 2

**Time [h]**	**Measurement [mg/l]**
47.73	41.10
96.49	33.25
168.09	25.72
335.14	18.61
506.33	15.06
672.35	11.14
1006.44	6.59
1344.69	3.90
1679.83	2.11
2016.00	1.17

Table 11.1: Beva_shin2020_HV_3mpk..Human.

Chapter 12

Beva_shin2020_HV_3mpk..Human.iv perfusion.3.Bevacizumab_human_sim.3

Observed Data
Beva_shin2020_HV_3mpk..Human.iv perfusion.3.Bevacizumab_human_sim.3
Source: J:\PhInC\Suivi_Echanges\Stage\Salih Benamara_2022\Gastro Plus\Bevacizumab_all_Raw_Data\Donne propres\Beva_shin2020_HV_3mpk.csv
Sheet:
Species: Human
Route: iv perfusion
Dose: 3
Molecule: Bevacizumab_human_sim
Subject Id: 3

**Time [h]**	**Measurement [mg/l]**
47.73	58.75
96.49	45.96
168.09	37.59
335.14	27.20
506.33	22.50
672.35	18.61
1006.44	12.32
1344.69	7.79
1679.83	5.33
2016.00	3.21

Table 12.1: Beva_shin2020_HV_3mpk..Human.

Chapter 13

Beva_sinn2021_HV_3mpk..Human.iv perfusion.3.Bevacizumab_human_sim

Observed Data
Beva_sinn2021_HV_3mpk..Human.iv perfusion.3.Bevacizumab_human_sim
Source: J:\PhInC\Suivi_Echanges\Stage\Salih Benamara_2022\Gastro Plus\Bevacizumab_all_Raw_Data\Donne propres\Beva_sinn2021_HV_3mpk.csv
Sheet:
Species: Human
Route: iv perfusion
Dose: 3
Molecule: Bevacizumab_human_sim

**Time [h]**	**Measurement [mg/l]**
0.94	83.47
2.00	83.00
3.00	82.50
4.00	82.00
5.00	81.00
6.00	80.50
8.00	76.00
19.75	73.15
36.67	64.10
52.65	57.75
78.04	51.32
106.25	49.56
132.57	46.24
159.84	43.43
184.29	41.08
222.84	38.06
301.82	33.59
473.88	24.06
639.37	17.84
984.44	10.60
1307.88	6.38
1840.06	2.70
2370.36	1.03

continued on next page

continued from previous page	
**Time [h]**	**Measurement [mg/l]**

Table 13.1: Beva_sinn2021_HV_3mpk..Human.

Chapter 14

Beva_li2017_HV_0.5mpk..Human.iv perfusion.0.5.Bevacizumab_human_sim.1

Observed Data
Beva_li2017_HV_0.5mpk..Human.iv perfusion.0.5.Bevacizumab_human_sim.1
Source: J:\PhInC\Suivi_Echanges\Stage\Salih Benamara_2022\Gastro Plus\Bevacizumab_all_Raw_Data\Donne propres\Beva_li2017_HV_0.5mpk.csv
Sheet:
Species: Human
Route: iv perfusion
Dose: 0.5
Molecule: Bevacizumab_human_sim
Subject Id: 1

Time [h]	Measurement [mg/l]
2.00	6.74
6.00	6.50
16.73	6.30
24.00	6.00
39.03	4.70
64.13	4.55
114.31	3.64
161.71	3.40
329.00	2.45
663.57	1.32
833.64	0.86
1000.93	0.50
1168.22	0.25
1335.50	0.15

Table 14.1: Beva_li2017_HV_0.5mpk..Human.

Chapter 15

Beva_li2017_HV_0.5mpk..Human.iv perfusion.0.5.Bevacizumab_human_-sim.2

Observed Data
Beva_li2017_HV_0.5mpk..Human.iv perfusion.0.5.Bevacizumab_human_sim.2
Source: J:\PhInC\Suivi_Echanges\Stage\Salih Benamara_2022\Gastro Plus\Bevacizumab_all_Raw_Data\Donne propres\Beva_li2017_HV_0.5mpk.csv
Sheet:
Species: Human
Route: iv perfusion
Dose: 0.5
Molecule: Bevacizumab_human_sim
Subject Id: 2

**Time [h]**	**Measurement [mg/l]**
2.00	5.58
8.38	4.62
12.00	4.70
24.00	4.50
39.11	3.58
64.25	3.28
111.73	3.01
162.01	2.10
326.82	1.37
662.01	0.64
829.61	0.45
1000.00	0.23
1170.39	0.10
1337.99	0.08

Table 15.1: Beva_li2017_HV_0.5mpk..Human.

Chapter 16

Beva_li2017_HV_0.5mpk..Human.iv perfusion.0.5.Bevacizumab_human_sim.3

Observed Data
Beva_li2017_HV_0.5mpk..Human.iv perfusion.0.5.Bevacizumab_human_sim.3
Source: J:\PhInC\Suivi_Echanges\Stage\Salih Benamara_2022\Gastro Plus\Bevacizumab_all_Raw_Data\Donne propres\Beva_li2017_HV_0.5mpk.csv
Sheet:
Species: Human
Route: iv perfusion
Dose: 0.5
Molecule: Bevacizumab_human_sim
Subject Id: 3

Time [h]	Measurement [mg/l]
2.00	8.14
5.50	7.52
19.27	6.40
24.00	6.30
41.28	6.19
66.06	5.21
112.84	4.92
162.39	4.01
330.28	3.26
663.30	1.35
833.94	0.90
999.08	0.51
1166.97	0.21
1337.61	0.11

Table 16.1: Beva_li2017_HV_0.5mpk..Human.

Chapter 17

Beva_li2017_HV_0.5mpk..Human.iv perfusion.0.5.Bevacizumab_human_-sim.4

Observed Data
Beva_li2017_HV_0.5mpk..Human.iv perfusion.0.5.Bevacizumab_human_sim.4
Source: J:\PhInC\Suivi_Echanges\Stage\Salih Benamara_2022\Gastro Plus\Bevacizumab_all_Raw_Data\Donne propres\Beva_li2017_HV_0.5mpk.csv
Sheet:
Species: Human
Route: iv perfusion
Dose: 0.5
Molecule: Bevacizumab_human_sim
Subject Id: 4

Time [h]	Measurement [mg/l]
2.00	7.52
5.51	6.26
13.79	6.05
24.00	6.00
41.36	4.76
66.18	4.55
113.05	4.01
162.68	3.42
330.88	2.51
664.52	1.18
835.48	0.88
1000.92	0.71
1169.12	0.36
1337.32	0.18

Table 17.1: Beva_li2017_HV_0.5mpk..Human.

Chapter 18

Beva_li2017_HV_0.5mpk..Human.iv perfusion.0.5.Bevacizumab_human_-sim.5

Observed Data
Beva_li2017_HV_0.5mpk..Human.iv perfusion.0.5.Bevacizumab_human_sim.5
Source: J:\PhInC\Suivi_Echanges\Stage\Salih Benamara_2022\Gastro Plus\Bevacizumab_all_Raw_Data\Donne propres\Beva_li2017_HV_0.5mpk.csv
Sheet:
Species: Human
Route: iv perfusion
Dose: 0.5
Molecule: Bevacizumab_human_sim
Subject Id: 5

Time [h]	Measurement [mg/l]
2.00	6.85
2.75	5.70
19.27	5.32
24.00	5.20
41.28	4.74
66.06	3.95
115.60	3.40
165.14	3.03
330.28	1.89
663.30	0.98
833.94	0.71
1001.83	0.59
1169.72	0.36
1334.86	0.16

Table 18.1: Beva_li2017_HV_0.5mpk..Human.
