# Supplementary material for: Translation of Monoclonal Antibodies Pharmacokinetics from Animal to Human Using Physiologically Based Modeling in Open Systems Pharmacology (OSP) Suite: A Retrospective Analysis of Bevacizumab
Source: Pharmaceutics. 2023 Aug 14;15(8):2129. doi: 10.3390/pharmaceutics15082129 (PMC10459442; doi:10.3390/pharmaceutics15082129)
Supplement: Supplementary file 1 [file pharmaceutics-15-02129-s001.zip › Supplementary Material S3.pdf]

# Project
# Fit-HV_manuscrit

bpasquiers

August 3, 2023

Generated using PK-Sim® 10.0.257.

# Contents


I Building Blocks 6

1 Individuals 7
1.1 HV1 . . . 7
1.1.1 Biometrics . . . 7
1.1.2 Anatomy & Physiology . . . 8
1.1.3 Expression . . . 8
1.1.3.1 Metabolizing Enzymes . . . 8
1.1.3.2 Transport Proteins . . . 8
1.1.3.3 Protein Binding Partners . . . 8
1.2 HV_chinese . . . 8
1.2.1 Biometrics . . . 8
1.2.2 Anatomy & Physiology . . . 9
1.2.3 Expression . . . 9
1.2.3.1 Metabolizing Enzymes . . . 9
1.2.3.2 Transport Proteins . . . 9
1.2.3.3 Protein Binding Partners . . . 9
1.3 Patient1 . . . 9
1.3.1 Biometrics . . . 9
1.3.2 Anatomy & Physiology . . . 10
1.3.3 Expression . . . 10
1.3.3.1 Metabolizing Enzymes . . . 10
1.3.3.2 Transport Proteins . . . 10
1.3.3.3 Protein Binding Partners . . . 10
1.4 Patient_ped . . . 11
1.4.1 Biometrics . . . 11
1.4.2 Anatomy & Physiology . . . 11
1.4.3 Expression . . . 11
1.4.3.1 Metabolizing Enzymes . . . 11
1.4.3.2 Transport Proteins . . . 11
1.4.3.3 Protein Binding Partners . . . 11

2 Compounds 13
2.1 Bevacizumab_human . . . 13
2.1.1 Basic Physico-chemistry . . . 13
2.1.1.1 Molecular weight . . . 13
2.1.1.2 Lipophilicity . . . 13
2.1.1.3 Fraction unbound (plasma, reference value) . . . 13
2.1.1.4 Solubility . . . 14
2.1.2 ADME . . . 14
2.1.2.1 Absorption . . . 14
2.1.2.2 Distribution . . . 14
2.2 Bevacizumab . . . 15
2.2.1 Basic Physico-chemistry . . . 15
2.2.1.1 Molecular weight . . . 15
2.2.1.2 Lipophilicity . . . 15
2.2.1.3 Fraction unbound (plasma, reference value) . . . 15
2.2.1.4 Solubility . . . 16

2.2.2 ADME . . . 16
2.2.2.1 Absorption . . . 16
2.2.2.2 Distribution . . . 16

**3 Protocols** **18**
3.1 Perfusion 90 min_1mg/kg . . . 18
3.2 Perfusion 90 min_3mg/kg . . . 18
3.3 Perfusion 90 min_0.5mg/kg . . . 19
3.4 Infusion 0.3mg/kg human . . . 19
3.5 Infusion 10mg/kg human . . . 20
3.6 Infusion 1mg/kg human . . . 21
3.7 Infusion 3mg/kg human . . . 22
3.8 5mpk_every2weeks . . . 24
3.9 Infusion_5mg/kg . . . 24
3.10 Infusion_15mg/kg . . . 24

**II Simulations** **25**

**1 HV_1mg/kg** **26**
1.1 Used building blocks . . . 26
1.2 Simulation Properties . . . 26
1.2.1 Model Structure . . . 26
1.2.2 Compounds . . . 26
1.2.2.1 Bevacizumab . . . 26
1.2.3 Processes . . . 27
1.2.3.1 Bevacizumab . . . 27
1.2.4 Administration . . . 27
1.2.4.1 Bevacizumab . . . 27
1.3 Charts . . . 27
1.3.1 Time Profile Analysis . . . 28
1.3.1.1 Global PK-Analyses . . . 28
1.3.1.2 PK-Analyses . . . 28

**2 HV_3mg/kg** **33**
2.1 Used building blocks . . . 33
2.2 Simulation Properties . . . 33
2.2.1 Model Structure . . . 33
2.2.2 Compounds . . . 33
2.2.2.1 Bevacizumab . . . 33
2.2.3 Processes . . . 34
2.2.3.1 Bevacizumab . . . 34
2.2.4 Administration . . . 34
2.2.4.1 Bevacizumab . . . 34
2.3 Charts . . . 34
2.3.1 Time Profile Analysis . . . 35
2.3.1.1 Global PK-Analyses . . . 35
2.3.1.2 PK-Analyses . . . 35

**3 HV_0.5 mg/kg_chinese** **39**
3.1 Used building blocks . . . 39
3.2 Simulation Properties . . . 39
3.2.1 Model Structure . . . 39
3.2.2 Compounds . . . 39
3.2.2.1 Bevacizumab . . . 39
3.2.3 Processes . . . 40
3.2.3.1 Bevacizumab . . . 40
3.2.4 Administration . . . 40
3.2.4.1 Bevacizumab . . . 40

3.3 Charts . . . . . . 40
3.3.1 Time Profile Analysis . . . . . . 41
3.3.1.1 Global PK-Analyses . . . . . . 41
3.3.1.2 PK-Analyses . . . . . . 41

**4 Sim_Patient_1mg/kg 45**
4.1 Used building blocks . . . . . . 45
4.2 Simulation Properties . . . . . . 45
4.2.1 Model Structure . . . . . . 45
4.2.2 Compounds . . . . . . 45
4.2.2.1 Bevacizumab . . . . . . 45
4.2.3 Processes . . . . . . 46
4.2.3.1 Bevacizumab . . . . . . 46
4.2.4 Administration . . . . . . 46
4.2.4.1 Bevacizumab . . . . . . 46
4.3 Charts . . . . . . 47
4.3.1 Time Profile Analysis . . . . . . 47
4.3.1.1 PK-Analyses . . . . . . 47

**5 Sim_Patient_3mg/kg 49**
5.1 Used building blocks . . . . . . 49
5.2 Simulation Properties . . . . . . 49
5.2.1 Model Structure . . . . . . 49
5.2.2 Compounds . . . . . . 49
5.2.2.1 Bevacizumab . . . . . . 49
5.2.3 Processes . . . . . . 50
5.2.3.1 Bevacizumab . . . . . . 50
5.2.4 Administration . . . . . . 50
5.2.4.1 Bevacizumab . . . . . . 50
5.3 Charts . . . . . . 51
5.3.1 Time Profile Analysis . . . . . . 51
5.3.1.1 PK-Analyses . . . . . . 51

**6 Sim_Patient_10mg/kg 53**
6.1 Used building blocks . . . . . . 53
6.2 Simulation Properties . . . . . . 53
6.2.1 Model Structure . . . . . . 53
6.2.2 Compounds . . . . . . 53
6.2.2.1 Bevacizumab . . . . . . 53
6.2.3 Processes . . . . . . 54
6.2.3.1 Bevacizumab . . . . . . 54
6.2.4 Administration . . . . . . 54
6.2.4.1 Bevacizumab . . . . . . 54
6.3 Charts . . . . . . 55
6.3.1 Time Profile Analysis . . . . . . 55
6.3.1.1 PK-Analyses . . . . . . 55

**7 Sim_Patient_5mg/kg 57**
7.1 Used building blocks . . . . . . 57
7.2 Simulation Properties . . . . . . 57
7.2.1 Model Structure . . . . . . 57
7.2.2 Compounds . . . . . . 57
7.2.2.1 Bevacizumab . . . . . . 57
7.2.3 Processes . . . . . . 58
7.2.3.1 Bevacizumab . . . . . . 58
7.2.4 Administration . . . . . . 58
7.2.4.1 Bevacizumab . . . . . . 58
7.3 Charts . . . . . . 58
7.3.1 Time Profile Analysis . . . . . . 59

7.3.1.1 PK-Analyses . . . 59

**8 Sim_Patient_0.3mg/kg 61**
8.1 Used building blocks . . . 61
8.2 Simulation Properties . . . 61
8.2.1 Model Structure . . . 61
8.2.2 Compounds . . . 61
8.2.2.1 Bevacizumab . . . 61
8.2.3 Processes . . . 62
8.2.3.1 Bevacizumab . . . 62
8.2.4 Administration . . . 62
8.2.4.1 Bevacizumab . . . 62
8.3 Charts . . . 63
8.3.1 Time Profile Analysis . . . 63
8.3.1.1 PK-Analyses . . . 63

**9 Sim_patient_ped_5mg/kg 65**
9.1 Used building blocks . . . 65
9.2 Simulation Properties . . . 65
9.2.1 Model Structure . . . 65
9.2.2 Compounds . . . 65
9.2.2.1 Bevacizumab . . . 65
9.2.3 Processes . . . 66
9.2.3.1 Bevacizumab . . . 66
9.2.4 Administration . . . 66
9.2.4.1 Bevacizumab . . . 66
9.3 Charts . . . 66
9.3.1 Time Profile Analysis . . . 67
9.3.1.1 Global PK-Analyses . . . 67
9.3.1.2 PK-Analyses . . . 67

**10 Sim_patient_ped_15mg/kg 69**
10.1 Used building blocks . . . 69
10.2 Simulation Properties . . . 69
10.2.1 Model Structure . . . 69
10.2.2 Compounds . . . 69
10.2.2.1 Bevacizumab . . . 69
10.2.3 Processes . . . 70
10.2.3.1 Bevacizumab . . . 70
10.2.4 Administration . . . 70
10.2.4.1 Bevacizumab . . . 70
10.3 Charts . . . 70
10.3.1 Time Profile Analysis . . . 71
10.3.1.1 Global PK-Analyses . . . 71
10.3.1.2 PK-Analyses . . . 71

## III Observed Data 73

**1 Beva_Demarchi2021_HV_1mpk..Human.iv perfusion.1.Bevacizumab_human_sim 74**

**2 Beva_Hetema2017_HV_1mpk..Human.iv perfusion.1.Bevacizumab_human_sim 75**

**3 Beva_Hummel2022_HV_1mpk..Human.iv perfusion.1.Bevacizumab_human_sim 76**

**4 Beva_wu2019_HV_1mpk..Human.iv perfusion.1.Bevacizumab_human_sim.1 78**

**5 Beva_wu2019_HV_1mpk..Human.iv perfusion.1.Bevacizumab_human_sim.2 79**

**6 Beva_wu2019_HV_1mpk..Human.iv perfusion.1.Bevacizumab_human_sim.3 80**

**7 Beva_shin2020_HV_3mpk..Human.iv perfusion.3.Bevacizumab_human_sim.1 81**

**8 Beva_shin2020_HV_3mpk..Human.iv perfusion.3.Bevacizumab_human_sim.2 82**

**9 Beva_shin2020_HV_3mpk..Human.iv perfusion.3.Bevacizumab_human_sim.3 83**

**10 Beva_sinn2021_HV_3mpk..Human.iv perfusion.3.Bevacizumab_human_sim 84**

**11 Beva_li2017_HV_0.5mpk..Human.iv perfusion.0.5.Bevacizumab_human_sim.1 86**

**12 Beva_li2017_HV_0.5mpk..Human.iv perfusion.0.5.Bevacizumab_human_sim.2 87**

**13 Beva_li2017_HV_0.5mpk..Human.iv perfusion.0.5.Bevacizumab_human_sim.3 88**

**14 Beva_li2017_HV_0.5mpk..Human.iv perfusion.0.5.Bevacizumab_human_sim.4 89**

**15 Beva_li2017_HV_0.5mpk..Human.iv perfusion.0.5.Bevacizumab_human_sim.5 90**

**16 Beva_Gordon2001_patients_3mpk..Human.iv perfusion.3.Bevacizumab 91**

**17 Beva_Gordon2001_patients_0.3mpk..Human.iv perfusion.0.3.Bevacizumab 92**

**18 Beva_Gordon2001_patients_1mpk..Human.iv perfusion.1.Bevacizumab 93**

**19 Beva_Gordon2001_patients_10mpk..Human.iv perfusion.10.Bevacizumab 94**

**20 Beva_Romera2018_patients_5mpk..Human.iv perfusion.5.Bevacizumab 95**

# Part I

# Building Blocks

# Chapter 1

# Individuals

## 1.1 HV1

### 1.1.1 Biometrics

**Population Properties**

| Population Properties | Value |
|---|---|
| Species | Human |
| Population | European (ICRP, 2002) |
| Gender | Male |

**Table 1.1:** Population Properties

**Individual Parameters**

| Individual Parameters | Value | Unit |
|---|---|---|
| Age | 30.00 | year(s) |
| Weight | 73.00 | kg |
| Height | 176.00 | cm |
| BMI | 23.57 | $kg/m^2$ |

**Table 1.2:** Individual Parameters

**Calculation methods**

| Category | Calculation methods |
|---|---|
| Endothelial surface areas | Organ vascularization |
| Body surface area | Mosteller |

**Table 1.3:** Calculation methods

### 1.1.2 Anatomy & Physiology

Default

### 1.1.3 Expression

#### 1.1.3.1 Metabolizing Enzymes

None

#### 1.1.3.2 Transport Proteins

None

#### 1.1.3.3 Protein Binding Partners

VEGFA
Reference concentration: 3.00E-4 µmol/l
t1/2 (liver): 36.00 h
t1/2 (intestine): 23.00 h
Ontogeny/Variability like: Undefined
Localization: Interstitial, BloodCellsMembrane, VascMembraneTissueSide
Expression Levels

| Relative expression | Name | Value |
|---|---|---|
| VEGFA | 0.18 | 0.18 % |
| VEGFA | 0.18 | 0.18 % |

**Table 1.4:** Expression Levels

## 1.2 HV_chinese

### 1.2.1 Biometrics

**Population Properties**

| Population Properties | Value |
|---|---|
| Species | Human |
| Population | Asian (Tanaka, 1996) |
| Gender | Male |

**Table 1.5:** Population Properties

**Individual Parameters**

| Individual Parameters | Value | Unit |
|---|---|---|
| Age | 30.00 | year(s) |
| Weight | 60.03 | kg |
| Height | 169.96 | cm |
| BMI | 20.78 | $kg/m^2$ |

**Table 1.6:** Individual Parameters

**Calculation methods**

| Category | Calculation methods |
|---|---|
| Endothelial surface areas | Organ vascularization |
| Body surface area | Mosteller |

**Table 1.7:** Calculation methods

### 1.2.2 Anatomy & Physiology

Default

### 1.2.3 Expression

#### 1.2.3.1 Metabolizing Enzymes

None

#### 1.2.3.2 Transport Proteins

None

#### 1.2.3.3 Protein Binding Partners

VEGFA
Reference concentration: 3.00E-4 µmol/l
t1/2 (liver): 36.00 h
t1/2 (intestine): 23.00 h
Ontogeny/Variability like: Undefined
Localization: Interstitial, BloodCellsMembrane, VascMembraneTissueSide
Expression Levels

| Relative expression | Name | Value |
|---|---|---|
| VEGFA | 0.18 | 0.18 % |
| VEGFA | 0.18 | 0.18 % |

**Table 1.8:** Expression Levels

## 1.3 Patient1

### 1.3.1 Biometrics

**Population Properties**

| Population Properties | Value |
|---|---|
| Species | Human |
| Population | European (ICRP, 2002) |
| Gender | Male |

**Table 1.9:** Population Properties

**Individual Parameters**

| Individual Parameters | Value | Unit |
|---|---|---|
| Age | 30.00 | year(s) |
| Weight | 73.00 | kg |
| Height | 176.00 | cm |
| BMI | 23.57 | $kg/m^2$ |

**Table 1.10:** Individual Parameters

**Calculation methods**

| Category | Calculation methods |
|---|---|
| Endothelial surface areas | Organ vascularization |
| Body surface area | Mosteller |

**Table 1.11:** Calculation methods

## 1.3.2 Anatomy & Physiology

Default

## 1.3.3 Expression

### 1.3.3.1 Metabolizing Enzymes

None

### 1.3.3.2 Transport Proteins

None

### 1.3.3.3 Protein Binding Partners

VEGFA
Reference concentration: 3.00E-3 µmol/l
t1/2 (liver): 36.00 h
t1/2 (intestine): 23.00 h
Ontogeny/Variability like: Undefined
Localization: Interstitial, BloodCellsMembrane, VascMembraneTissueSide
Expression Levels

| Relative expression | Name | Value |
|---|---|---|
| VEGFA | 0.18 | 0.18 % |
| VEGFA | 0.18 | 0.18 % |

**Table 1.12:** Expression Levels

# 1.4 Patient_ped

## 1.4.1 Biometrics

### Population Properties

| Population Properties | Value |
|---|---|
| Species | Human |
| Population | European (ICRP, 2002) |
| Gender | Male |

**Table 1.13:** Population Properties

### Individual Parameters

| Individual Parameters | Value | Unit |
|---|---|---|
| Age | 13.00 | year(s) |
| Weight | 46.40 | kg |
| Height | 155.40 | cm |
| BMI | 19.21 | $kg/m^2$ |

**Table 1.14:** Individual Parameters

### Calculation methods

| Category | Calculation methods |
|---|---|
| Endothelial surface areas | Organ vascularization |
| Body surface area | Mosteller |

**Table 1.15:** Calculation methods

## 1.4.2 Anatomy & Physiology

Default

## 1.4.3 Expression

### 1.4.3.1 Metabolizing Enzymes

None

### 1.4.3.2 Transport Proteins

None

### 1.4.3.3 Protein Binding Partners

VEGFA
Reference concentration: 3.86E-3 µmol/l
t1/2 (liver): 36.00 h
t1/2 (intestine): 23.00 h

Ontogeny/Variability like: Undefined
Localization: Interstitial, BloodCellsMembrane, VascMembraneTissueSide
Expression Levels

| Relative expression | Name | Value |
|---|---|---|
| VEGFA | 0.18 | 0.18 % |
| VEGFA | 0.18 | 0.18 % |

**Table 1.16:** Expression Levels

# Chapter 2

# Compounds

## 2.1 Bevacizumab_human

### 2.1.1 Basic Physico-chemistry

Is small molecule: No

#### 2.1.1.1 Molecular weight

| Parameter | Value | Unit |
|---|---|---|
| Molecular weight | 150.00 | kDa |
| Effective molecular weight | 150000.00 | g/mol |
| I | 0 | |
| F | 0 | |
| Cl | 0 | |
| Br | 0 | |

**Table 2.1:** Molecular weight

#### 2.1.1.2 Lipophilicity

| Experiment | Lipophilicity [Log Units] |
|---|---|
| Measurement | −5.00 |

**Table 2.2:** Lipophilicity

Table 2.2 lists lipophilicity values for compound Bevacizumab_human.

#### 2.1.1.3 Fraction unbound (plasma, reference value)

| Experiment | Fraction Unbound | Species |
|---|---|---|
| Measurement | 1.00 | Human |

**Table 2.3:** Fraction unbound (plasma, reference value)

Table 2.3 lists fraction unbound values for compound Bevacizumab_human.

#### 2.1.1.4 Solubility

| Experiment | Ref-pH | Solubility at Ref-pH [mg/l] | Solubility gain per charge |
|---|---|---|---|
| Measurement | 7.00 | 9999.00 | 1000.00 |

**Table 2.4:** Solubility

Table 2.4 lists solubility values for compound Bevacizumab_human.

### 2.1.2 ADME

#### 2.1.2.1 Absorption

**Specific intestinal permeability**

| Experiment | Lipophilicity | Permeability [cm/min] |
|---|---|---|
| Calculated | Measurement | 0 |

**Table 2.5:** Specific intestinal permeability

Table 2.5 lists intestinal permeability values for compound Bevacizumab_human.

#### 2.1.2.2 Distribution

**Specific organ permeability**

| Experiment | Lipophilicity | Permeability [cm/min] |
|---|---|---|
| Calculated | Measurement | 0 |

**Table 2.6:** Specific organ permeability

Table 2.6 lists organ permeability values for compound Bevacizumab_human.

**Calculation methods**

| Category | Calculation methods |
|---|---|
| Partition coefficients | PK-Sim Standard |
| Cellular permeabilities | PK-Sim Standard |

**Table 2.7:** Calculation methods

VEGF 2-Paper 2
Process Type: Specific Binding

| Parameter | Value | Unit |
|---|---|---|
| koff | $3.10 \times 10^{-5}$ | 1/s |
| Kd | 0.06 | nmol/l |

**Table 2.8:** Parameters

VEGFA-Paper
Process Type: Specific Binding

| Parameter | Value | Unit |
|---|---|---|
| koff | $3.10 \times 10^{-5}$ | 1/s |
| Kd | 0.06 | nmol/l |

**Table 2.9:** Parameters

## 2.2 Bevacizumab

### 2.2.1 Basic Physico-chemistry

Is small molecule: No

#### 2.2.1.1 Molecular weight

| Parameter | Value | Unit |
|---|---|---|
| Molecular weight | 150.00 | kDa |
| Effective molecular weight | 150000.00 | g/mol |
| I | 0 | |
| F | 0 | |
| Cl | 0 | |
| Br | 0 | |

**Table 2.10:** Molecular weight

#### 2.2.1.2 Lipophilicity

| Experiment | Lipophilicity [Log Units] |
|---|---|
| Measurement | −5.00 |

**Table 2.11:** Lipophilicity

Table 2.11 lists lipophilicity values for compound Bevacizumab.

#### 2.2.1.3 Fraction unbound (plasma, reference value)

| Experiment | Fraction Unbound | Species |
|---|---|---|
| Measurement | 1.00 | Human |

**Table 2.12:** Fraction unbound (plasma, reference value)

Table 2.12 lists fraction unbound values for compound Bevacizumab.

#### 2.2.1.4 Solubility

| Experiment | Ref-pH | Solubility at Ref-pH [mg/l] | Solubility gain per charge |
|---|---|---|---|
| Measurement | 7.00 | 9999.00 | 1000.00 |

**Table 2.13:** Solubility

Table 2.13 lists solubility values for compound Bevacizumab.

### 2.2.2 ADME

#### 2.2.2.1 Absorption

**Specific intestinal permeability**

| Experiment | Lipophilicity | Permeability [cm/min] |
|---|---|---|
| Calculated | Measurement | 0 |

**Table 2.14:** Specific intestinal permeability

Table 2.14 lists intestinal permeability values for compound Bevacizumab.

#### 2.2.2.2 Distribution

**Specific organ permeability**

| Experiment | Lipophilicity | Permeability [cm/min] |
|---|---|---|
| Calculated | Measurement | 0 |

**Table 2.15:** Specific organ permeability

Table 2.15 lists organ permeability values for compound Bevacizumab.

**Calculation methods**

| Category | Calculation methods |
|---|---|
| Partition coefficients | PK-Sim Standard |
| Cellular permeabilities | PK-Sim Standard |

**Table 2.16:** Calculation methods

VEGF 2-Paper 2
Process Type: Specific Binding

| Parameter | Value | Unit |
|---|---|---|
| koff | $3.10 \times 10^{-5}$ | 1/s |
| Kd | 0.06 | nmol/l |

**Table 2.17:** Parameters

VEGFA-Paper
Process Type: Specific Binding

| Parameter | Value | Unit |
|---|---|---|
| koff | $3.10 \times 10^{-5}$ | 1/s |
| Kd | 0.06 | nmol/l |

**Table 2.18:** Parameters

# Chapter 3

# Protocols

## 3.1 Perfusion 90 min_1mg/kg

| Property | Value |
|---|---|
| Process Type | Simple protocol |
| Administration type | Intravenous Infusion |
| Dosing interval | Single |

**Table 3.1**

| Parameter | Value | Unit |
|---|---|---|
| Dose | 1.00 | mg/kg |
| Infusion time | 90.00 | min |

**Table 3.2:** Parameters

## 3.2 Perfusion 90 min_3mg/kg

| Property | Value |
|---|---|
| Process Type | Simple protocol |
| Administration type | Intravenous Infusion |
| Dosing interval | Single |

**Table 3.3**

| Parameter | Value | Unit |
|---|---|---|
| Dose | 3.00 | mg/kg |
| Infusion time | 90.00 | min |

**Table 3.4:** Parameters

## 3.3 Perfusion 90 min_0.5mg/kg

| Property | Value |
|---|---|
| Process Type | Simple protocol |
| Administration type | Intravenous Infusion |
| Dosing interval | Single |

**Table 3.5**

| Parameter | Value | Unit |
|---|---|---|
| Dose | 0.50 | mg/kg |
| Infusion time | 90.00 | min |

**Table 3.6:** Parameters

## 3.4 Infusion 0.3mg/kg human

**Schema 1**

- Start time: 0 day(s)
- Number of Repetitions: 1.00
- Time Between Repetitions: 0 h
- **Dosing**
  - Start time: 0 h
  - Dose: 0.30 mg/kg
  - Administration type: Intravenous Infusion
  - **Parameters**
    - Dose: 0.30 mg/kg
    - Infusion time: 90.00 min

**Schema 2**

- Start time: 28.00 day(s)
- Number of Repetitions: 1.00
- Time Between Repetitions: 0 h
- **Dosing**
  - Start time: 0 h
  - Dose: 0.30 mg/kg
  - Administration type: Intravenous Infusion
  - **Parameters**
    - Dose: 0.30 mg/kg
    - Infusion time: 60.00 min

**Schema 3**

- Start time: 35.00 day(s)
- Number of Repetitions: 1.00

- Time Between Repetitions: 0 h
- **Dosing**
  - Start time: 0 h
  - Dose: 0.30 mg/kg
  - Administration type: Intravenous Infusion
  - **Parameters**
    * Dose: 0.30 mg/kg
    * Infusion time: 60.00 min

**Schema 4**

- Start time: 42.00 day(s)
- Number of Repetitions: 1.00
- Time Between Repetitions: 0 h
- **Dosing**
  - Start time: 0 h
  - Dose: 0.30 mg/kg
  - Administration type: Intravenous Infusion
  - **Parameters**
    * Dose: 0.30 mg/kg
    * Infusion time: 60.00 min

## 3.5 Infusion 10mg/kg human

**Schema 1**

- Start time: 0 h
- Number of Repetitions: 1.00
- Time Between Repetitions: 0 h
- **Dosing**
  - Start time: 0 h
  - Dose: 10.00 mg/kg
  - Administration type: Intravenous Infusion
  - **Parameters**
    * Dose: 10.00 mg/kg
    * Infusion time: 90.00 min

**Schema 2**

- Start time: 28.00 day(s)
- Number of Repetitions: 1.00
- Time Between Repetitions: 0 h
- **Dosing**
  - Start time: 0 h
  - Dose: 10.00 mg/kg
  - Administration type: Intravenous Infusion

- Parameters
  - Dose: 10.00 mg/kg
  - Infusion time: 60.00 min

**Schema 3**

- Start time: 35.00 day(s)
- Number of Repetitions: 1.00
- Time Between Repetitions: 0 h
- **Dosing**
  - Start time: 0 h
  - Dose: 10.00 mg/kg
  - Administration type: Intravenous Infusion
  - **Parameters**
    - Dose: 10.00 mg/kg
    - Infusion time: 60.00 min

**Schema 4**

- Start time: 42.00 day(s)
- Number of Repetitions: 1.00
- Time Between Repetitions: 0 h
- **Dosing**
  - Start time: 0 h
  - Dose: 10.00 mg/kg
  - Administration type: Intravenous Infusion
  - **Parameters**
    - Dose: 10.00 mg/kg
    - Infusion time: 60.00 min

## 3.6 Infusion 1mg/kg human

**Schema 1**

- Start time: 0 day(s)
- Number of Repetitions: 1.00
- Time Between Repetitions: 90.00 min
- **Dosing**
  - Start time: 0 h
  - Dose: 1.00 mg/kg
  - Administration type: Intravenous Infusion
  - **Parameters**
    - Dose: 1.00 mg/kg
    - Infusion time: 90.00 min

**Schema 3**

- Start time: 28.00 day(s)

- Number of Repetitions: 1.00
- Time Between Repetitions: 90.00 min
- **Dosing**
  - Start time: 0 h
  - Dose: 1.00 mg/kg
  - Administration type: Intravenous Infusion
  - **Parameters**
    - Dose: 1.00 mg/kg
    - Infusion time: 60.00 min

**Schema 4**

- Start time: 35.00 day(s)
- Number of Repetitions: 1.00
- Time Between Repetitions: 90.00 min
- **Dosing**
  - Start time: 0 h
  - Dose: 1.00 mg/kg
  - Administration type: Intravenous Infusion
  - **Parameters**
    - Dose: 1.00 mg/kg
    - Infusion time: 60.00 min

**Schema 5**

- Start time: 42.00 day(s)
- Number of Repetitions: 1.00
- Time Between Repetitions: 90.00 min
- **Dosing**
  - Start time: 0 h
  - Dose: 1.00 mg/kg
  - Administration type: Intravenous Infusion
  - **Parameters**
    - Dose: 1.00 mg/kg
    - Infusion time: 60.00 min

## 3.7 Infusion 3mg/kg human

**Schema 1**

- Start time: 0 day(s)
- Number of Repetitions: 1.00
- Time Between Repetitions: 0 h
- **Dosing**
  - Start time: 0 h

  - Dose: 3.00 mg/kg
  - Administration type: Intravenous Infusion
  - **Parameters**
    * Dose: 3.00 mg/kg
    * Infusion time: 60.00 min

**Schema 2**

- Start time: 28.00 day(s)
- Number of Repetitions: 1.00
- Time Between Repetitions: 0 h
- **Dosing**
  - Start time: 0 h
  - Dose: 3.00 mg/kg
  - Administration type: Intravenous Infusion
  - **Parameters**
    * Dose: 3.00 mg/kg
    * Infusion time: 60.00 min

**Schema 3**

- Start time: 35.00 day(s)
- Number of Repetitions: 1.00
- Time Between Repetitions: 0 h
- **Dosing**
  - Start time: 0 h
  - Dose: 3.00 mg/kg
  - Administration type: Intravenous Infusion
  - **Parameters**
    * Dose: 3.00 mg/kg
    * Infusion time: 60.00 min

**Schema 4**

- Start time: 42.00 day(s)
- Number of Repetitions: 1.00
- Time Between Repetitions: 0 h
- **Dosing**
  - Start time: 0 h
  - Dose: 3.00 mg/kg
  - Administration type: Intravenous Infusion
  - **Parameters**
    * Dose: 3.00 mg/kg
    * Infusion time: 60.00 min

## 3.8 5mpk_every2weeks

**Schema 1**

- Start time: 0 h
- Number of Repetitions: 7.00
- Time Between Repetitions: 14.00 day(s)
- **Dosing**
  - Start time: 0 h
  - Dose: 5.00 mg/kg
  - Administration type: Intravenous Infusion
  - **Parameters**
    * Dose: 5.00 mg/kg
    * Infusion time: 60.00 min

## 3.9 Infusion_5mg/kg

| Property | Value |
|---|---|
| Process Type | Simple protocol |
| Administration type | Intravenous Infusion |
| Dosing interval | Single |

**Table 3.7**

| Parameter | Value | Unit |
|---|---|---|
| Dose | 5.00 | mg/kg |
| Infusion time | 60.00 | min |

**Table 3.8:** Parameters

## 3.10 Infusion_15mg/kg

| Property | Value |
|---|---|
| Process Type | Simple protocol |
| Administration type | Intravenous Infusion |
| Dosing interval | Single |

**Table 3.9**

| Parameter | Value | Unit |
|---|---|---|
| Dose | 15.00 | mg/kg |
| Infusion time | 60.00 | min |

**Table 3.10:** Parameters

# Part II

# Simulations

# Chapter 1

# HV_1mg/kg

## 1.1 Used building blocks

| Building Block | Name |
|---|---|
| Individual | HV1 (see section 1.1 in Part I) |
| Compound | Bevacizumab (see section 2.2 in Part I) |
| Protocol | Perfusion 90 min_1mg/kg (see section 3.1 in Part I) |

**Table 1.1:** Building Block

## 1.2 Simulation Properties

### 1.2.1 Model Structure

**Allow aging**
No

**Calculation methods**

| Category | Calculation methods |
|---|---|
| Endothelial surface areas | Organ vascularization |
| Body surface area | Mosteller |

**Table 1.2:** Calculation methods

### 1.2.2 Compounds

#### 1.2.2.1 Bevacizumab

**Compound Configuration**

| Parameter | Alternative in compound | Value | Unit |
|---|---|---|---|
| Solubility | Measurement | 9999.00 | mg/l |
| Lipophilicity | Measurement | −5.00 | Log Units |
| Fraction unbound (plasma, reference value) | Measurement | 1.00 | |
| *continued on next page* | | | |

| *continued from previous page* | | | |
|---|---|---|---|
| **Parameter** | **Alternative in compound** | **Value** | **Unit** |
| Specific organ permeability | Calculated | 0 | cm/min |
| Specific intestinal permeability | Calculated | 0 | cm/min |

**Table 1.3:** Compound Configuration

**Calculation methods**

| Category | Calculation methods |
|---|---|
| Partition coefficients | PK-Sim Standard |
| Cellular permeabilities | PK-Sim Standard |

**Table 1.4:** Calculation methods

### 1.2.3 Processes

#### 1.2.3.1 Bevacizumab

**Specific Binding**
Mapping VEGFA with VEGFA-Paper

### 1.2.4 Administration

#### 1.2.4.1 Bevacizumab

**Simple protocol**
Intravenous Infusion
Dosing interval: Single
Dose: 1.00 mg/kg
Infusion time: 90.00 min

## 1.3 Charts

### 1.3.1 Time Profile Analysis

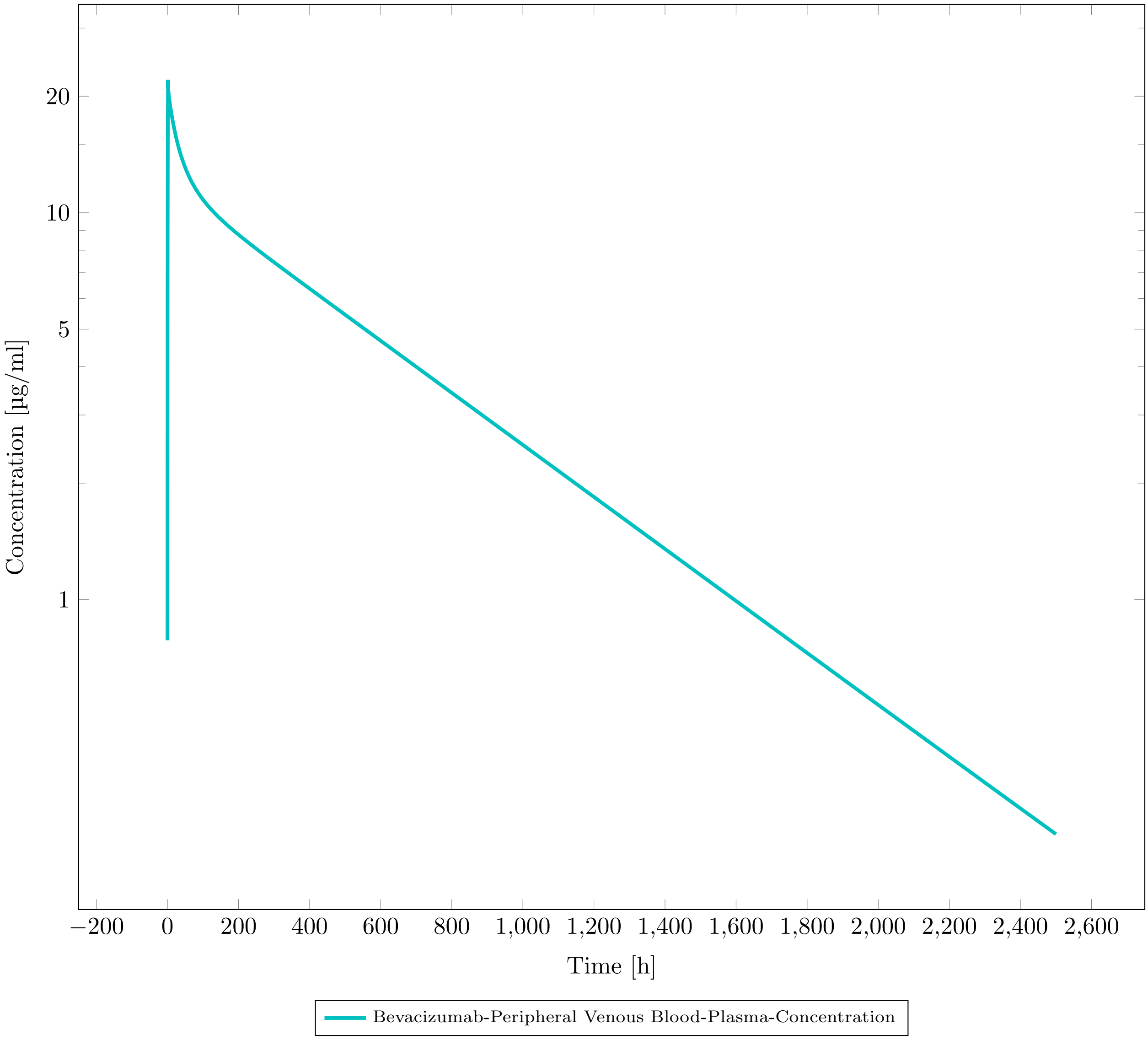


**Figure 1.1**

#### 1.3.1.1 Global PK-Analyses

| Parameter | Compound | Value | Unit |
|---|---|---|---|
| Vss (plasma) | Bevacizumab | 78.17 | ml/kg |
| Vd (plasma) | Bevacizumab | 82.14 | ml/kg |
| Vss (phys-chem) | Bevacizumab | 623.05 | ml/kg |
| Total plasma clearance | Bevacizumab | $2.09 \times 10^{-3}$ | ml/min/kg |

**Table 1.5:** Global PK-Analyses

#### 1.3.1.2 PK-Analyses

**Bevacizumab-Peripheral Venous Blood-Plasma-Concentration**

| Parameter | Value | Unit |
|---|---|---|
| C_max | 0.15 | µmol/l |
| C_max_norm | $2.20 \times 10^{7}$ | mg/l |
| t_max | 1.50 | h |
| C_tEnd | $1.65 \times 10^{-3}$ | µmol/l |
| AUC_tEnd | 3117.62 | µmol*min/l |
| AUC_tEnd_norm | $4.68 \times 10^{14}$ | µg*min/l |
| AUC_inf | 3182.35 | µmol*min/l |
| AUC_inf_norm | $4.77 \times 10^{14}$ | µg*min/l |
| MRT | 621.93 | h |
| Half-Life | 452.95 | h |
| % AUC (tlast-∞) | 0.02 | |
| Total body clearance/F | $2.09 \times 10^{-3}$ | ml/min/kg |
| Vss (plasma)/F | 78.17 | ml/kg |
| Vd (plasma)/F | 82.14 | ml/kg |

**Table 1.6:** PK-Analyses for Bevacizumab-Peripheral Venous Blood-Plasma-Concentration

**Beva_Demarchi2021_HV_1mpk..Human.iv perfusion.1.Bevacizumab_human_sim-Bevacizumab_human_-sim-Measurement**

| Parameter | Value | Unit |
|---|---|---|
| C_max | 0.15 | µmol/l |
| C_max_norm | NaN | mg/l |
| t_max | 4.86 | h |
| C_tEnd | $7.71 \times 10^{-5}$ | µmol/l |
| AUC_tEnd | 2942.18 | µmol*min/l |
| AUC_tEnd_norm | NaN | µg*min/l |
| AUC_inf | 2942.77 | µmol*min/l |
| AUC_inf_norm | NaN | µg*min/l |
| MRT | 448.08 | h |
| Half-Life | 89.57 | h |
| % AUC (tlast-∞) | $2.03 \times 10^{-4}$ | |
| Total body clearance/F | NaN | ml/min/kg |
| Vss (plasma)/F | NaN | ml/kg |
| Vd (plasma)/F | NaN | ml/kg |

**Table 1.7:** PK-Analyses for Beva_Demarchi2021_HV_1mpk..Human.iv perfusion.1.Bevacizumab_human_sim-Bevacizumab_human_sim-Measurement

**Beva_Hetema2017_HV_1mpk..Human.iv perfusion.1.Bevacizumab_human_sim-Bevacizumab_human_-sim-Measurement**

| Parameter | Value | Unit |
|---|---|---|
| C_max | 0.16 | µmol/l |
| C_max_norm | NaN | mg/l |
| t_max | 0.64 | h |
| C_tEnd | $8.38 \times 10^{-3}$ | µmol/l |
| AUC_tEnd | 2638.13 | µmol*min/l |
| AUC_tEnd_norm | NaN | µg*min/l |
| AUC_inf | NaN | µmol*min/l |
| AUC_inf_norm | NaN | µg*min/l |
| MRT | NaN | h |
| Half-Life | Infinity | h |
| % AUC (tlast-∞) | NaN | |
| Total body clearance/F | NaN | ml/min/kg |
| Vss (plasma)/F | NaN | ml/kg |
| Vd (plasma)/F | NaN | ml/kg |

**Table 1.8:** PK-Analyses for Beva_Hetema2017_HV_1mpk..Human.iv perfusion.1.Bevacizumab_human_sim-Bevacizumab_human_sim-Measurement

#### Beva_Hummel2022_HV_1mpk..Human.iv perfusion.1.Bevacizumab_human_sim-Bevacizumab_human_-sim-Measurement

| Parameter | Value | Unit |
|---|---|---|
| C_max | 0.17 | µmol/l |
| C_max_norm | NaN | mg/l |
| t_max | 1.50 | h |
| C_tEnd | $6.47 \times 10^{-4}$ | µmol/l |
| AUC_tEnd | 3230.16 | µmol*min/l |
| AUC_tEnd_norm | NaN | µg*min/l |
| AUC_inf | 3241.46 | µmol*min/l |
| AUC_inf_norm | NaN | µg*min/l |
| MRT | 476.41 | h |
| Half-Life | 201.77 | h |
| % AUC (tlast-∞) | $3.49 \times 10^{-3}$ | |
| Total body clearance/F | NaN | ml/min/kg |
| Vss (plasma)/F | NaN | ml/kg |
| Vd (plasma)/F | NaN | ml/kg |

**Table 1.9:** PK-Analyses for Beva_Hummel2022_HV_1mpk..Human.iv perfusion.1.Bevacizumab_human_sim-Bevacizumab_human_sim-Measurement

#### Beva_wu2019_HV_1mpk..Human.iv perfusion.1.Bevacizumab_human_sim.1-Bevacizumab_human_-sim-Measurement

| Parameter | Value | Unit |
|---|---|---|
| C_max | 0.17 | µmol/l |

*continued on next page*

| *continued from previous page* | | |
|---|---|---|
| **Parameter** | **Value** | **Unit** |
| C_max_norm | NaN | mg/l |
| t_max | 1.50 | h |
| C_tEnd | $1.18 \times 10^{-3}$ | µmol/l |
| AUC_tEnd | 2779.89 | µmol*min/l |
| AUC_tEnd_norm | NaN | µg*min/l |
| AUC_inf | 2821.02 | µmol*min/l |
| AUC_inf_norm | NaN | µg*min/l |
| MRT | 532.48 | h |
| Half-Life | 404.25 | h |
| % AUC (tlast-∞) | 0.01 | |
| Total body clearance/F | NaN | ml/min/kg |
| Vss (plasma)/F | NaN | ml/kg |
| Vd (plasma)/F | NaN | ml/kg |

**Table 1.10:** PK-Analyses for Beva_wu2019_HV_1mpk..Human.iv perfusion.1.Bevacizumab_human_sim.1-Bevacizumab_human_sim-Measurement

### Beva_wu2019_HV_1mpk..Human.iv perfusion.1.Bevacizumab_human_sim.2-Bevacizumab_human_-sim-Measurement

| Parameter | Value | Unit |
|---|---|---|
| C_max | 0.12 | µmol/l |
| C_max_norm | NaN | mg/l |
| t_max | 14.58 | h |
| C_tEnd | $2.20 \times 10^{-4}$ | µmol/l |
| AUC_tEnd | 2257.21 | µmol*min/l |
| AUC_tEnd_norm | NaN | µg*min/l |
| AUC_inf | NaN | µmol*min/l |
| AUC_inf_norm | NaN | µg*min/l |
| MRT | NaN | h |
| Half-Life | Infinity | h |
| % AUC (tlast-∞) | NaN | |
| Total body clearance/F | NaN | ml/min/kg |
| Vss (plasma)/F | NaN | ml/kg |
| Vd (plasma)/F | NaN | ml/kg |

**Table 1.11:** PK-Analyses for Beva_wu2019_HV_1mpk..Human.iv perfusion.1.Bevacizumab_human_sim.2-Bevacizumab_human_sim-Measurement

### Beva_wu2019_HV_1mpk..Human.iv perfusion.1.Bevacizumab_human_sim.3-Bevacizumab_human_-sim-Measurement

| Parameter | Value | Unit |
|---|---|---|
| C_max | 0.12 | µmol/l |
| | | *continued on next page* |

| *continued from previous page* | | |
|---|---|---|
| **Parameter** | **Value** | **Unit** |
| C_max_norm | NaN | mg/l |
| t_max | 14.58 | h |
| C_tEnd | $2.48 \times 10^{-3}$ | µmol/l |
| AUC_tEnd | 2995.12 | µmol*min/l |
| AUC_tEnd_norm | NaN | µg*min/l |
| AUC_inf | NaN | µmol*min/l |
| AUC_inf_norm | NaN | µg*min/l |
| MRT | NaN | h |
| Half-Life | Infinity | h |
| % AUC (tlast-∞) | NaN | |
| Total body clearance/F | NaN | ml/min/kg |
| Vss (plasma)/F | NaN | ml/kg |
| Vd (plasma)/F | NaN | ml/kg |

**Table 1.12:** PK-Analyses for Beva_wu2019_HV_1mpk..Human.iv perfusion.1.Bevacizumab_human_sim.3-Bevacizumab_human_sim-Measurement

# Chapter 2

# HV_3mg/kg

## 2.1 Used building blocks

| Building Block | Name |
|---|---|
| Individual | HV1 (see section 1.1 in Part I) |
| Compound | Bevacizumab (see section 2.2 in Part I) |
| Protocol | Perfusion 90 min_3mg/kg (see section 3.2 in Part I) |

**Table 2.1:** Building Block

## 2.2 Simulation Properties

### 2.2.1 Model Structure

**Allow aging**
No

**Calculation methods**

| Category | Calculation methods |
|---|---|
| Endothelial surface areas | Organ vascularization |
| Body surface area | Mosteller |

**Table 2.2:** Calculation methods

### 2.2.2 Compounds

#### 2.2.2.1 Bevacizumab

**Compound Configuration**

| Parameter | Alternative in compound | Value | Unit |
|---|---|---|---|
| Solubility | Measurement | 9999.00 | mg/l |
| Lipophilicity | Measurement | −5.00 | Log Units |
| Fraction unbound (plasma, reference value) | Measurement | 1.00 | |
| *continued on next page* | | | |

| *continued from previous page* | | | |
|---|---|---|---|
| **Parameter** | **Alternative in compound** | **Value** | **Unit** |
| Specific organ permeability | Calculated | 0 | cm/min |
| Specific intestinal permeability | Calculated | 0 | cm/min |

**Table 2.3:** Compound Configuration

**Calculation methods**

| Category | Calculation methods |
|---|---|
| Partition coefficients | PK-Sim Standard |
| Cellular permeabilities | PK-Sim Standard |

**Table 2.4:** Calculation methods

### 2.2.3 Processes

#### 2.2.3.1 Bevacizumab

**Specific Binding**
Mapping VEGFA with VEGFA-Paper

### 2.2.4 Administration

#### 2.2.4.1 Bevacizumab

**Simple protocol**
Intravenous Infusion
Dosing interval: Single
Dose: 3.00 mg/kg
Infusion time: 90.00 min

## 2.3 Charts

### 2.3.1 Time Profile Analysis

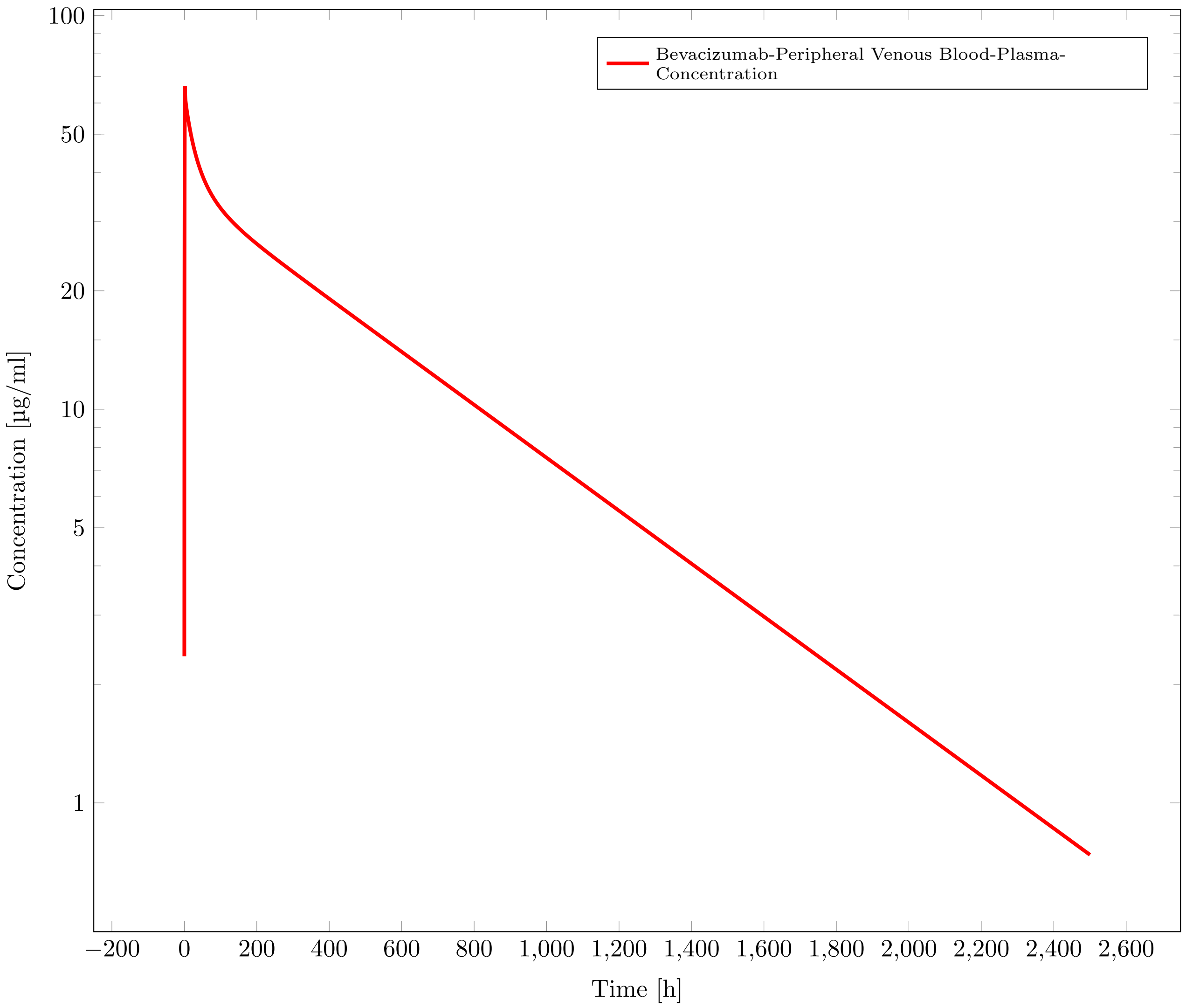


**Figure 2.1**

#### 2.3.1.1 Global PK-Analyses

| Parameter | Compound | Value | Unit |
|---|---|---|---|
| Vss (plasma) | Bevacizumab | 78.05 | ml/kg |
| Vd (plasma) | Bevacizumab | 81.35 | ml/kg |
| Vss (phys-chem) | Bevacizumab | 623.05 | ml/kg |
| Total plasma clearance | Bevacizumab | $2.10 \times 10^{-3}$ | ml/min/kg |

**Table 2.5:** Global PK-Analyses

#### 2.3.1.2 PK-Analyses

**Bevacizumab-Peripheral Venous Blood-Plasma-Concentration**

| Parameter | Value | Unit |
|---|---|---|
| C_max | 0.44 | µmol/l |
| C_max_norm | $2.21 \times 10^{7}$ | mg/l |
| t_max | 1.50 | h |
| C_tEnd | $4.92 \times 10^{-3}$ | µmol/l |
| AUC_tEnd | 9350.65 | µmol*min/l |
| AUC_tEnd_norm | $4.68 \times 10^{14}$ | µg*min/l |
| AUC_inf | 9541.60 | µmol*min/l |
| AUC_inf_norm | $4.77 \times 10^{14}$ | µg*min/l |
| MRT | 620.64 | h |
| Half-Life | 448.34 | h |
| % AUC (tlast-∞) | 0.02 | |
| Total body clearance/F | $2.10 \times 10^{-3}$ | ml/min/kg |
| Vss (plasma)/F | 78.05 | ml/kg |
| Vd (plasma)/F | 81.35 | ml/kg |

**Table 2.6:** PK-Analyses for Bevacizumab-Peripheral Venous Blood-Plasma-Concentration

**Beva_sinn2021_HV_3mpk..Human.iv perfusion.3.Bevacizumab_human_sim-Bevacizumab_human_-sim-Measurement**

| Parameter | Value | Unit |
|---|---|---|
| C_max | 0.56 | µmol/l |
| C_max_norm | NaN | mg/l |
| t_max | 0.94 | h |
| C_tEnd | $6.84 \times 10^{-3}$ | µmol/l |
| AUC_tEnd | 13496.98 | µmol*min/l |
| AUC_tEnd_norm | NaN | µg*min/l |
| AUC_inf | 13722.24 | µmol*min/l |
| AUC_inf_norm | NaN | µg*min/l |
| MRT | 569.85 | h |
| Half-Life | 380.52 | h |
| % AUC (tlast-∞) | 0.02 | |
| Total body clearance/F | NaN | ml/min/kg |
| Vss (plasma)/F | NaN | ml/kg |
| Vd (plasma)/F | NaN | ml/kg |

**Table 2.7:** PK-Analyses for Beva_sinn2021_HV_3mpk..Human.iv perfusion.3.Bevacizumab_human_sim-Bevacizumab_human_sim-Measurement

**Beva_shin2020_HV_3mpk..Human.iv perfusion.3.Bevacizumab_human_sim.3-Bevacizumab_human_-sim-Measurement**

| Parameter | Value | Unit |
|---|---|---|
| C_max | 0.39 | µmol/l |
| C_max_norm | NaN | mg/l |
| t_max | 47.73 | h |
| C_tEnd | 0.02 | µmol/l |
| AUC_tEnd | 12330.29 | µmol*min/l |
| AUC_tEnd_norm | NaN | µg*min/l |
| AUC_inf | NaN | µmol*min/l |
| AUC_inf_norm | NaN | µg*min/l |
| MRT | NaN | h |
| Half-Life | Infinity | h |
| % AUC (tlast-∞) | NaN | |
| Total body clearance/F | NaN | ml/min/kg |
| Vss (plasma)/F | NaN | ml/kg |
| Vd (plasma)/F | NaN | ml/kg |

**Table 2.8:** PK-Analyses for Beva_shin2020_HV_3mpk..Human.iv perfusion.3.Bevacizumab_human_sim.3-Bevacizumab_human_sim-Measurement

**Beva_shin2020_HV_3mpk..Human.iv perfusion.3.Bevacizumab_human_sim.2-Bevacizumab_human_-sim-Measurement**

| Parameter | Value | Unit |
|---|---|---|
| C_max | 0.27 | µmol/l |
| C_max_norm | NaN | mg/l |
| t_max | 47.73 | h |
| C_tEnd | $7.79 \times 10^{-3}$ | µmol/l |
| AUC_tEnd | 7591.91 | µmol*min/l |
| AUC_tEnd_norm | NaN | µg*min/l |
| AUC_inf | NaN | µmol*min/l |
| AUC_inf_norm | NaN | µg*min/l |
| MRT | NaN | h |
| Half-Life | Infinity | h |
| % AUC (tlast-∞) | NaN | |
| Total body clearance/F | NaN | ml/min/kg |
| Vss (plasma)/F | NaN | ml/kg |
| Vd (plasma)/F | NaN | ml/kg |

**Table 2.9:** PK-Analyses for Beva_shin2020_HV_3mpk..Human.iv perfusion.3.Bevacizumab_human_sim.2-Bevacizumab_human_sim-Measurement

**Beva_shin2020_HV_3mpk..Human.iv perfusion.3.Bevacizumab_human_sim.1-Bevacizumab_human_-sim-Measurement**

| Parameter | Value | Unit |
|---|---|---|
| C_max | 0.50 | µmol/l |

*continued on next page*

| *continued from previous page* | | |
|---|---|---|
| **Parameter** | **Value** | **Unit** |
| C_max_norm | NaN | mg/l |
| t_max | 1.50 | h |
| C_tEnd | 0.01 | µmol/l |
| AUC_tEnd | 11014.24 | µmol*min/l |
| AUC_tEnd_norm | NaN | µg*min/l |
| AUC_inf | NaN | µmol*min/l |
| AUC_inf_norm | NaN | µg*min/l |
| MRT | NaN | h |
| Half-Life | Infinity | h |
| % AUC (tlast-∞) | NaN | |
| Total body clearance/F | NaN | ml/min/kg |
| Vss (plasma)/F | NaN | ml/kg |
| Vd (plasma)/F | NaN | ml/kg |

**Table 2.10:** PK-Analyses for Beva_shin2020_HV_3mpk..Human.iv perfusion.3.Bevacizumab_human_sim.1-Bevacizumab_human_sim-Measurement

# Chapter 3

# HV_0.5 mg/kg_chinese

## 3.1 Used building blocks

| Building Block | Name |
|---|---|
| Individual | HV_chinese (see section 1.2 in Part I) |
| Compound | Bevacizumab (see section 2.2 in Part I) |
| Protocol | Perfusion 90 min_0.5mg/kg (see section 3.3 in Part I) |

**Table 3.1:** Building Block

## 3.2 Simulation Properties

### 3.2.1 Model Structure

**Allow aging**
No

**Calculation methods**

| Category | Calculation methods |
|---|---|
| Endothelial surface areas | Organ vascularization |
| Body surface area | Mosteller |

**Table 3.2:** Calculation methods

### 3.2.2 Compounds

#### 3.2.2.1 Bevacizumab

**Compound Configuration**

| Parameter | Alternative in compound | Value | Unit |
|---|---|---|---|
| Solubility | Measurement | 9999.00 | mg/l |
| Lipophilicity | Measurement | −5.00 | Log Units |
| Fraction unbound (plasma, reference value) | Measurement | 1.00 | |

*continued on next page*

| *continued from previous page* | | | |
|---|---|---|---|
| **Parameter** | **Alternative in compound** | **Value** | **Unit** |
| Specific organ permeability | Calculated | 0 | cm/min |
| Specific intestinal permeability | Calculated | 0 | cm/min |

**Table 3.3:** Compound Configuration

**Calculation methods**

| Category | Calculation methods |
|---|---|
| Partition coefficients | PK-Sim Standard |
| Cellular permeabilities | PK-Sim Standard |

**Table 3.4:** Calculation methods

### 3.2.3 Processes

#### 3.2.3.1 Bevacizumab

**Specific Binding**
Mapping VEGFA with VEGFA-Paper

### 3.2.4 Administration

#### 3.2.4.1 Bevacizumab

**Simple protocol**
Intravenous Infusion
Dosing interval: Single
Dose: 0.50 mg/kg
Infusion time: 90.00 min

## 3.3 Charts

### 3.3.1 Time Profile Analysis

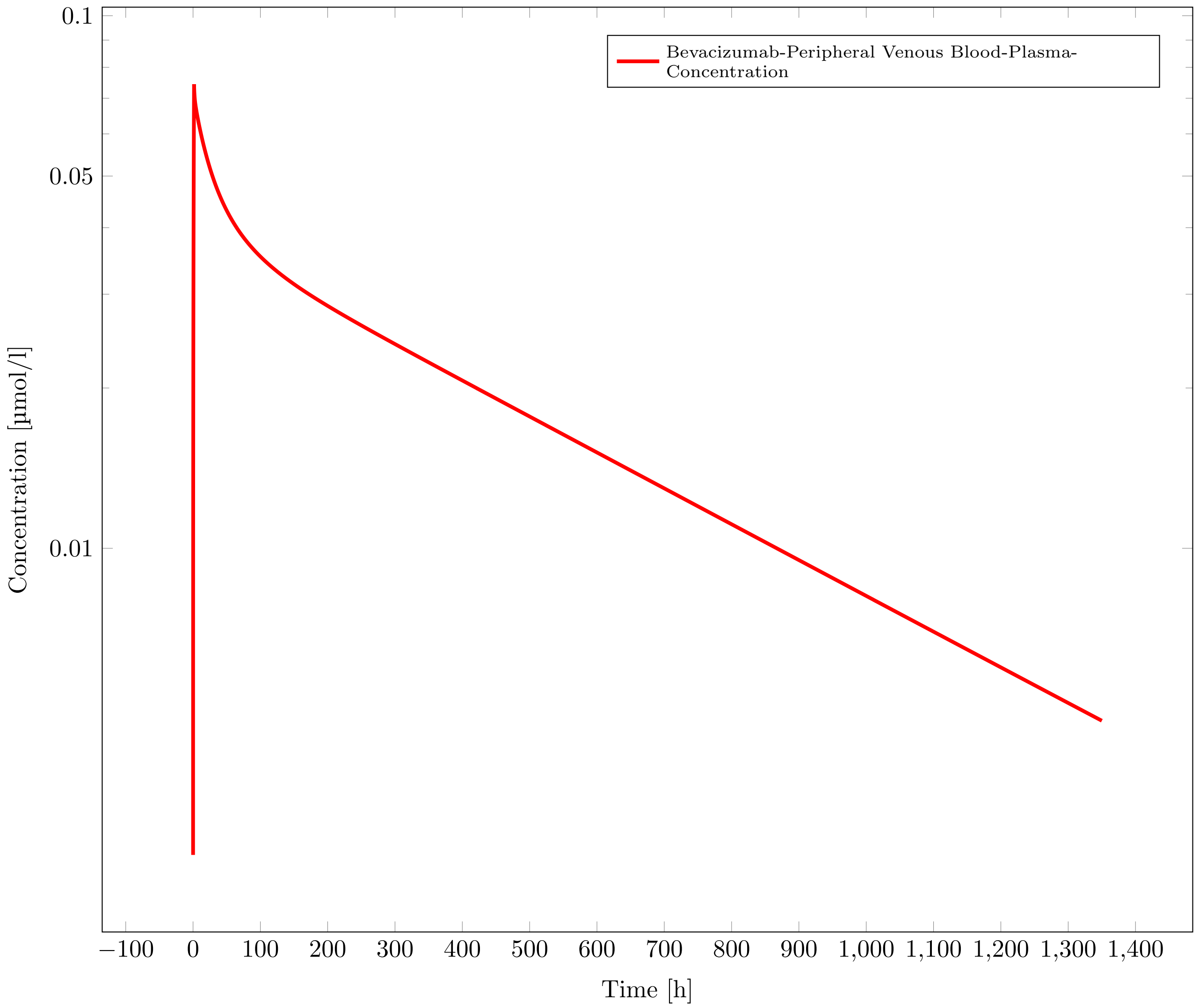


**Figure 3.1**

#### 3.3.1.1 Global PK-Analyses

| Parameter | Compound | Value | Unit |
|---|---|---|---|
| Vss (plasma) | Bevacizumab | 79.80 | ml/kg |
| Vd (plasma) | Bevacizumab | 83.77 | ml/kg |
| Vss (phys-chem) | Bevacizumab | 616.73 | ml/kg |
| Total plasma clearance | Bevacizumab | $2.14 \times 10^{-3}$ | ml/min/kg |

**Table 3.5:** Global PK-Analyses

#### 3.3.1.2 PK-Analyses

**Bevacizumab-Peripheral Venous Blood-Plasma-Concentration**

| Parameter | Value | Unit |
|---|---|---|
| C_max | 0.07 | µmol/l |
| C_max_norm | $2.23 \times 10^{7}$ | mg/l |
| t_max | 1.50 | h |
| C_tEnd | $4.74 \times 10^{-3}$ | µmol/l |
| AUC_tEnd | 1370.41 | µmol*min/l |
| AUC_tEnd_norm | $4.11 \times 10^{14}$ | µg*min/l |
| AUC_inf | 1555.93 | µmol*min/l |
| AUC_inf_norm | $4.67 \times 10^{14}$ | µg*min/l |
| MRT | 620.78 | h |
| Half-Life | 451.72 | h |
| % AUC (tlast-∞) | 0.12 | |
| Total body clearance/F | $2.14 \times 10^{-3}$ | ml/min/kg |
| Vss (plasma)/F | 79.80 | ml/kg |
| Vd (plasma)/F | 83.77 | ml/kg |

**Table 3.6:** PK-Analyses for Bevacizumab-Peripheral Venous Blood-Plasma-Concentration

**Beva_li2017_HV_0.5mpk..Human.iv perfusion.0.5.Bevacizumab_human_sim.1-Bevacizumab_human_-sim-Measurement**

| Parameter | Value | Unit |
|---|---|---|
| C_max | 0.04 | µmol/l |
| C_max_norm | NaN | mg/l |
| t_max | 2.00 | h |
| C_tEnd | $1.00 \times 10^{-3}$ | µmol/l |
| AUC_tEnd | 889.66 | µmol*min/l |
| AUC_tEnd_norm | NaN | µg*min/l |
| AUC_inf | NaN | µmol*min/l |
| AUC_inf_norm | NaN | µg*min/l |
| MRT | NaN | h |
| Half-Life | Infinity | h |
| % AUC (tlast-∞) | NaN | |
| Total body clearance/F | NaN | ml/min/kg |
| Vss (plasma)/F | NaN | ml/kg |
| Vd (plasma)/F | NaN | ml/kg |

**Table 3.7:** PK-Analyses for Beva_li2017_HV_0.5mpk..Human.iv perfusion.0.5.Bevacizumab_human_sim.1-Bevacizumab_human_sim-Measurement

**Beva_li2017_HV_0.5mpk..Human.iv perfusion.0.5.Bevacizumab_human_sim.2-Bevacizumab_human_-sim-Measurement**

| Parameter | Value | Unit |
|---|---|---|
| C_max | 0.04 | µmol/l |
| C_max_norm | NaN | mg/l |
| t_max | 2.00 | h |
| C_tEnd | $5.21 \times 10^{-4}$ | µmol/l |
| AUC_tEnd | 538.67 | µmol*min/l |
| AUC_tEnd_norm | NaN | µg*min/l |
| AUC_inf | NaN | µmol*min/l |
| AUC_inf_norm | NaN | µg*min/l |
| MRT | NaN | h |
| Half-Life | Infinity | h |
| % AUC (tlast-∞) | NaN | |
| Total body clearance/F | NaN | ml/min/kg |
| Vss (plasma)/F | NaN | ml/kg |
| Vd (plasma)/F | NaN | ml/kg |

**Table 3.8:** PK-Analyses for Beva_li2017_HV_0.5mpk..Human.iv perfusion.0.5.Bevacizumab_human_sim.2-Bevacizumab_human_sim-Measurement

### Beva_li2017_HV_0.5mpk..Human.iv perfusion.0.5.Bevacizumab_human_sim.3-Bevacizumab_human_-sim-Measurement

| Parameter | Value | Unit |
|---|---|---|
| C_max | 0.05 | µmol/l |
| C_max_norm | NaN | mg/l |
| t_max | 2.00 | h |
| C_tEnd | $7.56 \times 10^{-4}$ | µmol/l |
| AUC_tEnd | 1054.14 | µmol*min/l |
| AUC_tEnd_norm | NaN | µg*min/l |
| AUC_inf | NaN | µmol*min/l |
| AUC_inf_norm | NaN | µg*min/l |
| MRT | NaN | h |
| Half-Life | Infinity | h |
| % AUC (tlast-∞) | NaN | |
| Total body clearance/F | NaN | ml/min/kg |
| Vss (plasma)/F | NaN | ml/kg |
| Vd (plasma)/F | NaN | ml/kg |

**Table 3.9:** PK-Analyses for Beva_li2017_HV_0.5mpk..Human.iv perfusion.0.5.Bevacizumab_human_sim.3-Bevacizumab_human_sim-Measurement

### Beva_li2017_HV_0.5mpk..Human.iv perfusion.0.5.Bevacizumab_human_sim.4-Bevacizumab_human_-sim-Measurement

| Parameter | Value | Unit |
|---|---|---|
| C_max | 0.05 | µmol/l |

*continued on next page*

| *continued from previous page* | | |
|---|---|---|
| **Parameter** | **Value** | **Unit** |
| C_max_norm | NaN | mg/l |
| t_max | 2.00 | h |
| C_tEnd | $1.17 \times 10^{-3}$ | µmol/l |
| AUC_tEnd | 914.90 | µmol*min/l |
| AUC_tEnd_norm | NaN | µg*min/l |
| AUC_inf | NaN | µmol*min/l |
| AUC_inf_norm | NaN | µg*min/l |
| MRT | NaN | h |
| Half-Life | Infinity | h |
| % AUC (tlast-∞) | NaN | |
| Total body clearance/F | NaN | ml/min/kg |
| Vss (plasma)/F | NaN | ml/kg |
| Vd (plasma)/F | NaN | ml/kg |

**Table 3.10:** PK-Analyses for Beva_li2017_HV_0.5mpk..Human.iv perfusion.0.5.Bevacizumab_human_sim.4-Bevacizumab_human_sim-Measurement

**Beva_li2017_HV_0.5mpk..Human.iv perfusion.0.5.Bevacizumab_human_sim.5-Bevacizumab_human_-sim-Measurement**

| **Parameter** | **Value** | **Unit** |
|---|---|---|
| C_max | 0.05 | µmol/l |
| C_max_norm | NaN | mg/l |
| t_max | 2.00 | h |
| C_tEnd | $1.08 \times 10^{-3}$ | µmol/l |
| AUC_tEnd | 766.72 | µmol*min/l |
| AUC_tEnd_norm | NaN | µg*min/l |
| AUC_inf | NaN | µmol*min/l |
| AUC_inf_norm | NaN | µg*min/l |
| MRT | NaN | h |
| Half-Life | Infinity | h |
| % AUC (tlast-∞) | NaN | |
| Total body clearance/F | NaN | ml/min/kg |
| Vss (plasma)/F | NaN | ml/kg |
| Vd (plasma)/F | NaN | ml/kg |

**Table 3.11:** PK-Analyses for Beva_li2017_HV_0.5mpk..Human.iv perfusion.0.5.Bevacizumab_human_sim.5-Bevacizumab_human_sim-Measurement

# Chapter 4

# Sim_Patient_1mg/kg

## 4.1 Used building blocks

| Building Block | Name |
|---|---|
| Individual | Patient1 (see section 1.3 in Part I) |
| Compound | Bevacizumab (see section 2.2 in Part I) |
| Protocol | Infusion 1mg/kg human (see section 3.6 in Part I) |

**Table 4.1:** Building Block

## 4.2 Simulation Properties

### 4.2.1 Model Structure

**Allow aging**
No

**Calculation methods**

| Category | Calculation methods |
|---|---|
| Endothelial surface areas | Organ vascularization |
| Body surface area | Mosteller |

**Table 4.2:** Calculation methods

### 4.2.2 Compounds

#### 4.2.2.1 Bevacizumab

**Compound Configuration**

| Parameter | Alternative in compound | Value | Unit |
|---|---|---|---|
| Solubility | Measurement | 9999.00 | mg/l |
| Lipophilicity | Measurement | −5.00 | Log Units |
| Fraction unbound (plasma, reference value) | Measurement | 1.00 | |
| *continued on next page* | | | |

| *continued from previous page* | | | |
|---|---|---|---|
| **Parameter** | **Alternative in compound** | **Value** | **Unit** |
| Specific organ permeability | Calculated | 0 | cm/min |
| Specific intestinal permeability | Calculated | 0 | cm/min |

**Table 4.3:** Compound Configuration

**Calculation methods**

| **Category** | **Calculation methods** |
|---|---|
| Partition coefficients | PK-Sim Standard |
| Cellular permeabilities | PK-Sim Standard |

**Table 4.4:** Calculation methods

### 4.2.3 Processes

#### 4.2.3.1 Bevacizumab

**Specific Binding**
Mapping VEGFA with VEGFA-Paper

### 4.2.4 Administration

#### 4.2.4.1 Bevacizumab

**Schema Item 1**
Intravenous Infusion
Start time: 0 h
Dose: 1.00 mg/kg
Infusion time: 90.00 min

**Schema Item 1**
Intravenous Infusion
Start time: 0 h
Dose: 1.00 mg/kg
Infusion time: 60.00 min

**Schema Item 1**
Intravenous Infusion
Start time: 0 h
Dose: 1.00 mg/kg
Infusion time: 60.00 min

**Schema Item 1**
Intravenous Infusion
Start time: 0 h
Dose: 1.00 mg/kg
Infusion time: 60.00 min

## 4.3 Charts

### 4.3.1 Time Profile Analysis

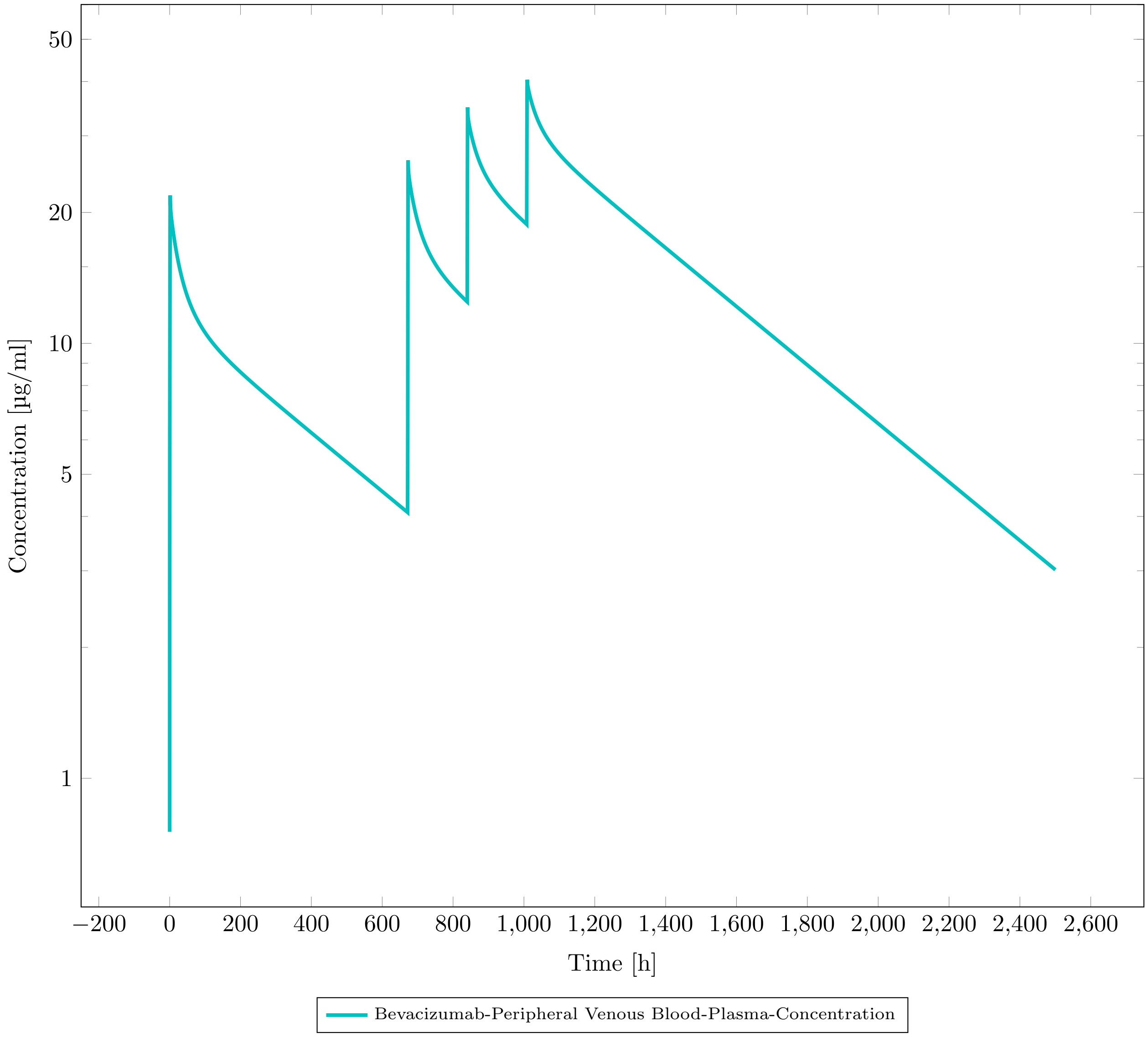


**Figure 4.1**

#### 4.3.1.1 PK-Analyses

**Bevacizumab-Peripheral Venous Blood-Plasma-Concentration**

| Parameter | Value | Unit |
|---|---|---|
| C_max | 0.27 | µmol/l |
| C_max_norm | $1.01 \times 10^7$ | mg/l |
| C_max_tD1-tD2 | 0.15 | µmol/l |
| C_max_tD1-tD2_norm | $2.19 \times 10^7$ | mg/l |
| C_max_tDlast-tEnd | 0.27 | µmol/l |
| C_max_tDLast-tEnd_norm | $4.04 \times 10^7$ | mg/l |
| t_max | 1009.40 | h |
| t_max_tD1-tD2 | 1.50 | h |

*continued on next page*

| *continued from previous page* | | |
|---|---|---|
| **Parameter** | **Value** | **Unit** |
| t_max_tDlast-tEnd | 1009.40 | h |
| C_trough_tD2 | 0.04 | µmol/l |
| C_trough_tDlast | 0.02 | µmol/l |
| AUC_tD1-tD2 | 2060.15 | µmol*min/l |
| AUC_tD1-tD2_norm | $3.09 \times 10^{14}$ | µg*min/l |
| AUC_tDlast-1_tDlast | 1556.16 | µmol*min/l |
| AUC_tDlast-1_tDlast_norm | $2.33 \times 10^{14}$ | µg*min/l |
| AUC_inf_tD1 | 0 | µmol*min/l |
| AUC_inf_tD1_norm | 0 | µg*min/l |
| AUC_inf_tDLast | 7984.57 | µmol*min/l |
| AUC_inf_tDLast_norm | $1.20 \times 10^{15}$ | µg*min/l |
| MRT | NaN | h |
| Half-Life | $-0.77$ | h |
| Half-Life_tDlast-tEnd | 448.10 | h |

**Table 4.5:** PK-Analyses for Bevacizumab-Peripheral Venous Blood-Plasma-Concentration

**Beva_Gordon2001_patients_1mpk..Human.iv perfusion.1.Bevacizumab-Bevacizumab-Measurement**

| **Parameter** | **Value** | **Unit** |
|---|---|---|
| C_max | 0.30 | µmol/l |
| C_max_norm | $1.14 \times 10^{7}$ | mg/l |
| t_max | 1006.87 | h |
| C_tEnd | 0.06 | µmol/l |
| AUC_tEnd | 10839.74 | µmol*min/l |
| AUC_tEnd_norm | $4.06 \times 10^{14}$ | µg*min/l |
| AUC_inf | NaN | µmol*min/l |
| AUC_inf_norm | NaN | µg*min/l |
| MRT | NaN | h |
| Half-Life | Infinity | h |
| % AUC (tlast-$\infty$) | NaN | |
| Total body clearance/F | NaN | ml/min/kg |
| Vss (plasma)/F | NaN | ml/kg |
| Vd (plasma)/F | NaN | ml/kg |

**Table 4.6:** PK-Analyses for Beva_Gordon2001_patients_1mpk..Human.iv perfusion.1.Bevacizumab-Bevacizumab-Measurement

# Chapter 5

# Sim_Patient_3mg/kg

## 5.1 Used building blocks

| Building Block | Name |
|---|---|
| Individual | Patient1 (see section 1.3 in Part I) |
| Compound | Bevacizumab (see section 2.2 in Part I) |
| Protocol | Infusion 3mg/kg human (see section 3.7 in Part I) |

**Table 5.1:** Building Block

## 5.2 Simulation Properties

### 5.2.1 Model Structure

**Allow aging**
No

**Calculation methods**

| Category | Calculation methods |
|---|---|
| Endothelial surface areas | Organ vascularization |
| Body surface area | Mosteller |

**Table 5.2:** Calculation methods

### 5.2.2 Compounds

#### 5.2.2.1 Bevacizumab

**Compound Configuration**

| Parameter | Alternative in compound | Value | Unit |
|---|---|---|---|
| Solubility | Measurement | 9999.00 | mg/l |
| Lipophilicity | Measurement | −5.00 | Log Units |
| Fraction unbound (plasma, reference value) | Measurement | 1.00 | |
| *continued on next page* | | | |

| *continued from previous page* | | | |
|---|---|---|---|
| **Parameter** | **Alternative in compound** | **Value** | **Unit** |
| Specific organ permeability | Calculated | 0 | cm/min |
| Specific intestinal permeability | Calculated | 0 | cm/min |

**Table 5.3:** Compound Configuration

**Calculation methods**

| **Category** | **Calculation methods** |
|---|---|
| Partition coefficients | PK-Sim Standard |
| Cellular permeabilities | PK-Sim Standard |

**Table 5.4:** Calculation methods

## 5.2.3 Processes

### 5.2.3.1 Bevacizumab

**Specific Binding**
Mapping VEGFA with VEGFA-Paper

## 5.2.4 Administration

### 5.2.4.1 Bevacizumab

**Schema Item 1**
Intravenous Infusion
Start time: 0 h
Dose: 3.00 mg/kg
Infusion time: 60.00 min

**Schema Item 1**
Intravenous Infusion
Start time: 0 h
Dose: 3.00 mg/kg
Infusion time: 60.00 min

**Schema Item 1**
Intravenous Infusion
Start time: 0 h
Dose: 3.00 mg/kg
Infusion time: 60.00 min

**Schema Item 1**
Intravenous Infusion
Start time: 0 h
Dose: 3.00 mg/kg
Infusion time: 60.00 min

## 5.3 Charts

### 5.3.1 Time Profile Analysis

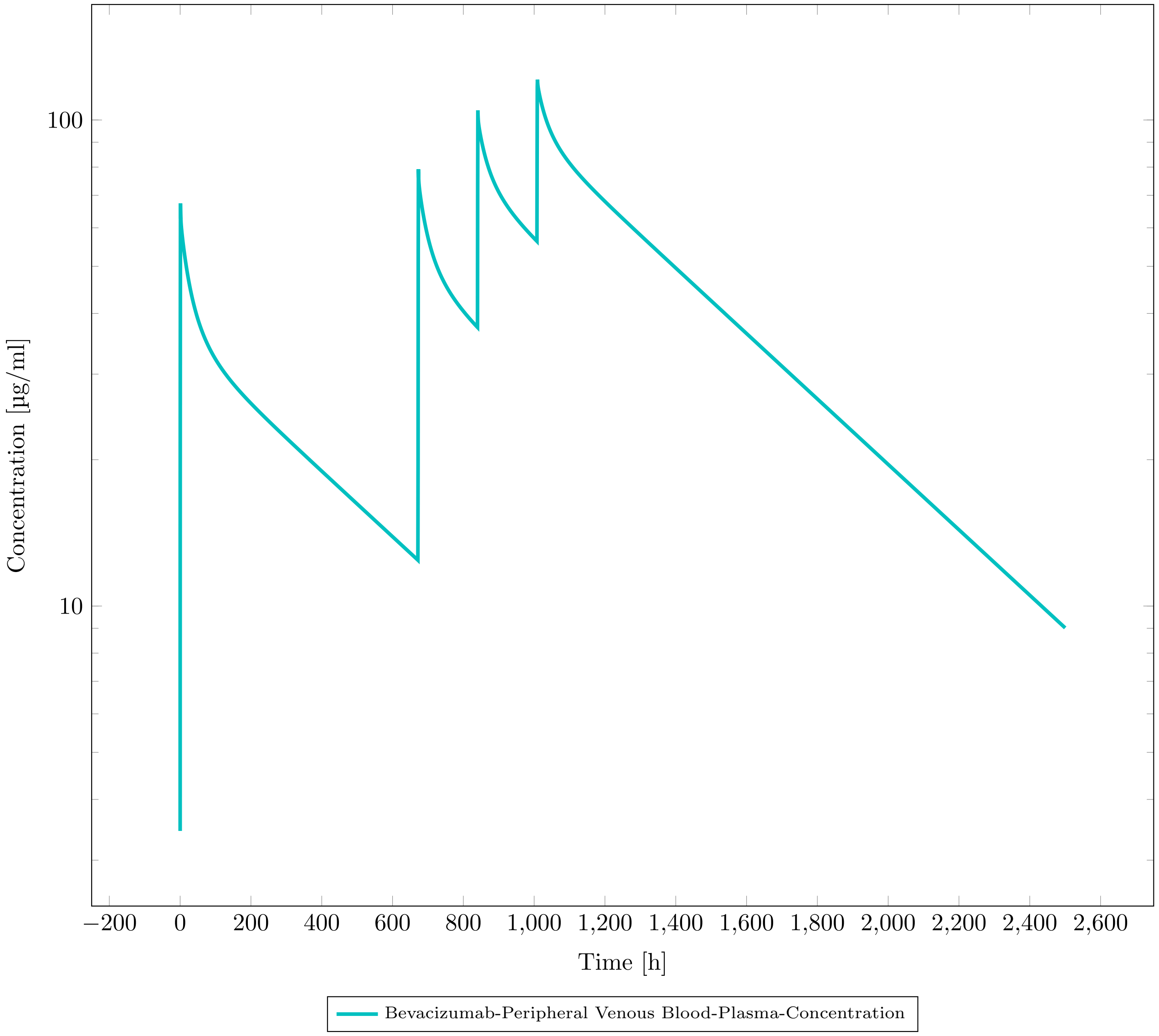


**Figure 5.1**

#### 5.3.1.1 PK-Analyses

**Bevacizumab-Peripheral Venous Blood-Plasma-Concentration**

| Parameter | Value | Unit |
|---|---|---|
| C_max | 0.81 | µmol/l |
| C_max_norm | $1.01 \times 10^7$ | mg/l |
| C_max_tD1-tD2 | 0.45 | µmol/l |
| C_max_tD1-tD2_norm | $2.25 \times 10^7$ | mg/l |
| C_max_tDlast-tEnd | 0.81 | µmol/l |
| C_max_tDLast-tEnd_norm | $4.04 \times 10^7$ | mg/l |
| t_max | 1009.40 | h |
| t_max_tD1-tD2 | 1.00 | h |

*continued on next page*

| *continued from previous page* | | |
|---|---|---|
| **Parameter** | **Value** | **Unit** |
| t_max_tDlast-tEnd | 1009.40 | h |
| C_trough_tD2 | 0.13 | µmol/l |
| C_trough_tDlast | 0.06 | µmol/l |
| AUC_tD1-tD2 | 6267.91 | µmol*min/l |
| AUC_tD1-tD2_norm | $3.13 \times 10^{14}$ | µg*min/l |
| AUC_tDlast-1_tDlast | 4667.69 | µmol*min/l |
| AUC_tDlast-1_tDlast_norm | $2.33 \times 10^{14}$ | µg*min/l |
| AUC_inf_tD1 | 0 | µmol*min/l |
| AUC_inf_tD1_norm | 0 | µg*min/l |
| AUC_inf_tDLast | 23890.55 | µmol*min/l |
| AUC_inf_tDLast_norm | $1.19 \times 10^{15}$ | µg*min/l |
| MRT | NaN | h |
| Half-Life | $-0.77$ | h |
| Half-Life_tDlast-tEnd | 447.50 | h |

**Table 5.5:** PK-Analyses for Bevacizumab-Peripheral Venous Blood-Plasma-Concentration

### Beva_Gordon2001_patients_3mpk..Human.iv perfusion.3.Bevacizumab-Bevacizumab-Measurement

| Parameter | Value | Unit |
|---|---|---|
| C_max | 1.14 | µmol/l |
| C_max_norm | $1.42 \times 10^{7}$ | mg/l |
| t_max | 1008.57 | h |
| C_tEnd | 0.14 | µmol/l |
| AUC_tEnd | 34293.29 | µmol*min/l |
| AUC_tEnd_norm | $4.29 \times 10^{14}$ | µg*min/l |
| AUC_inf | NaN | µmol*min/l |
| AUC_inf_norm | NaN | µg*min/l |
| MRT | NaN | h |
| Half-Life | Infinity | h |
| % AUC (tlast-∞) | NaN | |
| Total body clearance/F | NaN | ml/min/kg |
| Vss (plasma)/F | NaN | ml/kg |
| Vd (plasma)/F | NaN | ml/kg |

**Table 5.6:** PK-Analyses for Beva_Gordon2001_patients_3mpk..Human.iv perfusion.3.Bevacizumab-Bevacizumab-Measurement

# Chapter 6

# Sim_Patient_10mg/kg

## 6.1 Used building blocks

| Building Block | Name |
|---|---|
| Individual | Patient1 (see section 1.3 in Part I) |
| Compound | Bevacizumab (see section 2.2 in Part I) |
| Protocol | Infusion 10mg/kg human (see section 3.5 in Part I) |

**Table 6.1:** Building Block

## 6.2 Simulation Properties

### 6.2.1 Model Structure

**Allow aging**
No

**Calculation methods**

| Category | Calculation methods |
|---|---|
| Endothelial surface areas | Organ vascularization |
| Body surface area | Mosteller |

**Table 6.2:** Calculation methods

### 6.2.2 Compounds

#### 6.2.2.1 Bevacizumab

**Compound Configuration**

| Parameter | Alternative in compound | Value | Unit |
|---|---|---|---|
| Solubility | Measurement | 9999.00 | mg/l |
| Lipophilicity | Measurement | −5.00 | Log Units |
| Fraction unbound (plasma, reference value) | Measurement | 1.00 | |
| *continued on next page* | | | |

| *continued from previous page* | | | |
|---|---|---|---|
| **Parameter** | **Alternative in compound** | **Value** | **Unit** |
| Specific organ permeability | Calculated | 0 | cm/min |
| Specific intestinal permeability | Calculated | 0 | cm/min |

**Table 6.3:** Compound Configuration

#### Calculation methods

| Category | Calculation methods |
|---|---|
| Partition coefficients | PK-Sim Standard |
| Cellular permeabilities | PK-Sim Standard |

**Table 6.4:** Calculation methods

## 6.2.3 Processes

### 6.2.3.1 Bevacizumab

**Specific Binding**
Mapping VEGFA with VEGFA-Paper

## 6.2.4 Administration

### 6.2.4.1 Bevacizumab

**Schema Item 1**
Intravenous Infusion
Start time: 0 h
Dose: 10.00 mg/kg
Infusion time: 90.00 min

**Schema Item 1**
Intravenous Infusion
Start time: 0 h
Dose: 10.00 mg/kg
Infusion time: 60.00 min

**Schema Item 1**
Intravenous Infusion
Start time: 0 h
Dose: 10.00 mg/kg
Infusion time: 60.00 min

**Schema Item 1**
Intravenous Infusion
Start time: 0 h
Dose: 10.00 mg/kg
Infusion time: 60.00 min

## 6.3 Charts

### 6.3.1 Time Profile Analysis

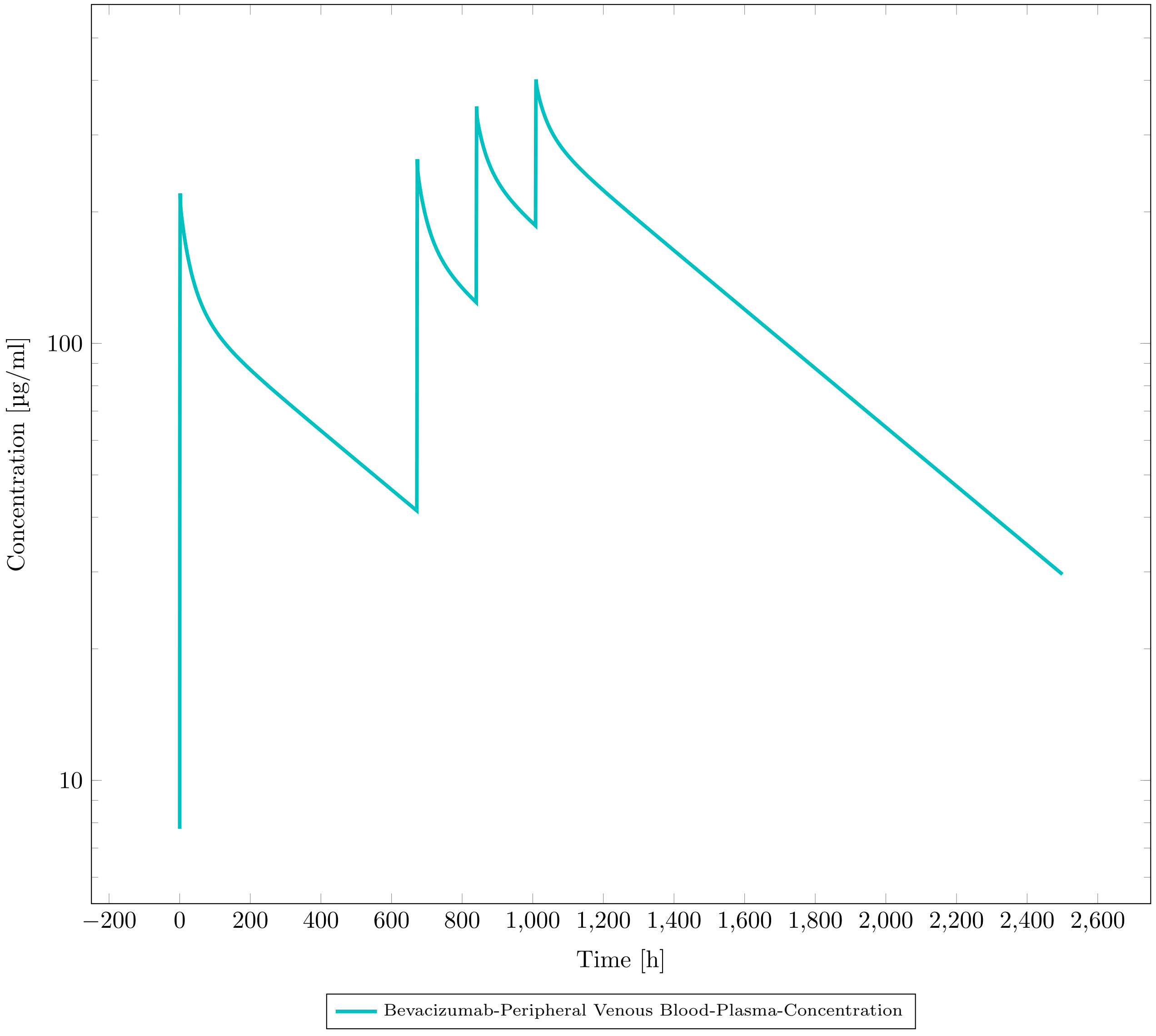


**Figure 6.1**

#### 6.3.1.1 PK-Analyses

**Bevacizumab-Peripheral Venous Blood-Plasma-Concentration**

| Parameter | Value | Unit |
|---|---|---|
| C_max | 2.68 | µmol/l |
| C_max_norm | $1.00 \times 10^7$ | mg/l |
| C_max_tD1-tD2 | 1.47 | µmol/l |
| C_max_tD1-tD2_norm | $2.20 \times 10^7$ | mg/l |
| C_max_tDlast-tEnd | 2.68 | µmol/l |
| C_max_tDLast-tEnd_norm | $4.02 \times 10^7$ | mg/l |
| t_max | 1009.40 | h |
| t_max_tD1-tD2 | 1.50 | h |

*continued on next page*

| *continued from previous page* | | |
|---|---|---|
| **Parameter** | **Value** | **Unit** |
| t_max_tDlast-tEnd | 1009.40 | h |
| C_trough_tD2 | 0.43 | µmol/l |
| C_trough_tDlast | 0.20 | µmol/l |
| AUC_tD1-tD2 | 20891.18 | µmol*min/l |
| AUC_tD1-tD2_norm | $3.13 \times 10^{14}$ | µg*min/l |
| AUC_tDlast-1_tDlast | 15454.22 | µmol*min/l |
| AUC_tDlast-1_tDlast_norm | $2.32 \times 10^{14}$ | µg*min/l |
| AUC_inf_tD1 | 0 | µmol*min/l |
| AUC_inf_tD1_norm | 0 | µg*min/l |
| AUC_inf_tDLast | 78648.48 | µmol*min/l |
| AUC_inf_tDLast_norm | $1.18 \times 10^{15}$ | µg*min/l |
| MRT | NaN | h |
| Half-Life | −0.77 | h |
| Half-Life_tDlast-tEnd | 447.26 | h |

**Table 6.5:** PK-Analyses for Bevacizumab-Peripheral Venous Blood-Plasma-Concentration

**Beva_Gordon2001_patients_10mpk..Human.iv perfusion.10.Bevacizumab-Bevacizumab-Measurement**

| Parameter | Value | Unit |
|---|---|---|
| C_max | 2.85 | µmol/l |
| C_max_norm | $1.07 \times 10^{7}$ | mg/l |
| t_max | 1006.87 | h |
| C_tEnd | 0.61 | µmol/l |
| AUC_tEnd | 111357.59 | µmol*min/l |
| AUC_tEnd_norm | $4.18 \times 10^{14}$ | µg*min/l |
| AUC_inf | NaN | µmol*min/l |
| AUC_inf_norm | NaN | µg*min/l |
| MRT | NaN | h |
| Half-Life | Infinity | h |
| % AUC (tlast-∞) | NaN | |
| Total body clearance/F | NaN | ml/min/kg |
| Vss (plasma)/F | NaN | ml/kg |
| Vd (plasma)/F | NaN | ml/kg |

**Table 6.6:** PK-Analyses for Beva_Gordon2001_patients_10mpk..Human.iv perfusion.10.Bevacizumab-Bevacizumab-Measurement

# Chapter 7

# Sim_Patient_5mg/kg

## 7.1 Used building blocks

| Building Block | Name |
|---|---|
| Individual | Patient1 (see section 1.3 in Part I) |
| Compound | Bevacizumab (see section 2.2 in Part I) |
| Protocol | 5mpk_every2weeks (see section 3.8 in Part I) |

**Table 7.1:** Building Block

## 7.2 Simulation Properties

### 7.2.1 Model Structure

**Allow aging**
No

**Calculation methods**

| Category | Calculation methods |
|---|---|
| Endothelial surface areas | Organ vascularization |
| Body surface area | Mosteller |

**Table 7.2:** Calculation methods

### 7.2.2 Compounds

#### 7.2.2.1 Bevacizumab

**Compound Configuration**

| Parameter | Alternative in compound | Value | Unit |
|---|---|---|---|
| Solubility | Measurement | 9999.00 | mg/l |
| Lipophilicity | Measurement | −5.00 | Log Units |
| Fraction unbound (plasma, reference value) | Measurement | 1.00 | |
| *continued on next page* | | | |

| *continued from previous page* | | | |
|---|---|---|---|
| **Parameter** | **Alternative in compound** | **Value** | **Unit** |
| Specific organ permeability | Calculated | 0 | cm/min |
| Specific intestinal permeability | Calculated | 0 | cm/min |

**Table 7.3:** Compound Configuration

**Calculation methods**

| Category | Calculation methods |
|---|---|
| Partition coefficients | PK-Sim Standard |
| Cellular permeabilities | PK-Sim Standard |

**Table 7.4:** Calculation methods

### 7.2.3 Processes

#### 7.2.3.1 Bevacizumab

**Specific Binding**
Mapping VEGFA with VEGFA-Paper

### 7.2.4 Administration

#### 7.2.4.1 Bevacizumab

**Schema Item 1**
Intravenous Infusion
Start time: 0 h
Dose: 5.00 mg/kg
Infusion time: 60.00 min

## 7.3 Charts

### 7.3.1 Time Profile Analysis

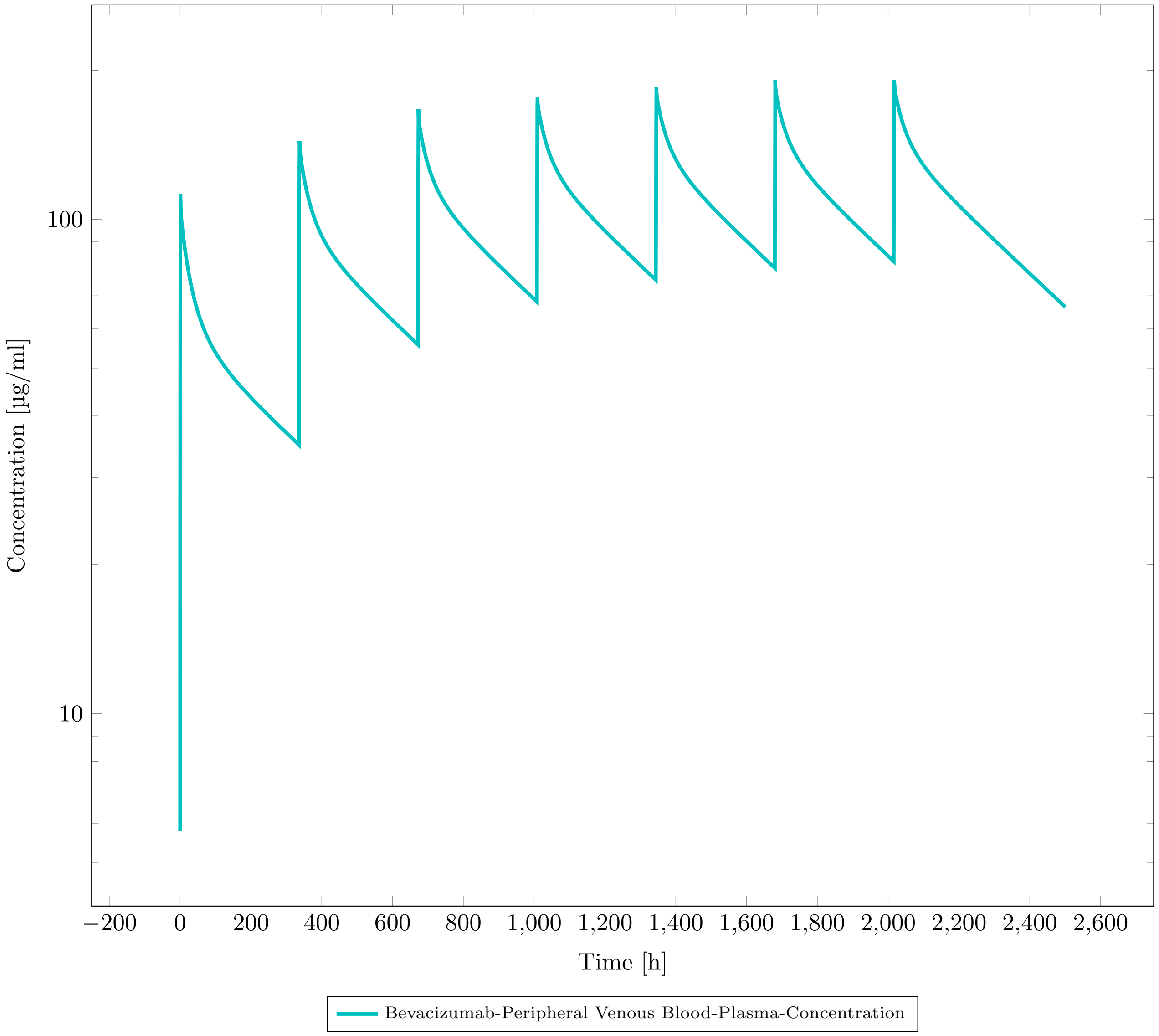


**Figure 7.1**

#### 7.3.1.1 PK-Analyses

**Bevacizumab-Peripheral Venous Blood-Plasma-Concentration**

| Parameter | Value | Unit |
|---|---|---|
| C_max | 1.28 | µmol/l |
| C_max_norm | $5.46 \times 10^6$ | mg/l |
| C_max_tD1-tD2 | 0.75 | µmol/l |
| C_max_tD1-tD2_norm | $2.25 \times 10^7$ | mg/l |
| C_max_tDlast-tEnd | 1.27 | µmol/l |
| C_max_tDLast-tEnd_norm | $3.82 \times 10^7$ | mg/l |
| t_max | 1680.99 | h |
| t_max_tD1-tD2 | 1.00 | h |
| t_max_tDlast-tEnd | 2017.29 | h |

*continued on next page*

| *continued from previous page* | | |
|---|---|---|
| **Parameter** | **Value** | **Unit** |
| C_trough_tD2 | 0.47 | µmol/l |
| C_trough_tDlast | 0.44 | µmol/l |
| AUC_tD1-tD2 | 6810.80 | µmol*min/l |
| AUC_tD1-tD2_norm | $2.04 \times 10^{14}$ | µg*min/l |
| AUC_tDlast-1_tDlast | 15137.61 | µmol*min/l |
| AUC_tDlast-1_tDlast_norm | $4.54 \times 10^{14}$ | µg*min/l |
| AUC_inf_tD1 | 0 | µmol*min/l |
| AUC_inf_tD1_norm | 0 | µg*min/l |
| AUC_inf_tDLast | 36960.51 | µmol*min/l |
| AUC_inf_tDLast_norm | $1.11 \times 10^{15}$ | µg*min/l |
| MRT | NaN | h |
| Half-Life | $-0.50$ | h |
| Half-Life_tDlast-tEnd | 446.60 | h |

**Table 7.5:** PK-Analyses for Bevacizumab-Peripheral Venous Blood-Plasma-Concentration

**Beva_Romera2018_patients_5mpk..Human.iv perfusion.5.Bevacizumab-Bevacizumab-Measurement**

| Parameter | Value | Unit |
|---|---|---|
| C_max | 1.27 | µmol/l |
| C_max_norm | $5.44 \times 10^{6}$ | mg/l |
| t_max | 2022.79 | h |
| C_tEnd | 0.52 | µmol/l |
| AUC_tEnd | 90708.27 | µmol*min/l |
| AUC_tEnd_norm | $3.89 \times 10^{14}$ | µg*min/l |
| AUC_inf | 107670.11 | µmol*min/l |
| AUC_inf_norm | $4.61 \times 10^{14}$ | µg*min/l |
| MRT | 1897.54 | h |
| Half-Life | 376.53 | h |
| % AUC (tlast-$\infty$) | 0.16 | |
| Total body clearance/F | $2.17 \times 10^{-3}$ | ml/min/kg |
| Vss (plasma)/F | 246.73 | ml/kg |
| Vd (plasma)/F | 70.63 | ml/kg |

**Table 7.6:** PK-Analyses for Beva_Romera2018_patients_5mpk..Human.iv perfusion.5.Bevacizumab-Bevacizumab-Measurement

# Chapter 8

# Sim_Patient_0.3mg/kg

## 8.1 Used building blocks

| Building Block | Name |
|---|---|
| Individual | Patient1 (see section 1.3 in Part I) |
| Compound | Bevacizumab (see section 2.2 in Part I) |
| Protocol | Infusion 0.3mg/kg human (see section 3.4 in Part I) |

**Table 8.1:** Building Block

## 8.2 Simulation Properties

### 8.2.1 Model Structure

**Allow aging**
No

**Calculation methods**

| Category | Calculation methods |
|---|---|
| Endothelial surface areas | Organ vascularization |
| Body surface area | Mosteller |

**Table 8.2:** Calculation methods

### 8.2.2 Compounds

#### 8.2.2.1 Bevacizumab

**Compound Configuration**

| Parameter | Alternative in compound | Value | Unit |
|---|---|---|---|
| Solubility | Measurement | 9999.00 | mg/l |
| Lipophilicity | Measurement | −5.00 | Log Units |
| Fraction unbound (plasma, reference value) | Measurement | 1.00 | |

*continued on next page*

| *continued from previous page* | | | |
|---|---|---|---|
| **Parameter** | **Alternative in compound** | **Value** | **Unit** |
| Specific organ permeability | Calculated | 0 | cm/min |
| Specific intestinal permeability | Calculated | 0 | cm/min |

**Table 8.3:** Compound Configuration

**Calculation methods**

| Category | Calculation methods |
|---|---|
| Partition coefficients | PK-Sim Standard |
| Cellular permeabilities | PK-Sim Standard |

**Table 8.4:** Calculation methods

## 8.2.3 Processes

### 8.2.3.1 Bevacizumab

**Specific Binding**
Mapping VEGFA with VEGFA-Paper

## 8.2.4 Administration

### 8.2.4.1 Bevacizumab

**Schema Item 1**
Intravenous Infusion
Start time: 0 h
Dose: 0.30 mg/kg
Infusion time: 90.00 min

**Schema Item 1**
Intravenous Infusion
Start time: 0 h
Dose: 0.30 mg/kg
Infusion time: 60.00 min

**Schema Item 1**
Intravenous Infusion
Start time: 0 h
Dose: 0.30 mg/kg
Infusion time: 60.00 min

**Schema Item 1**
Intravenous Infusion
Start time: 0 h
Dose: 0.30 mg/kg
Infusion time: 60.00 min

## 8.3 Charts

### 8.3.1 Time Profile Analysis

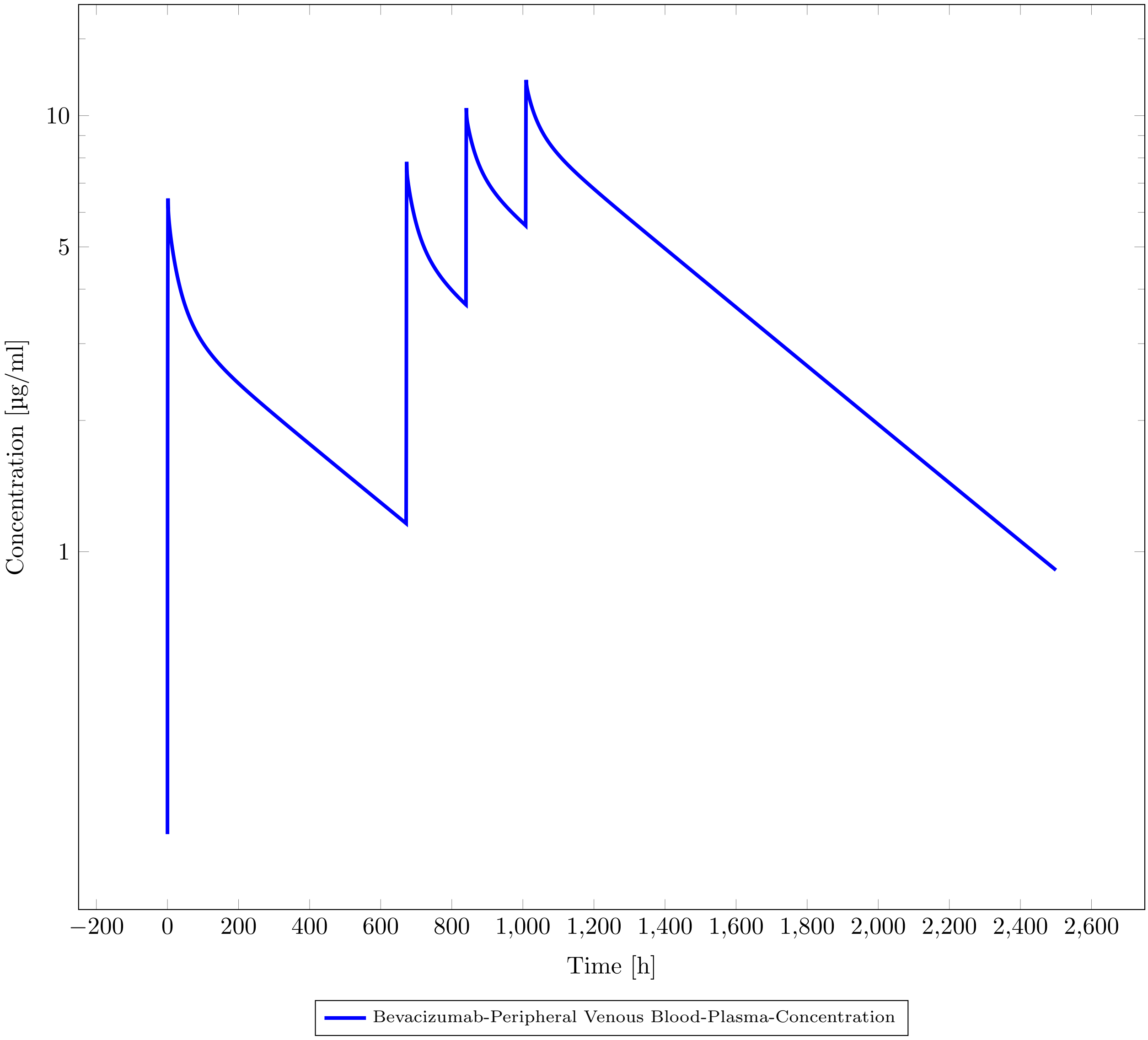


**Figure 8.1**

#### 8.3.1.1 PK-Analyses

**Bevacizumab-Peripheral Venous Blood-Plasma-Concentration**

| Parameter | Value | Unit |
|---|---|---|
| C_max | 0.08 | µmol/l |
| C_max_norm | $1.01 \times 10^7$ | mg/l |
| C_max_tD1-tD2 | 0.04 | µmol/l |
| C_max_tD1-tD2_norm | $2.15 \times 10^7$ | mg/l |
| C_max_tDlast-tEnd | 0.08 | µmol/l |
| C_max_tDLast-tEnd_norm | $4.02 \times 10^7$ | mg/l |
| t_max | 1009.40 | h |
| t_max_tD1-tD2 | 1.50 | h |

*continued on next page*

| *continued from previous page* | | |
|---|---|---|
| **Parameter** | **Value** | **Unit** |
| t_max_tDlast-tEnd | 1009.40 | h |
| C_trough_tD2 | 0.01 | µmol/l |
| C_trough_tDlast | $6.05 \times 10^{-3}$ | µmol/l |
| AUC_tD1-tD2 | 585.81 | µmol*min/l |
| AUC_tD1-tD2_norm | $2.93 \times 10^{14}$ | µg*min/l |
| AUC_tDlast-1_tDlast | 463.83 | µmol*min/l |
| AUC_tDlast-1_tDlast_norm | $2.32 \times 10^{14}$ | µg*min/l |
| AUC_inf_tD1 | 0 | µmol*min/l |
| AUC_inf_tD1_norm | 0 | µg*min/l |
| AUC_inf_tDLast | 2392.41 | µmol*min/l |
| AUC_inf_tDLast_norm | $1.20 \times 10^{15}$ | µg*min/l |
| MRT | NaN | h |
| Half-Life | $-0.73$ | h |
| Half-Life_tDlast-tEnd | 453.27 | h |

**Table 8.5:** PK-Analyses for Bevacizumab-Peripheral Venous Blood-Plasma-Concentration

**Beva_Gordon2001_patients_0.3mpk..Human.iv perfusion.0.3.Bevacizumab-Bevacizumab-Measurement**

| **Parameter** | **Value** | **Unit** |
|---|---|---|
| C_max | 0.07 | µmol/l |
| C_max_norm | $8.38 \times 10^{6}$ | mg/l |
| t_max | 1008.57 | h |
| C_tEnd | $3.12 \times 10^{-3}$ | µmol/l |
| AUC_tEnd | 2314.57 | µmol*min/l |
| AUC_tEnd_norm | $2.89 \times 10^{14}$ | µg*min/l |
| AUC_inf | NaN | µmol*min/l |
| AUC_inf_norm | NaN | µg*min/l |
| MRT | NaN | h |
| Half-Life | Infinity | h |
| % AUC (tlast-$\infty$) | NaN | |
| Total body clearance/F | NaN | ml/min/kg |
| Vss (plasma)/F | NaN | ml/kg |
| Vd (plasma)/F | NaN | ml/kg |

**Table 8.6:** PK-Analyses for Beva_Gordon2001_patients_0.3mpk..Human.iv perfusion.0.3.Bevacizumab-Bevacizumab-Measurement

# Chapter 9

# Sim_patient_ped_5mg/kg

## 9.1 Used building blocks

| Building Block | Name |
|---|---|
| Individual | Patient_ped (see section 1.4 in Part I) |
| Compound | Bevacizumab (see section 2.2 in Part I) |
| Protocol | Infusion_5mg/kg (see section 3.9 in Part I) |

**Table 9.1:** Building Block

## 9.2 Simulation Properties

### 9.2.1 Model Structure

**Allow aging**
No

**Calculation methods**

| Category | Calculation methods |
|---|---|
| Endothelial surface areas | Organ vascularization |
| Body surface area | Mosteller |

**Table 9.2:** Calculation methods

### 9.2.2 Compounds

#### 9.2.2.1 Bevacizumab

**Compound Configuration**

| Parameter | Alternative in compound | Value | Unit |
|---|---|---|---|
| Solubility | Measurement | 9999.00 | mg/l |
| Lipophilicity | Measurement | −5.00 | Log Units |
| Fraction unbound (plasma, reference value) | Measurement | 1.00 | |
| *continued on next page* | | | |

| *continued from previous page* | | | |
|---|---|---|---|
| **Parameter** | **Alternative in compound** | **Value** | **Unit** |
| Specific organ permeability | Calculated | 0 | cm/min |
| Specific intestinal permeability | Calculated | 0 | cm/min |

**Table 9.3:** Compound Configuration

**Calculation methods**

| **Category** | **Calculation methods** |
|---|---|
| Partition coefficients | PK-Sim Standard |
| Cellular permeabilities | PK-Sim Standard |

**Table 9.4:** Calculation methods

### 9.2.3 Processes

#### 9.2.3.1 Bevacizumab

**Specific Binding**
Mapping VEGFA with VEGFA-Paper

### 9.2.4 Administration

#### 9.2.4.1 Bevacizumab

**Simple protocol**
Intravenous Infusion
Dosing interval: Single
Dose: 5.00 mg/kg
Infusion time: 60.00 min

## 9.3 Charts

### 9.3.1 Time Profile Analysis

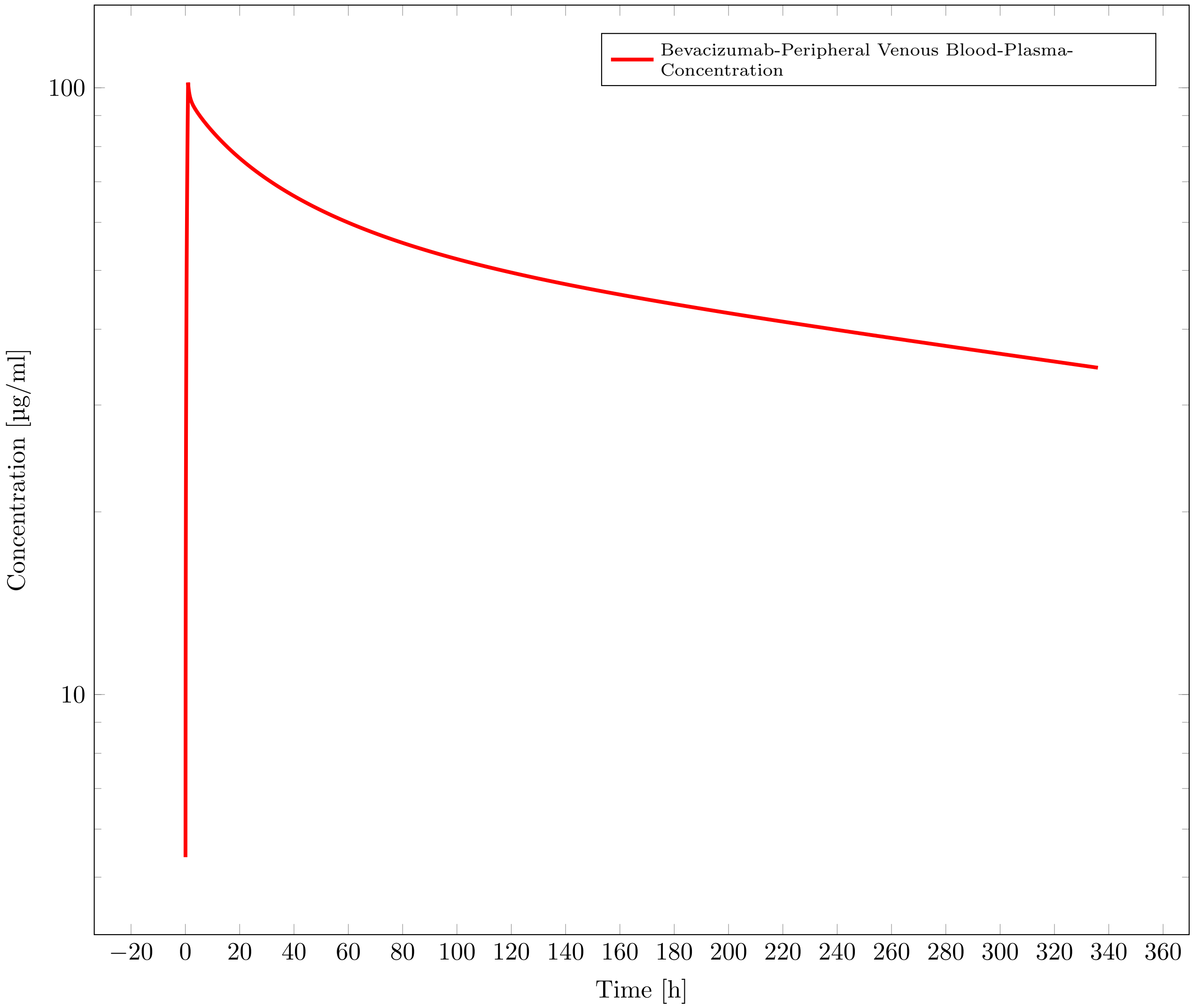


**Figure 9.1**

#### 9.3.1.1 Global PK-Analyses

| Parameter | Compound | Value | Unit |
|---|---|---|---|
| Vss (plasma) | Bevacizumab | 82.20 | ml/kg |
| Vd (plasma) | Bevacizumab | 85.17 | ml/kg |
| Vss (phys-chem) | Bevacizumab | 650.79 | ml/kg |
| Total plasma clearance | Bevacizumab | $2.08 \times 10^{-3}$ | ml/min/kg |

**Table 9.5:** Global PK-Analyses

#### 9.3.1.2 PK-Analyses

**Bevacizumab-Peripheral Venous Blood-Plasma-Concentration**

| Parameter | Value | Unit |
|---|---|---|
| C_max | 0.68 | µmol/l |
| C_max_norm | $2.04 \times 10^{7}$ | mg/l |
| t_max | 1.00 | h |
| C_tEnd | 0.23 | µmol/l |
| AUC_tEnd | 6586.24 | µmol*min/l |
| AUC_tEnd_norm | $1.98 \times 10^{14}$ | µg*min/l |
| AUC_inf | 16013.58 | µmol*min/l |
| AUC_inf_norm | $4.80 \times 10^{14}$ | µg*min/l |
| MRT | 658.14 | h |
| Half-Life | 472.66 | h |
| % AUC (tlast-$\infty$) | 0.59 | |
| Total body clearance/F | $2.08 \times 10^{-3}$ | ml/min/kg |
| Vss (plasma)/F | 82.20 | ml/kg |
| Vd (plasma)/F | 85.17 | ml/kg |

**Table 9.6:** PK-Analyses for Bevacizumab-Peripheral Venous Blood-Plasma-Concentration

# Chapter 10

# Sim_patient_ped_15mg/kg

## 10.1 Used building blocks

| Building Block | Name |
|---|---|
| Individual | Patient_ped (see section 1.4 in Part I) |
| Compound | Bevacizumab (see section 2.2 in Part I) |
| Protocol | Infusion_15mg/kg (see section 3.10 in Part I) |

**Table 10.1:** Building Block

## 10.2 Simulation Properties

### 10.2.1 Model Structure

**Allow aging**
No

**Calculation methods**

| Category | Calculation methods |
|---|---|
| Endothelial surface areas | Organ vascularization |
| Body surface area | Mosteller |

**Table 10.2:** Calculation methods

### 10.2.2 Compounds

#### 10.2.2.1 Bevacizumab

**Compound Configuration**

| Parameter | Alternative in compound | Value | Unit |
|---|---|---|---|
| Solubility | Measurement | 9999.00 | mg/l |
| Lipophilicity | Measurement | −5.00 | Log Units |
| Fraction unbound (plasma, reference value) | Measurement | 1.00 | |
| *continued on next page* | | | |

| *continued from previous page* | | | |
|---|---|---|---|
| **Parameter** | **Alternative in compound** | **Value** | **Unit** |
| Specific organ permeability | Calculated | 0 | cm/min |
| Specific intestinal permeability | Calculated | 0 | cm/min |

**Table 10.3:** Compound Configuration

**Calculation methods**

| **Category** | **Calculation methods** |
|---|---|
| Partition coefficients | PK-Sim Standard |
| Cellular permeabilities | PK-Sim Standard |

**Table 10.4:** Calculation methods

### 10.2.3 Processes

#### 10.2.3.1 Bevacizumab

**Specific Binding**
Mapping VEGFA with VEGFA-Paper

### 10.2.4 Administration

#### 10.2.4.1 Bevacizumab

**Simple protocol**
Intravenous Infusion
Dosing interval: Single
Dose: 15.00 mg/kg
Infusion time: 60.00 min

## 10.3 Charts

### 10.3.1 Time Profile Analysis

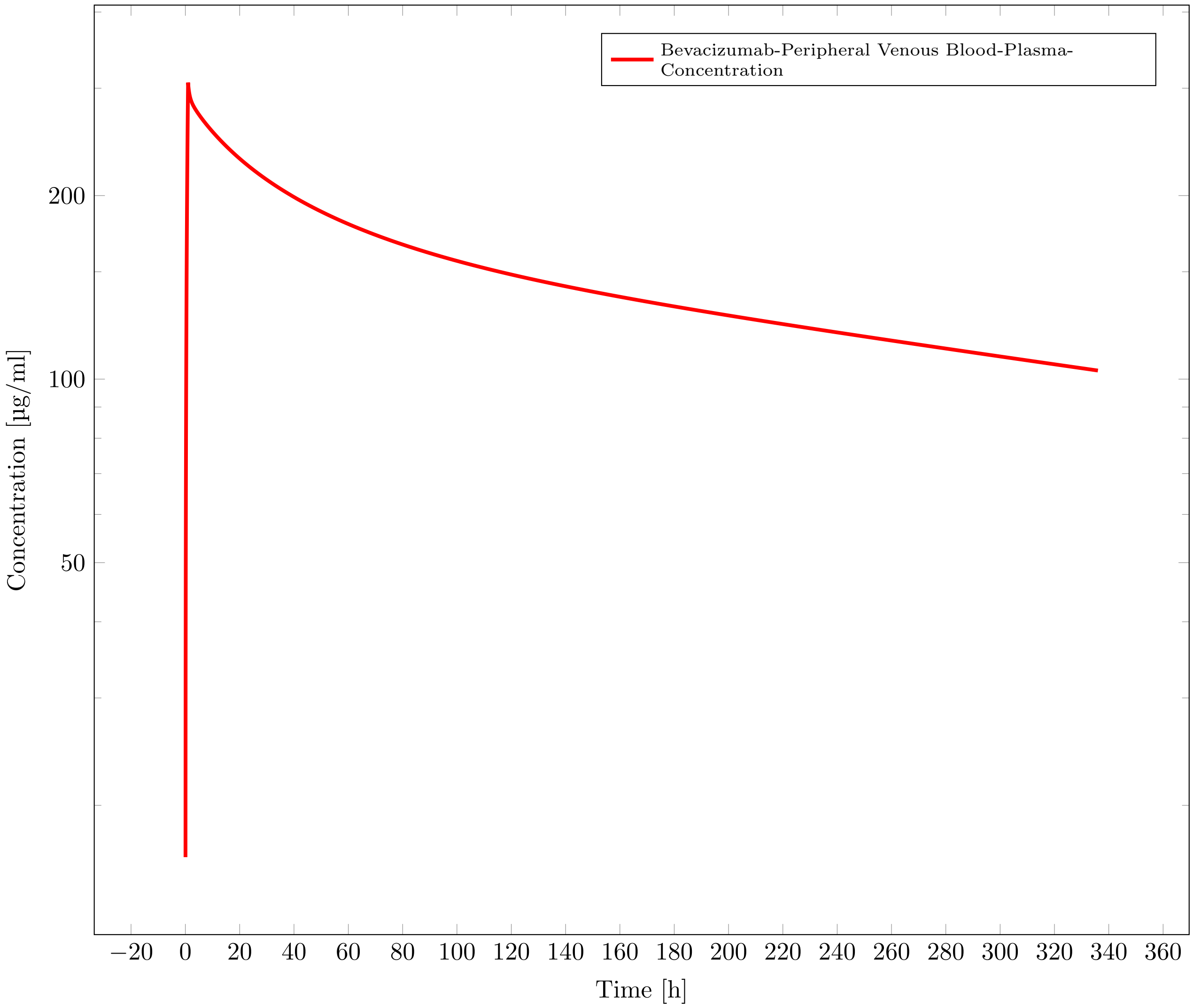


**Figure 10.1**

#### 10.3.1.1 Global PK-Analyses

| Parameter | Compound | Value | Unit |
|---|---|---|---|
| Vss (plasma) | Bevacizumab | 82.32 | ml/kg |
| Vd (plasma) | Bevacizumab | 85.34 | ml/kg |
| Vss (phys-chem) | Bevacizumab | 650.79 | ml/kg |
| Total plasma clearance | Bevacizumab | $2.09 \times 10^{-3}$ | ml/min/kg |

**Table 10.5:** Global PK-Analyses

#### 10.3.1.2 PK-Analyses

### Bevacizumab-Peripheral Venous Blood-Plasma-Concentration

| Parameter | Value | Unit |
|---|---|---|
| C_max | 2.04 | µmol/l |
| C_max_norm | $2.04 \times 10^{7}$ | mg/l |
| t_max | 1.00 | h |
| C_tEnd | 0.69 | µmol/l |
| AUC_tEnd | 19721.20 | µmol*min/l |
| AUC_tEnd_norm | $1.97 \times 10^{14}$ | µg*min/l |
| AUC_inf | 47833.70 | µmol*min/l |
| AUC_inf_norm | $4.78 \times 10^{14}$ | µg*min/l |
| MRT | 656.31 | h |
| Half-Life | 471.61 | h |
| % AUC (tlast-$\infty$) | 0.59 | |
| Total body clearance/F | $2.09 \times 10^{-3}$ | ml/min/kg |
| Vss (plasma)/F | 82.32 | ml/kg |
| Vd (plasma)/F | 85.34 | ml/kg |

**Table 10.6:** PK-Analyses for Bevacizumab-Peripheral Venous Blood-Plasma-Concentration

# Part III

# Observed Data

# Chapter 1

# Beva_Demarchi2021_HV_-1mpk..Human.iv perfusion.1.Bevacizumab_human_sim

Observed Data
Beva_Demarchi2021_HV_1mpk..Human.iv perfusion.1.Bevacizumab_human_sim
Source: J:\PhInC\Suivi_Echanges\Stage\Salih Benamara_2022\Gastro Plus\Bevacizumab_all_Raw_Data\Donne propres\Beva_Demarchi2021_HV_1mpk.csv
Sheet:
Species: Human
Route: iv perfusion
Dose: 1
Molecule: Bevacizumab_human_sim

| **Time [h]** | **Measurement [µg/ml]** |
|---|---|
| 0 | 0.06 |
| 0.75 | 12.00 |
| 4.86 | 23.00 |
| 16.21 | 22.50 |
| 25.93 | 22.00 |
| 43.75 | 19.00 |
| 58.34 | 14.69 |
| 71.30 | 13.08 |
| 95.61 | 12.19 |
| 183.12 | 8.90 |
| 358.14 | 6.06 |
| 526.67 | 5.85 |
| 696.83 | 3.23 |
| 862.12 | 2.47 |
| 1030.65 | 1.84 |
| 1199.19 | 1.41 |
| 1534.64 | 0.76 |
| 1704.79 | 0.49 |
| 1871.71 | 0.27 |
| 2278.46 | 0.01 |

**Table 1.1:** Beva_Demarchi2021_HV_1mpk..Hu

# Chapter 2

# Beva_Hetema2017_HV_-1mpk..Human.iv perfusion.1.Bevacizumab_human_sim

Observed Data
Beva_Hetema2017_HV_1mpk..Human.iv perfusion.1.Bevacizumab_human_sim
Source: J:\PhInC\Suivi_Echanges\Stage\Salih Benamara_2022\Gastro Plus\Bevacizumab_all_Raw_Data\Donne propres\Beva_Hetema2017_HV_1mpk.csv
Sheet:
Species: Human
Route: iv perfusion
Dose: 1
Molecule: Bevacizumab_human_sim

| **Time [h]** | **Measurement [µg/ml]** |
|---|---|
| 0.64 | 24.50 |
| 1.29 | 22.93 |
| 1.93 | 21.48 |
| 2.57 | 21.06 |
| 24.44 | 18.23 |
| 46.95 | 14.39 |
| 91.32 | 11.39 |
| 169.14 | 9.88 |
| 333.78 | 7.08 |
| 502.29 | 5.12 |
| 838.00 | 2.53 |
| 1179.50 | 1.26 |

**Table 2.1:** Beva_Hetema2017_HV_1mpk..Huma

# Chapter 3

# Beva_Hummel2022_HV_-1mpk..Human.iv perfusion.1.Bevacizumab_human_sim

Observed Data
Beva_Hummel2022_HV_1mpk..Human.iv perfusion.1.Bevacizumab_human_sim
Source: J:\PhInC\Suivi_Echanges\Stage\Salih Benamara_2022\Gastro Plus\Bevacizumab_all_Raw_Data\Donne propres\Beva_Hummel2022_HV_1mpk.csv
Sheet:
Species: Human
Route: iv perfusion
Dose: 1
Molecule: Bevacizumab_human_sim

| **Time [h]** | **Measurement [µg/ml]** |
|---|---|
| 0.33 | 5.00 |
| 1.00 | 15.00 |
| 1.50 | 25.00 |
| 2.00 | 24.90 |
| 3.00 | 24.80 |
| 4.00 | 24.60 |
| 5.00 | 24.50 |
| 6.00 | 24.40 |
| 8.00 | 24.20 |
| 20.05 | 24.07 |
| 30.74 | 20.16 |
| 41.43 | 17.65 |
| 60.14 | 15.46 |
| 81.52 | 13.73 |
| 102.90 | 12.39 |
| 134.97 | 11.25 |
| 163.04 | 10.30 |
| 204.46 | 9.50 |
| 273.95 | 8.76 |
| 350.13 | 7.61 |
| 510.49 | 5.22 |
| 672.19 | 4.00 |

*continued on next page*

*continued from previous page*

| **Time [h]** | **Measurement [µg/ml]** |
|---|---|
| 1014.30 | 2.20 |
| 1348.39 | 1.15 |
| 1679.81 | 0.63 |
| 2013.90 | 0.31 |
| 2353.34 | 0.10 |

**Table 3.1:** Beva_Hummel2022_HV_1mpk..Huma

# Chapter 4

# Beva_wu2019_HV_1mpk..Human.iv perfusion.1.Bevacizumab_human_sim.1

Observed Data
Beva_wu2019_HV_1mpk..Human.iv perfusion.1.Bevacizumab_human_sim.1
Source: J:\PhInC\Suivi_Echanges\Stage\Salih Benamara_2022\Gastro Plus\Bevacizumab_all_Raw_Data\Donne propres\Beva_wu2019_HV_1mpk.csv
Sheet:
Species: Human
Route: iv perfusion
Dose: 1
Molecule: Bevacizumab_human_sim
Subject Id: 1

| Time [h] | Measurement [µg/ml] |
|---|---|
| 0.75 | 12.00 |
| 1.50 | 25.00 |
| 2.50 | 24.50 |
| 3.50 | 24.00 |
| 5.50 | 22.50 |
| 9.50 | 20.00 |
| 14.58 | 18.09 |
| 19.92 | 16.04 |
| 46.98 | 13.86 |
| 94.96 | 11.28 |
| 168.77 | 8.42 |
| 237.64 | 7.40 |
| 336.02 | 6.29 |
| 503.26 | 4.86 |
| 671.74 | 3.69 |
| 840.22 | 2.62 |
| 1007.47 | 1.97 |
| 1344.42 | 1.13 |
| 1680.16 | 0.60 |
| 2015.89 | 0.31 |
| 2351.62 | 0.18 |

**Table 4.1:** Beva_wu2019_HV_1mpk..Human.iv

# Chapter 5

# Beva_wu2019_HV_1mpk..Human.iv perfusion.1.Bevacizumab_human_sim.2

Observed Data
Beva_wu2019_HV_1mpk..Human.iv perfusion.1.Bevacizumab_human_sim.2
Source: J:\PhInC\Suivi_Echanges\Stage\Salih Benamara_2022\Gastro Plus\Bevacizumab_all_Raw_Data\Donne propres\Beva_wu2019_HV_1mpk.csv
Sheet:
Species: Human
Route: iv perfusion
Dose: 1
Molecule: Bevacizumab_human_sim
Subject Id: 2

| Time [h] | Measurement [µg/ml] |
|---|---|
| 14.58 | 18.09 |
| 19.92 | 16.04 |
| 46.98 | 13.86 |
| 94.96 | 11.28 |
| 168.77 | 8.42 |
| 237.64 | 7.40 |
| 336.02 | 4.99 |
| 503.26 | 3.92 |
| 671.74 | 2.98 |
| 840.22 | 2.04 |
| 1007.47 | 1.40 |
| 1344.42 | 0.74 |
| 1680.16 | 0.30 |
| 2015.89 | 0.10 |
| 2351.62 | 0.03 |

**Table 5.1:** Beva_wu2019_HV_1mpk..Human.iv

# Chapter 6

# Beva_wu2019_HV_1mpk..Human.iv perfusion.1.Bevacizumab_human_sim.3

Observed Data
Beva_wu2019_HV_1mpk..Human.iv perfusion.1.Bevacizumab_human_sim.3
Source: J:\PhInC\Suivi_Echanges\Stage\Salih Benamara_2022\Gastro Plus\Bevacizumab_all_Raw_Data\Donne propres\Beva_wu2019_HV_1mpk.csv
Sheet:
Species: Human
Route: iv perfusion
Dose: 1
Molecule: Bevacizumab_human_sim
Subject Id: 3

| **Time [h]** | **Measurement [µg/ml]** |
|---|---|
| 14.58 | 18.09 |
| 19.92 | 16.04 |
| 46.98 | 13.86 |
| 94.96 | 11.28 |
| 168.77 | 8.42 |
| 237.64 | 7.40 |
| 336.02 | 7.66 |
| 503.26 | 5.92 |
| 671.74 | 4.35 |
| 840.22 | 3.14 |
| 1007.47 | 2.26 |
| 1344.42 | 1.35 |
| 1680.16 | 0.65 |
| 2015.89 | 0.53 |
| 2351.62 | 0.37 |

**Table 6.1:** Beva_wu2019_HV_1mpk..Human.iv

# Chapter 7

# Beva_shin2020_HV_3mpk..Human.iv perfusion.3.Bevacizumab_human_sim.1

Observed Data
Beva_shin2020_HV_3mpk..Human.iv perfusion.3.Bevacizumab_human_sim.1
Source: J:\PhInC\Suivi_Echanges\Stage\Salih Benamara_2022\Gastro Plus\Bevacizumab_all_Raw_Data\Donne propres\Beva_shin2020_HV_3mpk.csv
Sheet:
Species: Human
Route: iv perfusion
Dose: 3
Molecule: Bevacizumab_human_sim
Subject Id: 1

| **Time [h]** | **Measurement [µg/ml]** |
|---|---|
| 0.75 | 33.20 |
| 1.50 | 75.45 |
| 4.15 | 74.26 |
| 8.30 | 65.68 |
| 12.45 | 59.41 |
| 24.90 | 55.56 |
| 47.73 | 49.14 |
| 96.49 | 40.42 |
| 168.09 | 31.80 |
| 335.14 | 23.01 |
| 506.33 | 18.61 |
| 672.35 | 14.89 |
| 1006.44 | 9.21 |
| 1344.69 | 5.83 |
| 1679.83 | 3.69 |
| 2016.00 | 2.14 |

**Table 7.1:** Beva_shin2020_HV_3mpk..Human.

## Chapter 8

# Beva_shin2020_HV_3mpk..Human.iv perfusion.3.Bevacizumab_human_sim.2

Observed Data
Beva_shin2020_HV_3mpk..Human.iv perfusion.3.Bevacizumab_human_sim.2
Source: J:\PhInC\Suivi_Echanges\Stage\Salih Benamara_2022\Gastro Plus\Bevacizumab_all_Raw_Data\Donne propres\Beva_shin2020_HV_3mpk.csv
Sheet:
Species: Human
Route: iv perfusion
Dose: 3
Molecule: Bevacizumab_human_sim
Subject Id: 2

| Time [h] | Measurement [µg/ml] |
|---|---|
| 47.73 | 41.10 |
| 96.49 | 33.25 |
| 168.09 | 25.72 |
| 335.14 | 18.61 |
| 506.33 | 15.06 |
| 672.35 | 11.14 |
| 1006.44 | 6.59 |
| 1344.69 | 3.90 |
| 1679.83 | 2.11 |
| 2016.00 | 1.17 |

**Table 8.1:** Beva_shin2020_HV_3mpk..Human.

# Chapter 9

# Beva_shin2020_HV_3mpk..Human.iv perfusion.3.Bevacizumab_human_sim.3

Observed Data
Beva_shin2020_HV_3mpk..Human.iv perfusion.3.Bevacizumab_human_sim.3
Source: J:\PhInC\Suivi_Echanges\Stage\Salih Benamara_2022\Gastro Plus\Bevacizumab_all_Raw_Data\Donne propres\Beva_shin2020_HV_3mpk.csv
Sheet:
Species: Human
Route: iv perfusion
Dose: 3
Molecule: Bevacizumab_human_sim
Subject Id: 3

| **Time [h]** | **Measurement [µg/ml]** |
|---|---|
| 47.73 | 58.75 |
| 96.49 | 45.96 |
| 168.09 | 37.59 |
| 335.14 | 27.20 |
| 506.33 | 22.50 |
| 672.35 | 18.61 |
| 1006.44 | 12.32 |
| 1344.69 | 7.79 |
| 1679.83 | 5.33 |
| 2016.00 | 3.21 |

**Table 9.1:** Beva_shin2020_HV_3mpk..Human.

# Chapter 10

# Beva_sinn2021_HV_3mpk..Human.iv perfusion.3.Bevacizumab_human_sim

Observed Data
Beva_sinn2021_HV_3mpk..Human.iv perfusion.3.Bevacizumab_human_sim
Source: J:\PhInC\Suivi_Echanges\Stage\Salih Benamara_2022\Gastro Plus\Bevacizumab_all_Raw_Data\Donne propres\Beva_sinn2021_HV_3mpk.csv
Sheet:
Species: Human
Route: iv perfusion
Dose: 3
Molecule: Bevacizumab_human_sim

| **Time [h]** | **Measurement [µg/ml]** |
|---|---|
| 0.94 | 83.47 |
| 2.00 | 83.00 |
| 3.00 | 82.50 |
| 4.00 | 82.00 |
| 5.00 | 81.00 |
| 6.00 | 80.50 |
| 8.00 | 76.00 |
| 19.75 | 73.15 |
| 36.67 | 64.10 |
| 52.65 | 57.75 |
| 78.04 | 51.32 |
| 106.25 | 49.56 |
| 132.57 | 46.24 |
| 159.84 | 43.43 |
| 184.29 | 41.08 |
| 222.84 | 38.06 |
| 301.82 | 33.59 |
| 473.88 | 24.06 |
| 639.37 | 17.84 |
| 984.44 | 10.60 |
| 1307.88 | 6.38 |
| 1840.06 | 2.70 |
| 2370.36 | 1.03 |

*continued on next page*

| *continued from previous page* | |
|---|---|
| **Time [h]** | **Measurement [µg/ml]** |

**Table 10.1:** Beva_sinn2021_HV_3mpk..Human.

# Chapter 11

# Beva_li2017_HV_0.5mpk..Human.iv perfusion.0.5.Bevacizumab_human_-sim.1

Observed Data
Beva_li2017_HV_0.5mpk..Human.iv perfusion.0.5.Bevacizumab_human_sim.1
Source: J:\PhInC\Suivi_Echanges\Stage\Salih Benamara_2022\Gastro Plus\Bevacizumab_all_Raw_Data\Donne propres\Beva_li2017_HV_0.5mpk.csv
Sheet:
Species: Human
Route: iv perfusion
Dose: 0.5
Molecule: Bevacizumab_human_sim
Subject Id: 1

| Time [h] | Measurement [mg/l] |
|---|---|
| 2.00 | 6.74 |
| 6.00 | 6.50 |
| 16.73 | 6.30 |
| 24.00 | 6.00 |
| 39.03 | 4.70 |
| 64.13 | 4.55 |
| 114.31 | 3.64 |
| 161.71 | 3.40 |
| 329.00 | 2.45 |
| 663.57 | 1.32 |
| 833.64 | 0.86 |
| 1000.93 | 0.50 |
| 1168.22 | 0.25 |
| 1335.50 | 0.15 |

**Table 11.1:** Beva_li2017_HV_0.5mpk..Human.

# Chapter 12

# Beva_li2017_HV_0.5mpk..Human.iv perfusion.0.5.Bevacizumab_human_-sim.2

Observed Data
Beva_li2017_HV_0.5mpk..Human.iv perfusion.0.5.Bevacizumab_human_sim.2
Source: J:\PhInC\Suivi_Echanges\Stage\Salih Benamara_2022\Gastro Plus\Bevacizumab_all_Raw_Data\Donne propres\Beva_li2017_HV_0.5mpk.csv
Sheet:
Species: Human
Route: iv perfusion
Dose: 0.5
Molecule: Bevacizumab_human_sim
Subject Id: 2

| **Time [h]** | **Measurement [mg/l]** |
|---|---|
| 2.00 | 5.58 |
| 8.38 | 4.62 |
| 12.00 | 4.70 |
| 24.00 | 4.50 |
| 39.11 | 3.58 |
| 64.25 | 3.28 |
| 111.73 | 3.01 |
| 162.01 | 2.10 |
| 326.82 | 1.37 |
| 662.01 | 0.64 |
| 829.61 | 0.45 |
| 1000.00 | 0.23 |
| 1170.39 | 0.10 |
| 1337.99 | 0.08 |

**Table 12.1:** Beva_li2017_HV_0.5mpk..Human.

# Chapter 13

# Beva_li2017_HV_0.5mpk..Human.iv perfusion.0.5.Bevacizumab_human_sim.3

Observed Data
Beva_li2017_HV_0.5mpk..Human.iv perfusion.0.5.Bevacizumab_human_sim.3
Source: J:\PhInC\Suivi_Echanges\Stage\Salih Benamara_2022\Gastro Plus\Bevacizumab_all_Raw_Data\Donne propres\Beva_li2017_HV_0.5mpk.csv
Sheet:
Species: Human
Route: iv perfusion
Dose: 0.5
Molecule: Bevacizumab_human_sim
Subject Id: 3

| **Time [h]** | **Measurement [mg/l]** |
|---|---|
| 2.00 | 8.14 |
| 5.50 | 7.52 |
| 19.27 | 6.40 |
| 24.00 | 6.30 |
| 41.28 | 6.19 |
| 66.06 | 5.21 |
| 112.84 | 4.92 |
| 162.39 | 4.01 |
| 330.28 | 3.26 |
| 663.30 | 1.35 |
| 833.94 | 0.90 |
| 999.08 | 0.51 |
| 1166.97 | 0.21 |
| 1337.61 | 0.11 |

**Table 13.1:** Beva_li2017_HV_0.5mpk..Human.

# Chapter 14

# Beva_li2017_HV_0.5mpk..Human.iv perfusion.0.5.Bevacizumab_human_-sim.4

Observed Data
Beva_li2017_HV_0.5mpk..Human.iv perfusion.0.5.Bevacizumab_human_sim.4
Source: J:\PhInC\Suivi_Echanges\Stage\Salih Benamara_2022\Gastro Plus\Bevacizumab_all_Raw_Data\Donne propres\Beva_li2017_HV_0.5mpk.csv
Sheet:
Species: Human
Route: iv perfusion
Dose: 0.5
Molecule: Bevacizumab_human_sim
Subject Id: 4

| Time [h] | Measurement [mg/l] |
|---|---|
| 2.00 | 7.52 |
| 5.51 | 6.26 |
| 13.79 | 6.05 |
| 24.00 | 6.00 |
| 41.36 | 4.76 |
| 66.18 | 4.55 |
| 113.05 | 4.01 |
| 162.68 | 3.42 |
| 330.88 | 2.51 |
| 664.52 | 1.18 |
| 835.48 | 0.88 |
| 1000.92 | 0.71 |
| 1169.12 | 0.36 |
| 1337.32 | 0.18 |

**Table 14.1:** Beva_li2017_HV_0.5mpk..Human.

# Chapter 15

# Beva_li2017_HV_0.5mpk..Human.iv perfusion.0.5.Bevacizumab_human_-sim.5

Observed Data
Beva_li2017_HV_0.5mpk..Human.iv perfusion.0.5.Bevacizumab_human_sim.5
Source: J:\PhInC\Suivi_Echanges\Stage\Salih Benamara_2022\Gastro Plus\Bevacizumab_all_Raw_Data\Donne propres\Beva_li2017_HV_0.5mpk.csv
Sheet:
Species: Human
Route: iv perfusion
Dose: 0.5
Molecule: Bevacizumab_human_sim
Subject Id: 5

| Time [h] | Measurement [mg/l] |
|---|---|
| 2.00 | 6.85 |
| 2.75 | 5.70 |
| 19.27 | 5.32 |
| 24.00 | 5.20 |
| 41.28 | 4.74 |
| 66.06 | 3.95 |
| 115.60 | 3.40 |
| 165.14 | 3.03 |
| 330.28 | 1.89 |
| 663.30 | 0.98 |
| 833.94 | 0.71 |
| 1001.83 | 0.59 |
| 1169.72 | 0.36 |
| 1334.86 | 0.16 |

**Table 15.1:** Beva_li2017_HV_0.5mpk..Human.

# Chapter 16

# Beva_Gordon2001_patients_-3mpk..Human.iv perfusion.3.Bevacizumab

Observed Data
Beva_Gordon2001_patients_3mpk..Human.iv perfusion.3.Bevacizumab
Source: J:\PhInC\Suivi_Echanges\Stage\Salih Benamara_2022\Gastro Plus\Bevacizumab_all_Raw_Data\Donne propres\Beva_Gordon2001_patients_3mpk.csv
Sheet:
Species: Human
Route: iv perfusion
Dose: 3
Molecule: Bevacizumab

| **Time [h]** | **Measurement [µg/ml]** |
|---|---|
| 2.83 | 77.29 |
| 17.00 | 63.47 |
| 45.33 | 58.82 |
| 90.66 | 44.09 |
| 161.48 | 32.55 |
| 235.14 | 29.70 |
| 334.30 | 26.29 |
| 504.28 | 17.70 |
| 671.43 | 13.66 |
| 674.27 | 92.14 |
| 835.75 | 44.47 |
| 841.42 | 134.36 |
| 1005.73 | 57.44 |
| 1008.57 | 170.96 |
| 1056.73 | 110.13 |
| 1172.88 | 70.90 |
| 1345.70 | 44.94 |
| 1507.18 | 28.49 |
| 1728.16 | 21.64 |

**Table 16.1:** Beva_Gordon2001_patients_3mpk

# Chapter 17

# Beva_Gordon2001_patients_-0.3mpk..Human.iv perfusion.0.3.Bevacizumab

Observed Data
Beva_Gordon2001_patients_0.3mpk..Human.iv perfusion.0.3.Bevacizumab
Source: J:\PhInC\Suivi_Echanges\Stage\Salih Benamara_2022\Gastro Plus\Bevacizumab_all_Raw_Data\Donne propres\Beva_Gordon2001_patients_0.3mpk.csv
Sheet:
Species: Human
Route: iv perfusion
Dose: 0.3
Molecule: Bevacizumab

| Time [h] | Measurement [µg/ml] |
|---|---|
| 5.67 | 6.07 |
| 19.83 | 5.21 |
| 45.33 | 4.28 |
| 90.66 | 3.02 |
| 161.48 | 2.33 |
| 235.14 | 1.75 |
| 331.47 | 1.31 |
| 501.45 | 0.72 |
| 671.00 | 0.73 |
| 672.00 | 7.57 |
| 835.75 | 2.91 |
| 841.42 | 9.92 |
| 1005.73 | 3.99 |
| 1008.57 | 10.06 |
| 1056.73 | 8.91 |
| 1172.88 | 5.00 |
| 1342.87 | 2.94 |
| 1510.02 | 1.75 |
| 1728.16 | 0.47 |

**Table 17.1:** Beva_Gordon2001_patients_0.3m

# Chapter 18

# Beva_Gordon2001_patients_-1mpk..Human.iv perfusion.1.Bevacizumab

Observed Data
Beva_Gordon2001_patients_1mpk..Human.iv perfusion.1.Bevacizumab
Source: J:\PhInC\Suivi_Echanges\Stage\Salih Benamara_2022\Gastro Plus\Bevacizumab_all_Raw_Data\Donne propres\Beva_Gordon2001_patients_1mpk.csv
Sheet:
Species: Human
Route: iv perfusion
Dose: 1
Molecule: Bevacizumab

| **Time [h]** | **Measurement [µg/ml]** |
|---|---|
| 2.83 | 24.82 |
| 19.80 | 21.65 |
| 45.25 | 17.25 |
| 90.51 | 13.13 |
| 161.21 | 10.15 |
| 234.75 | 7.85 |
| 330.91 | 6.16 |
| 500.61 | 4.28 |
| 667.47 | 2.64 |
| 670.30 | 27.59 |
| 837.17 | 11.81 |
| 840.00 | 39.69 |
| 1004.04 | 18.33 |
| 1006.87 | 45.49 |
| 1054.95 | 31.62 |
| 1170.91 | 26.77 |
| 1340.61 | 16.74 |
| 1507.47 | 12.18 |
| 1725.25 | 8.99 |

**Table 18.1:** Beva_Gordon2001_patients_1mpk

# Chapter 19

# Beva_Gordon2001_patients_-10mpk..Human.iv perfusion.10.Bevacizumab

Observed Data
Beva_Gordon2001_patients_10mpk..Human.iv perfusion.10.Bevacizumab
Source: J:\PhInC\Suivi_Echanges\Stage\Salih Benamara_2022\Gastro Plus\Bevacizumab_all_Raw_Data\Donne propres\Beva_Gordon2001_patients_10mpk.csv
Sheet:
Species: Human
Route: iv perfusion
Dose: 10
Molecule: Bevacizumab

| Time [h] | Measurement [µg/ml] |
|---|---|
| 2.83 | 233.57 |
| 22.63 | 183.30 |
| 48.08 | 177.83 |
| 90.51 | 135.39 |
| 164.04 | 109.52 |
| 237.58 | 95.56 |
| 333.74 | 76.13 |
| 500.61 | 52.13 |
| 667.47 | 36.80 |
| 673.13 | 284.41 |
| 834.34 | 114.61 |
| 840.00 | 379.27 |
| 1004.04 | 188.94 |
| 1006.87 | 428.13 |
| 1054.95 | 396.90 |
| 1173.74 | 226.60 |
| 1343.43 | 175.15 |
| 1507.47 | 119.94 |
| 1725.25 | 91.31 |

**Table 19.1:** Beva_Gordon2001_patients_10mp

# Chapter 20

# Beva_Romera2018_patients_-5mpk..Human.iv perfusion.5.Bevacizumab

Observed Data
Beva_Romera2018_patients_5mpk..Human.iv perfusion.5.Bevacizumab
Source: J:\PhInC\Suivi_Echanges\Stage\Salih Benamara_2022\Gastro Plus\Bevacizumab_all_Raw_Data\Donne propres\Beva_Romera2018_patients_5mpk.csv
Sheet:
Species: Human
Route: iv perfusion
Dose: 5
Molecule: Bevacizumab

| Time [h] | Measurement [µg/ml] |
|---|---|
| 0.82 | 97.77 |
| 1.64 | 108.28 |
| 6.36 | 114.33 |
| 24.82 | 87.26 |
| 49.03 | 71.18 |
| 73.23 | 64.81 |
| 121.23 | 50.00 |
| 169.03 | 43.47 |
| 241.64 | 35.35 |
| 338.46 | 30.41 |
| 2016.62 | 173.72 |
| 2017.44 | 181.63 |
| 2022.79 | 190.56 |
| 2040.47 | 160.97 |
| 2064.94 | 146.43 |
| 2089.20 | 134.18 |
| 2138.35 | 118.37 |
| 2186.47 | 106.12 |
| 2257.20 | 93.37 |
| 2354.47 | 78.06 |

**Table 20.1:** Beva_Romera2018_patients_5mpk
